# Decoding clone evolution in HER2 amplified breast cancer through single-cell and spatial transcriptomics analysis of copy number variations

**DOI:** 10.1038/s41598-026-44476-7

**Published:** 2026-03-16

**Authors:** Jiao Yang, Yong Li, Suxia Luo, Jian Wang, Yuanqiang Duan

**Affiliations:** 1 Department of Medical Oncology, the Affiliated Cancer Hospital of Zhengzhou University & Henan Cancer Hospital, Zhengzhou, 450008 China; 2 Department of Oncology, the Second Affiliated Hospital of Zhengzhou University, Zhengzhou, 450008 China; 3 Department of Breast Disease, Henan Breast Cancer Center, the Affiliated Cancer Hospital of Zhengzhou University & Henan Cancer Hospital, Zhengzhou, 450008 China; 4 Department of Thoracic Surgery, the Affiliated Cancer Hospital of Zhengzhou University & Henan Cancer Hospital, Zhengzhou, Henan, 450008 China

**Keywords:** HER2-positive breast cancer, ductal carcinoma in situ, invasive ductal carcinoma, copy number variation, clonal evolution., Cancer, Computational biology and bioinformatics, Genetics, Oncology

## Abstract

HER2-positive breast cancer exhibits marked genomic instability and heterogeneity, yet the clonal architecture and copy number variation (CNV) dynamics between ductal carcinoma in situ (DCIS) and invasive ductal carcinoma (IDC), remain poorly understood. We analyzed single-cell RNA sequencing data from 14 HER2-positive breast cancer patients and spatial transcriptomics from 8 patients. CNVs were inferred to evaluate genomic alterations and reconstruct tumor subclone evolutionary trajectories. Survival analyses were performed on CNV-correlated transcripts. We identified 68,064 cells and 4,764 spatial transcriptomic spots, and observed early and pervasive CNV events in DCIS. IDC exhibiting a higher CNV burden supported the hypothesis of progressive genomic instability during tumor evolution. Shared CNV regions across DCIS and IDC suggested the common clonal origin, favoring a multi-threaded evolutionary model. Amplifications in chromosome 17q12-21 were associated with poor prognosis. CNV-driven clonal evolution probably originates at early stages of HER2-positive breast cancer and persists through disease progression. Early CNV events may serve as predictive biomarkers and potential intervention targets to prevent disease advancement.

**Supplementary Information:**

The online version contains supplementary material available at 10.1038/s41598-026-44476-7.

## Introduction

Copy number variation (CNV), a form of genomic structural alteration involving segmental duplication or deletion of DNA, plays a significant role in the development and progression of breast cancer^1,2^. In breast cancer, CNVs have been closely linked to tumor initiation, heterogeneity, progression, and therapeutic response^3^. Notably, CNV landscapes in breast cancer are highly heterogeneous, varying not only between molecular subtypes but also between individual patients. In HER2-positive (HER2+) breast cancer, CNVs are reported to mainly contribute to tumor aggressiveness and metastasis^4^.

Ductal carcinoma in situ (DCIS) is considered a potential precursor to invasive ductal carcinoma (IDC). Since only an estimated 10–50% of untreated DCIS cases progress to IDC, overtreatment remains a significant concern^5–8^. Despite numerous efforts, the molecular mechanisms underlying DCIS-to-IDC progression remain poorly understood^6,9^. This underscores the urgent need for reliable prognostic biomarkers to distinguish indolent from high-risk lesions.

Recent advances in single-cell RNA sequencing (scRNA-seq) and spatial transcriptomics have enabled CNV inference at unprecedented resolution, offering insight into CNV patterns at both the cellular and spatial levels^1,10,11^. These technologies allow for the exploration of intratumoral heterogeneity, subclonal architecture, and evolutionary dynamics in situ. In this study, we applied these cutting-edge approaches to characterize CNV landscapes in HER2-positive DCIS and IDC breast cancer. Moreover, by comparing DCIS and IDC, we investigated the distinct CNV profiles and their implications for disease progression, therapeutic resistance, and prognosis in HER2-positive breast cancer.

## Methods

### Data Sources

This study included public available data from four major sources^4,5,10,12^, comprising single-cell RNA sequencing (scRNA-seq) data from 14 HER2-positive breast cancer patients and spatial transcriptomics sequencing (ST-seq) data from 8 patients. The scRNA-seq datasets were obtained from the Broad Institute Single Cell Portal (SCP1039) (*n* = 4), GEO Series GSE161529 (*n* = 6), and NCBI GEO under accession numbers GSE195861 and GSE196208 (*n* = 4). All scRNA-seq data were generated using the 10x Genomics Chromium platform. The ST-seq dataset was achieved from zenodo (4751624) (*n* = 8). All cases were pathologically confirmed as HER2-enriched breast cancer based on immunohistochemistry (IHC) or fluorescence in situ hybridization (FISH), as reported in the original publications. Detailed clinical and pathological information for each patient was provided in Supplementary Table 1.

### Pre-processing and quality control of scRNA-seq data

Filtered gene expression matrix for each sample were obtained from the sources described above and processed using R software (v4.3.1) with the Seurat package (v4.3.0.1). To ensure high data quality, a series of filtering steps were applied. For each sample, cells were excluded if they met any of the following criteria: fewer than 200 unique molecular identifiers (UMIs), fewer than 200 or more than 6,000 detected genes, or more than 20% of UMIs derived from mitochondrial transcripts-indicative of low-quality or dying cells. After quality control, 68,064 high-quality cells from 14 scRNA-seq samples were retained for downstream analysis. Similarly, after applying the same quality filtering criteria, 4764 spots from 8 ST-seq samples were included in subsequent analyses.

### Integrative analysis of multiple datasets

To perform dimensionality reduction, the 2,000 most variable genes were selected using the FindVariableFeatures function with the vst method in the Seurat package. These highly variable genes were then subjected to principal component analysis for linear dimensionality reduction. To correct for batch effects across different samples, the Harmony package (v0.1.1) was employed using the RunHarmony function. Subsequently, cell clustering was performed using Seurat’s FindNeighbors and FindClusters functions. The resolution parameter was adjusted to 0.1 to delineate clusters. Specifically, the top 30 principal components (PCs) were used to construct a shared nearest-neighbor (SNN) graph by calculating the neighborhood overlap between each cell and its nearest neighbors. Clusters were identified using a modularity optimization-based clustering algorithm applied to the SNN graph. For visualization, uniform manifold approximation and projection (UMAP) was conducted on the top 30 PCs using the RunUMAP function, enabling two-dimensional representation of the cellular landscape and cluster distribution.

### Cell clusters annotation

Cell clusters were annotated based on the expression of canonical marker genes corresponding to known cell types. Specifically, the fibroblast cells were identified by expression of PDGFRA, COL3A1, POSTN, COL1A1 and COL5A2; endothelial cells by PECAM1, CLDN5, ITGA6, ENG, FLT1, CD93, PLVAP; myeloid cells by CD68, APOE, APOC1, C1QA and C1QC; B cells by BANK1, CD79A, IGHM, and MS4A1; T cells by CD3D, CLL5, TRBC2, CD2.

Epithelial cells were further refined using SingleR-based (v.2.2.0) annotation. Following epithelial cell selection, standard preprocessing steps, including normalization, dimensionality reduction, and clustering, were repeated. Due to the biological heterogeneity inherent to tumors, batch correction (via Harmony) was not applied in this step. Dimensionality reduction was visualized using the RunUMAP function, and epithelial marker expression was used to validate the epithelial cell clusters.

### InferCNV analysis and phylogenetic trees construction

CNV signals at the single-cell level were estimated using the inferCNV^13^(v1.16.0) with cutoff value 0.1, and a dynamic threshold of 1.5 standard deviations was applied for signal denoising^14^. Required input files, including raw count matrices, cell annotations, and gene / chromosome position data, were prepared following official guidelines (https://github.com/broadinstitute/inferCNV). Immune and endothelial cells, particularly T cells, were used as the primary reference population for CNV normalization. To assess potential reference bias, normal epithelial cells from adjacent non-tumor tissues were also utilized. Comparable CNV patterns were observed with both reference populations, demonstrating the robustness of the inferred CNV profiles. For each sample, gene expression values of individual cells were re-standardized and scaled to a range between − 1 and 1. A CNV score for each cell was calculated as the sum of all CNV regions. Gene-level CNV inferences were derived using a Hidden Markov Model (HMM)-based approach, specifically from the output file HMM_CNV_predictions.HMMi6.rand_trees.hmm_mode-subclusters.Pnorm_0.5.pred_cnv_regions.dat. These inferred CNVs were then visualized using UPhyloplot2 to construct phylogenetic trees, thereby illustrating clonal architecture and evolutionary trajectories of the tumor.

### Identification of tumor cells in scRNA-seq data

A clustering approach analogous to the K-means algorithm was employed to classify epithelial cells in order to distinguish tumor cells from unassigned cells^15^. The cluster number (k = 3) was optimized to balance cluster distinctness, population size, and biological interpretability. For each sample, epithelial cells were grouped into three main clusters based on their CNV scores. The cluster with the highest average CNV score was inferred to contain tumor cells. The remaining clusters were designated as unassigned. This strategy was designed to selectively identify tumor cells with high confidence and preserve the inherent heterogeneity of tumor populations.

### Venn diagram analysis

CNV-altered genes and regions were extracted from single-cell RNA-seq and spatial transcriptomics datasets for each patient sample. With the help of the ggVennDiagram package, shared and unique CNV-altered genes and regions across samples were identified and represented in Venn diagrams to highlight overlaps and distinctions in CNV profiles among different samples.

### Spatial transcriptomics data analysis

Raw spatial transcriptomics data and code were obtained as described in the original publication^4^ and repository (https://github.com/almaan/her2st).

### Definition of epithelial spots signature in ST-seq

Epithelial spot signatures were quantified using the AddModuleScore function in the Seurat package, based on the expression of canonical epithelial markers: EPCAM, KRT8, and KRT18.

### Manual algorithmic tree construction from inferCNV outputs

#### Clone tree consensus spatial inferred CNV (siCNV) event calling

Putative subclonal CNV events were identified by integrating both the HMM-based outputs (from files infercnv.17_HMM_predHMMi6.hmm_mode-samples.png and 17_HMM_predHMMi6.hmm_mode-samples.pred_cnv_regions.dat), along with manual curation of CNV profiles. A final consensus CNV event list was generated for each clone and used to construct clonal phylogenies. Clone trees were built by identifying CNV events shared across cell clusters, based on the biological assumption that CNV events were irreversible. Thus, shared CNVs suggest common ancestry among the clusters. This framework allowed for the hierarchical reconstruction of subclonal lineages.

#### Clone tree branch lengths

To depict the relative evolutionary distance between subclones, branch lengths were scaled based on the number of additional CNVs acquired by each descendant clone. Specifically, we applied a logarithmic transformation to the CNV difference using the formula: bk = 10×log₂(|Z_descendant| - |Z_parent|), where bk was the branch length (in pixels), and Z represented the number of CNV events in the descendant and parent clones. An arbitrary scaling factor was included to ensure branches remained visually discernible, even when CNV differences were minimal.

#### Clone tree node sizes

The size of each node (circle) representing a clone was proportional to the number of epithelial spots assigned to that clone, relative to the total epithelial spots in the sample. Clone diameters were computed using the formula: dl = √p, where dl is the diameter of the clone (in pixels), and p is the percentage of epithelial spots assigned to the clone.

### Lineage inference in ST-seq data using monocle

To investigate potential functional transitions and lineage differentiation among epithelial spots in ST-seq data, we employed the Monocle2 package (v.2.28.0). Epithelial clusters were directly input into Monocle2 for trajectory analysis. Specifically, we used the distinct GeneTest function to perform density peak clustering (via the Monocle dpFeature algorithm), allowing for the identification of differentially expressed genes among clusters. Default Monocle2 parameters were applied for dimensionality reduction and cell ordering, enabling the inference of epithelial spot differentiation trajectories. Endothelial cells trajectories were inferred using Monocle2 under the default settings. Integrated gene expression matrices exported from Seurat were used to construct a CellDataSet in Monocle2. All variable genes identified by the differential GeneTest function were used for cell ordering via the set Ordering Filter function. Dimensionality reduction was performed without additional normalization using the DDRTree method in the reduce Dimension step.

### Survival analysis of CNV-associated signatures

Survival associations of CNV-associated gene signatures were assessed using TCGA-BRCA cohort. The maximum significant cut-off point of each gene was calculated based on the best p-value. Kaplan-Meier survival curves were generated to evaluate the association of these gene signatures with overall patient survival outcomes.

### Statistical analyses

All data were analyzed and visualized by R (v.4.3.1) in this study. Kaplan-Meier survival curves were generated with K-M Plotter tool (http://www.kmplot.com). The hazard ratio (HR) and the 95% confidence interval (CI) were computed by using the univariate Cox proportional hazards regression analysis. Wilcox test was used to assess the difference between groups in this study. *P* < 0.05 were considered statistically significant in all statistical tests. Visualization was done using the ggplot2 (v.3.4.3) R package.

## Results

### A high-resolution cellular landscape of HER2 amplified breast cancer

**Fig. 1:**
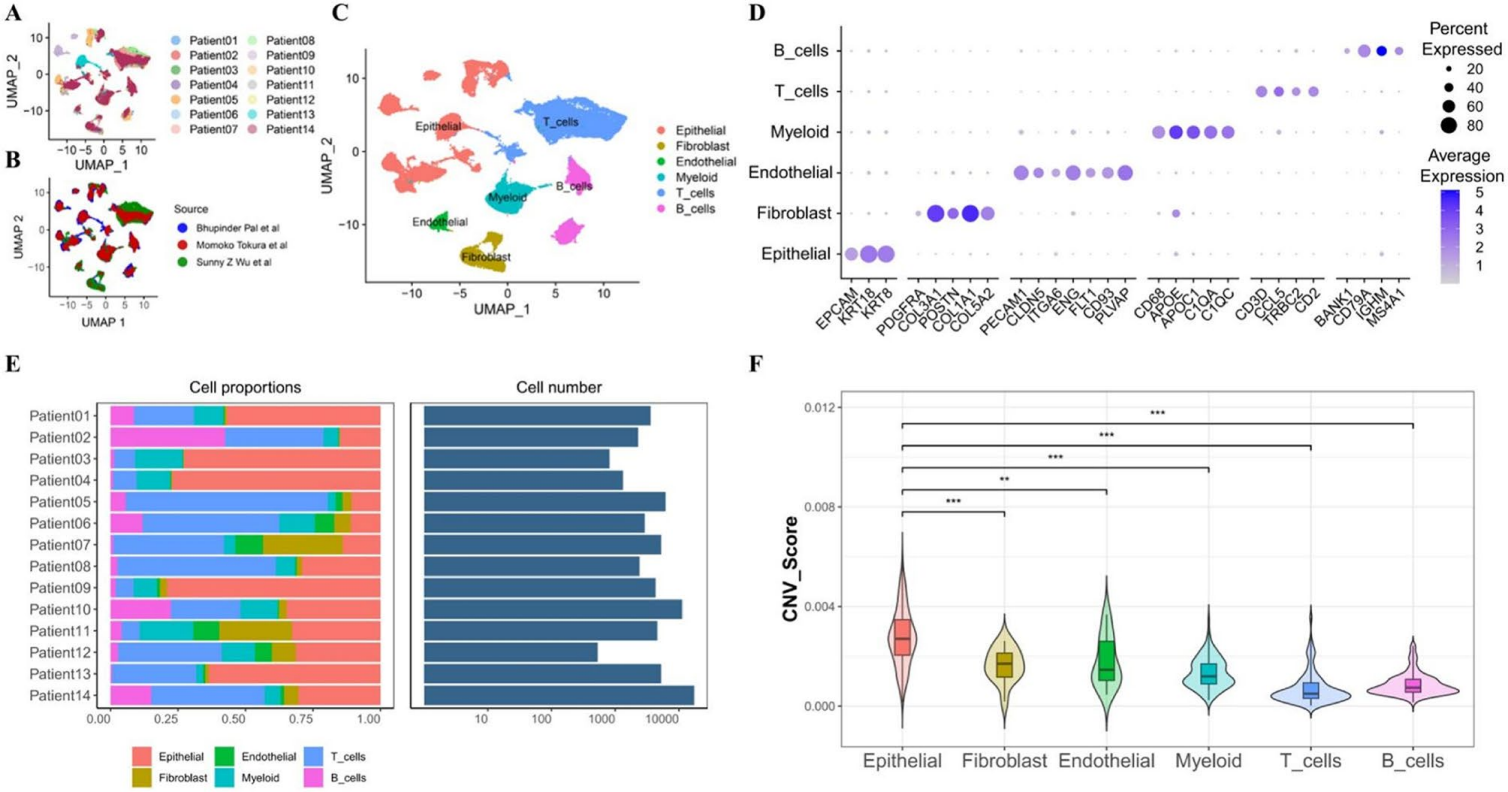
Single-cell RNA sequencing depicted the high-resolution cellular landscape in HER2 amplified primary breast cancer. **A-C**. Uniform manifold approximation and projection (UMAP) plots were colored by patients(**A**) or sources of the samples (**B**) from 14 HER2 amplified breast cancer patients. **C.** Six cell clusters were annotated, including epithelial cells, fibroblasts, endothelial cells, myeloid cells, T cells and B cells. **D.** Cell clusters were mainly annotated by canonical marker genes, including EPCAM (epithelial cells), PDGFR (fibroblasts), PECAM1 (endothelial cells), CD68 (myeloid cells), CD3D (T cells) and MS4A1 (B cells). **E.** Bar plots showed the proportion of each cell type and the total cell number in each patient. **F.** Violin plot showed the CNV score of different cell types in HER2 amplified breast cancer.Epithelial cells had significantly higher score thanother cells types, analyzed by wilcox.test, ***p-value < 0.001, **p-value < 0.01.

To explore the cell type and cellular characteristics of HER2 + breast cancer, we analyzed scRNA profiling data of 14 HER2 + primary breast cancer patients from three research studies^4,5,12^(Fig. 1A and B), including 4 DCIS patients and 10 IDC patients (Supplementary Table 1).Finally, 68,064 high-quality cells were obtained for further analysis (Fig. 1C) and annotated into six main cell types based on canonical lineage marker genes (Fig. 1D), which contained EPCAM for epithelial cells, PDGFR for fibroblasts, PECAM1 for endothelial cells, CD68 for myeloid cells, CD3D for T cellsand MS4A1 for B cells^16^(Supplementary Fig. 1). The number of cells per sample was ranged from 800 to 12,000 (Fig. 1E).

To further explore the genomic variation of these cells, we performed inferCNV analysis. We assessed CNV levels in every cell and each cell cluster^13^. Interestingly, the CNV score of epithelial cells (0.0019) was significantly higher than the other cell types (Fig. 1F). This finding provides important clues to our understanding of the genomic variant characteristics of epithelial cells in HER2 + breast cancer.

### The characteristics and the evolution of epithelial cells in HER2 amplified breast cancer

**Fig. 2:**
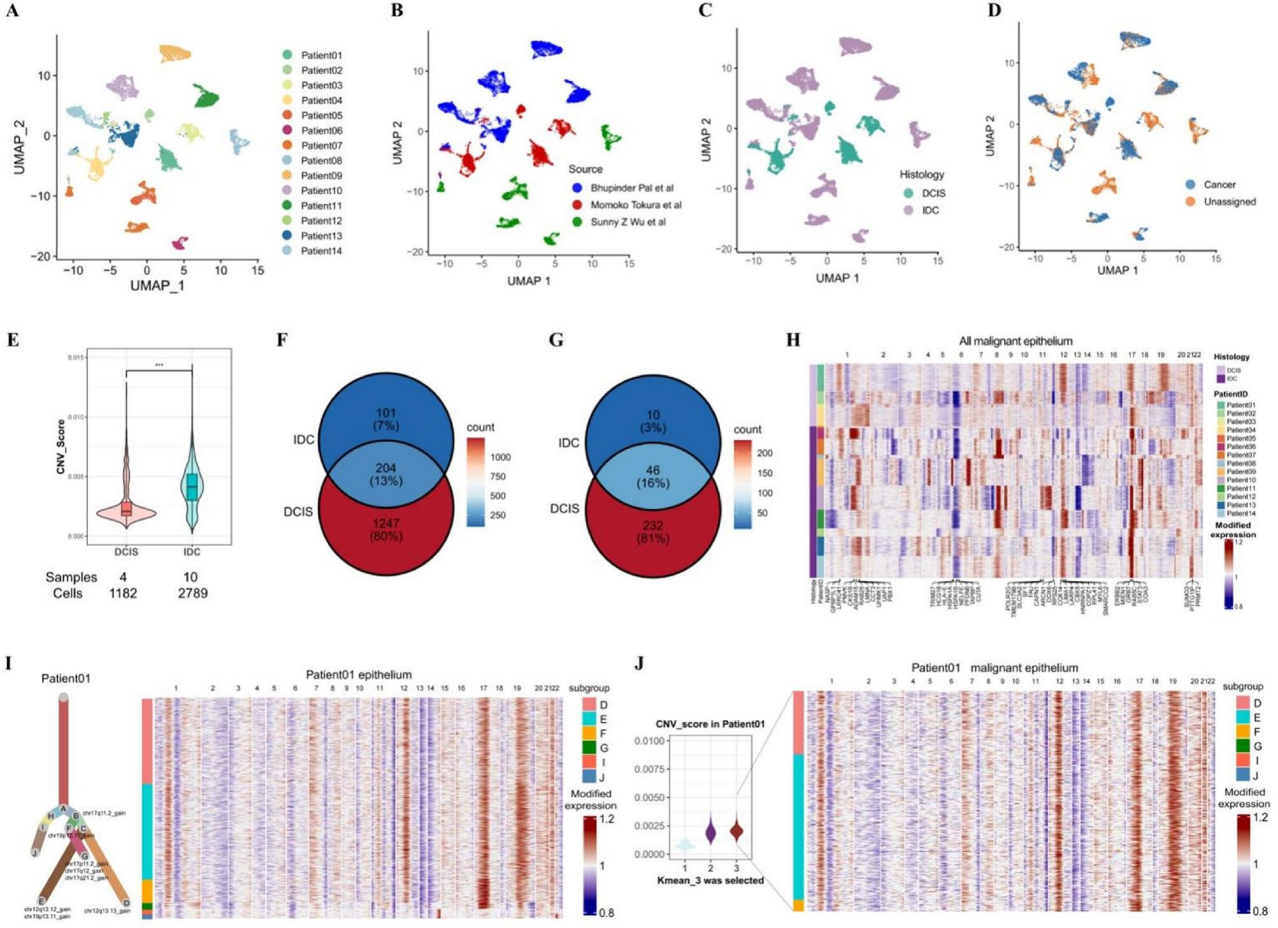
The landscape of copy number variations in epithelial cells of HER2-amplified breast cancer. **A-C**. Uniform manifold approximation and projection (UMAP) visualization of epithelial cells from 14 HER2 + patients, colored by: (A) patient origin, (B) sample source, and (C) tissue type. **D.** UMAP visualization of all epithelial cells were classified into tumor (steel blue) and unassigned (persimmon Orange). **E.** Violin plot showed the CNV score in DCIS and IDC in HER2 + breast cancer (wilcox.test, *** *p* < 0.05). **F.** Venn diagram showing CNV-altered genes shared between DCIS and IDC samples. **G.** Venn diagram depicting chromosomal regions with CNV alterations shared between DCIS and IDC samples. **H.** Consolidated heatmap of malignant cells from all 14 patients, with common representative genes labeled below. **I.** Tumor phylogenetic tree based on CNV patterns (left). Corresponding heatmap (right) displayed CNV content, patterns and clonal structures across cell subsets. Columns represented chromosomal regions, and rows showed CNV scores per cell (red amplification, blue deletion). **J.** Based on the K-means algorithm, the epithelial cells were divided into three subpopulations according to the CNV scores (left). The subset with the highest mean CNV score was selected as the putative malignant cell subset and its heatmap was displayed (right).

A total of 11,320 epithelial cells were annotated from 14 patients diagnosed with DCIS or IDC, sourced from three research studies (Fig. 2A, B and C).Since DNA copy number variations constitute a major oncogenic driver in breast cancer, cells of the same lineage would share similar CNV signatures^11,17^.CNV profiles of individual epithelial cells were inferred by the inferCNV tool^14^, to characterize the clonal CNV patterns and genomic heterogeneity.

Therefore, we presented a comprehensive picture of the presumed malignant cell profile from all 14 patients. The CNV burden exhibited no significant correlation with the number of epithelial cells selected per sample or with tumor subtype (Supplementary Fig. 2A–2D). Epithelial cells from each patient were classified into three populations by the CNV scores (Supplementary Fig. 2E). Malignant cell selection was performed by prioritizing cells exhibiting the highest CNV scores within each sample, with all other cells designated as unassigned group^15^. Then 3,971 tumor cells and 6,384 unassigned epithelial cells were successfully identified. UMAP plot showed the distribution of all malignant epithelial cells in a multidimensional space (Fig. 2D). Our comprehensive analysis confirmed significantly higher CNV burden in IDC versus DCIS (0.0042 vs. 0.0027, *p* < 0.005; Fig. 2E), indicating enhanced genomic instability during invasive progression.

Through inferred CNVs across all malignant epithelial cell clusters, we observed extensive overlap in CNV profiles between DCIS and IDC samples, uncovering both shared breast cancer-associated changes and patient-specific alterations. 204 genes within 46 genomic regions were consistently altered across all 14 patients in our study cohort (Fig. 2F and G). We identified amplifications in chromosome 17 regions (q12, q21.1, q21.2, q25.3), and in chr1 (q21.3, q22, q23.1, q23.2, q23.3, q24.1, p34.1, p34.2), 7(q13), chr11 (q12.1, q12.2, q12.3, q13.1), chr12 (q13.2, q13.3, q13.12, q13.13), chr20 (p11.23, p11.22, p11.21, q11.21, q11.22, p12.1) and chr21 (q22.2, q22.3, q22.11, q22.12, q22.13)^18,19^. Deletions were found in chromosomes 6 (p22.1, p21.33, p21.32, p21.31, p22.3, p22.2) and chr11 (q21, q22.3, q23.1, q23.2, q23.3, q24.1, q24.2, q24.3). Through rigorous screening of regions exhibiting high-level amplifications and deep deletions, we finally identified 147 key genes distributed across 16 genomic loci. For each of these regions, we highlighted three representative genes in heatmap visualization (Fig. 2H).

Specifically, our analysis revealed high-level amplification of the 17q12 chromosomal region containing ERBB2 (HER2), the canonical driver of HER2-amplified breast cancer (Fig. 2H, Supplementary Fig. 3A and 3B). Heatmap visualization demonstrated concordance between ERBB2 amplification status and expression patterns, showing the robust correlation between ERBB2 expression and RNA-inferred CNV level. Quantitative analysis showed a significant positive correlation between HER2 expression and CNV scores (13/14 patients; *r* = 0.1–0.7, *p* < 0.05) (Supplementary Fig. 4). Patient 08 (*r* = − 0.06) may reflect unique biological or technical confounding factors. This finding is in line with previous DNA-sequencing studies^20,21^, indicating that HER2 amplification cooperates with other genomic alterations to drive breast cancer progression.

Based on the genetic profiles amplification and deletion in Patient01, we reconstructed a CNV-based phylogenetic tree of tumor evolution, which delineated distinct clonal architectures, CNV patterns, and subpopulation-specific genomic alterations across cell lineages (Fig. 2I). This single-cell resolution analysis allowed us to resolve the subclonal diversity that is often obscured in bulk DNA or RNA sequencing data. Clonal evolution and CNV heatmaps in other patients further support our findings and provide us with a more comprehensive view of the evolution of epithelial cells in HER2 + breast cancer (Supplementary Fig. 5). The heatmap of the predicted malignant cellsin the Patient01 sample showed obviously stronger CNV pattern (Fig. 2J, right).

Notably, Patient02 presented an unusual case with a large tumor size (73 mm) and low ER/PR expression despite being pathologically diagnosed as DCIS, exhibiting a CNV score (0.0052) that actually exceeded the IDC group average (0.0042) (Supplementary Fig. 6).

### Spatial transcriptomemapped the CNV spectrum of HER2 + breast cancer

**Fig. 3:**
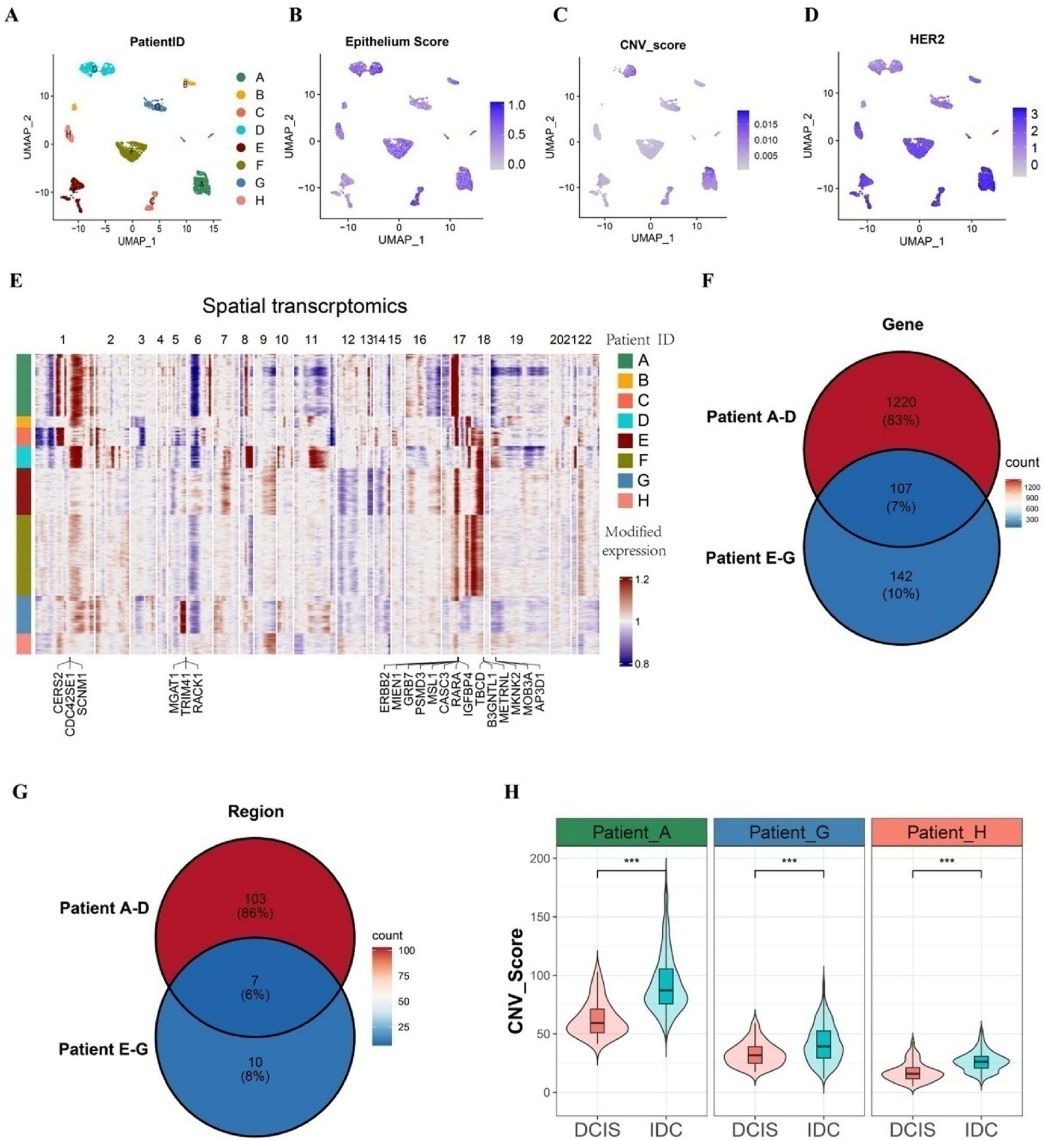
Spatial transcriptome mapped the cope number variation (CNV) spectrum in HER2amplified breast cancer. **A-D**. Uniform manifold approximation and projection (UMAP) visualization of malignant epithelial cells from 8 HER2 + patients, colored by: (A) patient ID, (B) epithelium score, (C) CNV score and (D) HER2 expression. **E**. Heatmap of malignant cells from 8 patients displayed CNV content, with common representative genes labeled below. Columns represented patient ID and chromosomal regions, and rows showed CNV scores per cell (red amplification, blue deletion). **F**. Venn diagram showing CNV-altered genes shared across 8 samples. **G.** Venn diagram depicting chromosomal regions with CNV alterations shared among 8 samples. **H.** Violin plot showed the CNV score of DCIS spots and IDC spots in three HER2 + breast cancer patients (wilcox.test, *** *p* < 0.05).

To validate our approach for identifying malignant epithelial cells, we performed comprehensive CNV profiling analysis of 36 spatial transcriptomic sections from 8 patients, encompassing over 1,200 spatial regions^4^. Based on manually annotated histological sections, all malignant epithelial regions were identified and validated using canonical epithelial cell markers derived from single-cell RNA sequencing. UMAP displayed all the malignant epithelial cells colored by the eight patients, epithelium score, CNV score or HER2 expression (Fig. 3A and B 3C and 3D).

We performed correlation analyses between HER2 expression and CNV scores across all malignant epithelial cells from the 8 patients (Supplementary Fig. 7A). In all cases, HER2 expression levels showed a positive correlation with CNV scores (r 0.1–0.5, *p* < 0.05). Consistently, correlation analyses across malignant epithelial cells from all eight patients demonstrated a consistent positive association between epithelial scores and CNV scores (r 0.05–0.35, *p* < 0.05, Supplementary Fig. 7B).

Widespread large-scale CNVs were detected within the tumor regions, revealing both patient-specific copy number alterations and common CNV patterns associated with breast cancer, such as amplifications in chr1q, chr5q, chr17q and chr19p^18^(Fig. 3E). Further analysis identified a set of 107 genes spanning 7 regions that were consistently altered across all 8 patients (Fig. 3F and G). The heatmap was annotated with hotspot genes and corresponding genomic regions (Fig. 3H). These common genes were annotated on the CNV heatmap, with a prominent amplification in 17q12, ERBB2, further validating the accuracy of CNV inference. Besides HER2 amplified, other genomic alterations on chromosome 17q were also identified, including 17q21.1, 17q21.2 and 17q25.3 (Fig. 3E).

Both DCIS and IDC lesions were coexisted in the Patient A, G and H (Supplementary Fig. 8). Substantial CNV events in both DCIS and IDC spots were observed, with IDC spots exhibiting a remarkably higher burden of CNVs compared to DCIS ones (Fig. 3H). This finding is consistent with the results previously obtained from scRNA seq data (Fig. 2J). These findings indicate distinct CNV profiles among DCIS and IDC clones, although the potential influence of extrinsic factors within the tumor ecosystem warrants further investigation.

### Spatial transcriptome revealed tumor heterogeneity and evolutionary pathways

To specifically characterize the molecular alterations associated with HER2 amplification during the DCIS-to-IDC transition, we conducted detailed multi-region analysis of three representative cases containing both DCIS and IDC pathological components: six consecutive cryosections from Patient A, three sections from Patient G, and three sections from Patient H, enabling systematic comparison of paired pre-invasive and invasive lesions within individual tumor ecosystems.

**Fig. 4:**
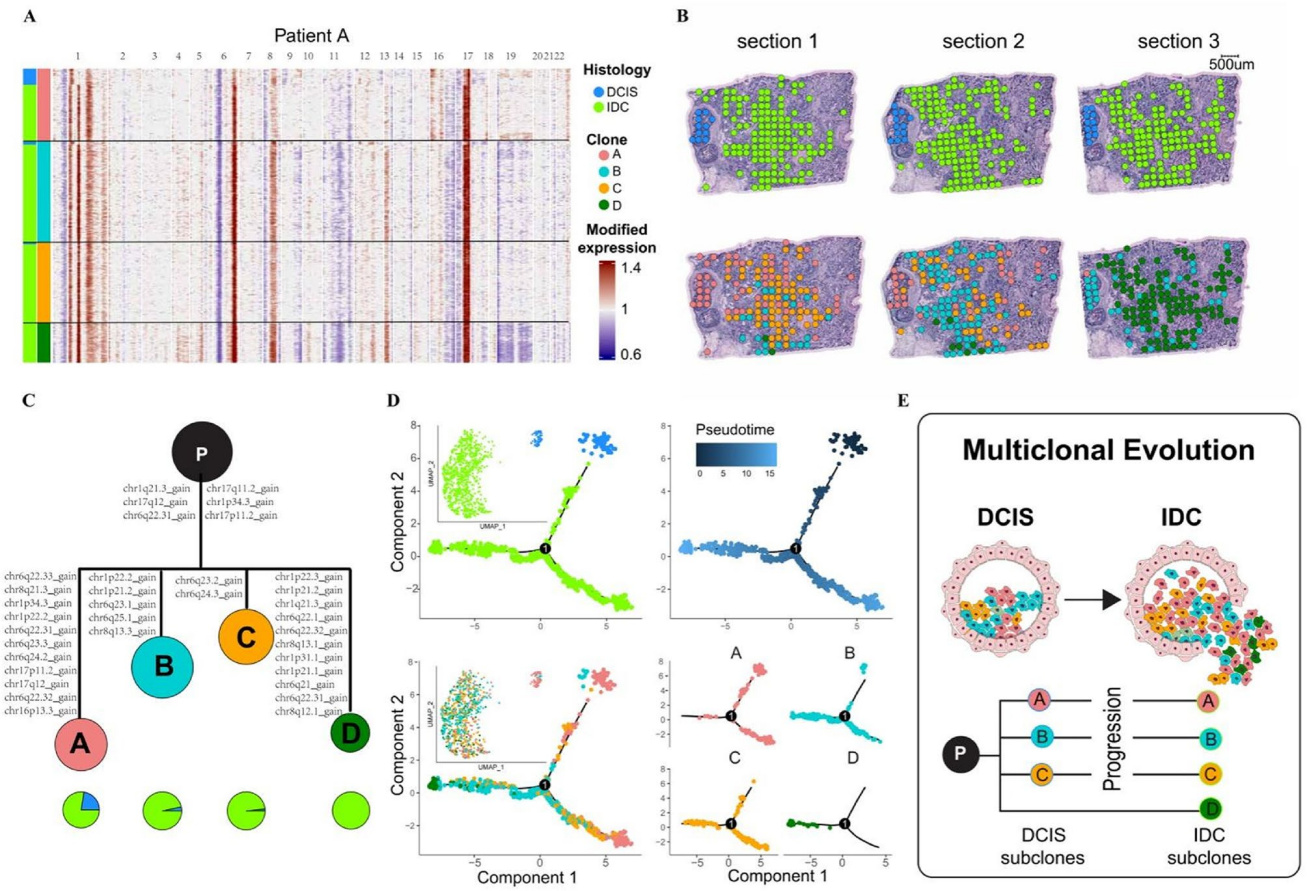
Spatial transcriptome revealed tumor heterogeneity and subclonal evolution in Patient A. **A**. Heatmap displayed copy number variation (CNV) contents and patterns in Patient A. Columns represented chromosomal regions, histology types (DCIS: dodger blue, IDC: chartreuse) and clones (clone A: light coral, clone B: dark turquoise, clone C: orange, and clone D: green). Rows showed CNV scores per cell (red amplification, blue deletion). Color scheme maintained consistent throughout Fig. 4. **B.** The epithelial histological characteristics (top panel) and tumor sub-clones (bottom panel) were mapped across all spatial spots in Patient A, corresponding tumor lesion sections. **C.** The phylogenetic cloning tree showed the tumor clonal evolution in Patient A, with clone area proportional to spot count and branch lengths reflecting weighted CNV alterations (log-scale), complemented by a DCIS/IDC composition pie chart (dodger blue/chartreuse). The black clone P was inferred common ancestors. **D.** Pseudotemporal analysis via Monocle tracks pathological progression (DCIS→IDC) and subclonal dynamics along evolutionary trajectories. **E.** Two distinct progression models emerge in Patient A: the multiclonal invasion model (migration of clones A-C) and independent evolution model (emergence of clone D), both originating from a common progenitor population.

All spatial transcriptomic spots were annotated based on pathological diagnosis and unique gene expression profiles. Both DCIS and IDC spots were precisely mapped onto pathological tissue sections (Fig. 4A). CNV quantification and tumor subcloning analysis was performed using siCNV methodology (Supplementary Table 2), with results spatially mapped onto each tissue section (Fig. 4A and B). The heatmap analysis confirmed HER2 overexpression patterns aligned with pathological findings (Fig. 4A). We found a shared ancestral clone containing fundamental CNV alterations in Patient A: chr1q21.3 amplification, chr1p34.3 amplification, chr6q22.31 amplification, chr17q11.2 amplification, chr17q12 (HER2) amplification, and chr17p11.2 amplification (Fig. 4A and C).

Subsequent subclones developed distinct CNV profiles. These molecular events were distributed across both DCIS and IDC histological regions and among different tumor subclones (Fig. 4C and E). This analysis demonstrates how spatial transcriptomics combined with CNV profiling can reveal both the phylogenetic relationships and histological distribution of tumor subclones within individual patients.

Spatial analysis of discriminatory events through siCNV mapping elucidated potential tumor clonal evolution. Complementary application of pseudo-temporal analysis (Monocle) enabled further characterization of pathological progression and subclonal dynamics. Notably, DCIS clones were spersed in start of the reconstructed evolutionary trajectory, with concurrent subclonal evolution of both DCIS and IDC populations evident in pseudo-temporal ordering (Fig. 4D). UMAP visualization confirmed the spatial distribution of distinct pathological subtypes and clonal populations (Fig. 4D). Collectively, integrated CNV and transcriptomic pseudo-temporal analysis enabled reconstruction of the putative evolutionary trajectory for DCIS in Patient A, demonstrating concordance between genomic and transcriptional regulation of tumor progression. Patient A exhibited two distinct tumor progression patterns: a multiclonal invasion model and an independent evolution model (Fig. 4E). In the multiclonal invasion scenario, three subclones (A, B, C) originating from a common normal progenitor cell (P1) collectively breached the ductal structure and co-migrated into surrounding tissues to form invasive carcinoma. Alternatively, the independent evolution model featured that clone D developing through a separate malignant transformation pathway from the same progenitor population (P1), demonstrating parallel clonal selection within the same tumor microenvironment.

**Fig. 5:**
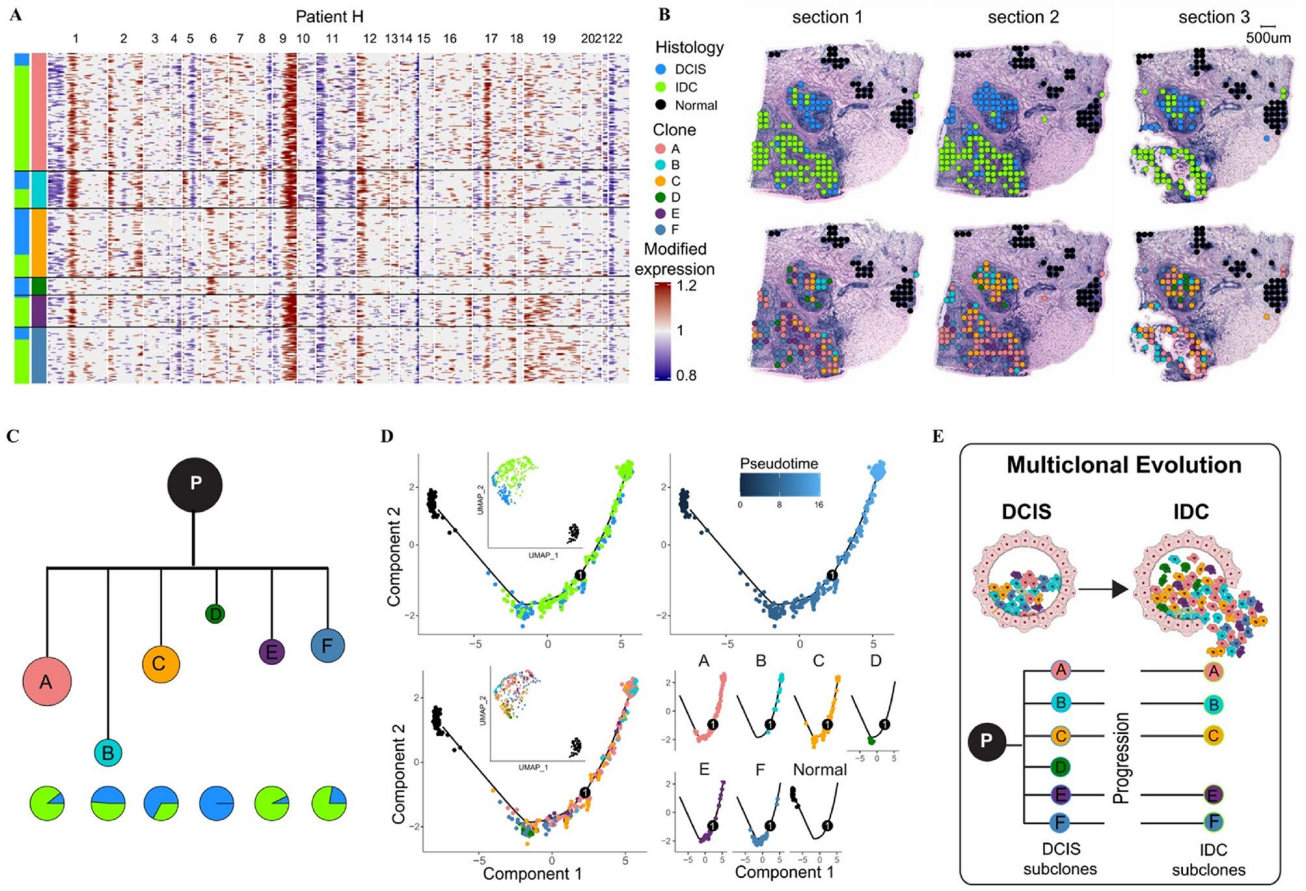
Spatial transcriptomic mapping of Patient H revealed tumor heterogeneity and subclonal progression patterns. **A.** Heatmap showing copy number variation (CNV) profiles across spatial transcriptomic spots in Patient H. Columns represent chromosomal regions, histological subtypes (DCIS: dodger blue; IDC: chartreuse), and identified clones (A: light coral; B: dark turquoise; C: orange; D: green; E: dark slate blue; F: steel blue). Rows correspond to individual cells, with CNV scores displayed as red (amplification) and blue (deletion). The color scheme is maintained consistently throughout the figure. **B.** Spatial mapping of epithelial histology (top panel) and tumor subclones (bottom panel) across tissue sections from Patient H, illustrating the distribution of DCIS and IDC components alongside subclonal architecture. **C.** Phylogenetic tree depicting tumor clonal evolution. Clone area is proportional to the number of associated spots, and branch lengths reflect the magnitude of CNV divergence (log scale). A pie chart illustrates the DCIS/IDC composition of each clone. The black node labeled “P” indicates the common progenitor. **D.** Pseudotemporal analysis using Monocle reconstructs the pathological progression from DCIS to IDC, tracking subclonal dynamics along the inferred trajectory. **E.** Two distinct evolutionary models were observed: (1) a multiclonal invasion model, in which clones A, B, C, E, and F—derived from a common progenitor (P1)—collectively invade adjacent tissues; and (2) an independent evolution model, in which clone D arises in situ from the same progenitor pool, highlighting parallel but divergent subclonal trajectories within a shared tumor ecosystem.

As with Patient A, the presumed directionality of progression from DCIS to IDC was inferred using both Monocle and CNV-based approaches. We next conducted a detailed analysis of Patient H (Fig. 5A and B), which included normal tissue, DCIS, and IDC spots. CNV quantification and tumor subclonal architecture were assessed using the siCNV methodology (Supplementary Table 3). Normal epithelial spots as controls, we analyzed copy number states across all spots and performed stratified clustering to classify them into six distinct clones (A-F) based on defined cluster separation thresholds (Fig. 5A and B). Spatial mapping revealed these computationally derived clonal clusters were organized into distinct groups that broadly correlated with histological subtypes (Fig. 5C and D). All analyzed spots contained both DCIS and IDC components, indicating their developmental relationship. These spatial-genomic patterns provided crucial insights into tumor subclone evolutionary trajectories (Fig. 5C). Importantly, every subclone contained both DCIS and IDC components, demonstrating their close biological relationship. Notably, DCIS clones were interspersed among IDC clones in the reconstructed evolutionary trajectory, with concurrent subclonal evolution of both DCIS and IDC populations evident in pseudo-temporal ordering (Fig. 5D). Patient H demonstrated dual tumor progression pathways: (1) a multiclonal invasion pattern where five genetically distinct subclones (A, B, C, E, F) derived from a common normal progenitor (P1) cooperatively invaded through ductal barriers into adjacent tissues, and (2) an independent evolution pattern where clone D arose in situ from the same progenitor pool (P1), illustrating concurrent but divergent clonal selection mechanisms within the same tumor ecosystem (Fig. 5E).

Patient G exhibited two distinct evolutionary patterns: (1) an evolutionary bottleneck model characterized by selective migration of clone C (with clones A and B remaining non-migratory), and (2) an independent evolution model featuring the emergence of clone D, both originating from a common progenitor population (Supplementary Fig. 9). These observations across all three patients collectively support a multi-threaded evolutionary model of DCIS to IDC progression.

### CNV loci genes predicted the survival of HER2 + breast cancer

CNV frequencies between the TCGA-BRCA dataset and our scRNA-seq data revealed highly concordant patterns of gene amplification and deletion (Supplementary Fig. 10A). We next sought to address a key clinical question: whether these hotspot CNV genes hold prognostic relevance. To investigate this, we collected clinical outcome data and gene expression profiles from cancer patients in publicly available data. Breast cancer patients with HER2 + were stratified into groups based on high or low expression of genes associated with chromosomal instability regions, and survival analyses were performed.

**Fig. 6:**
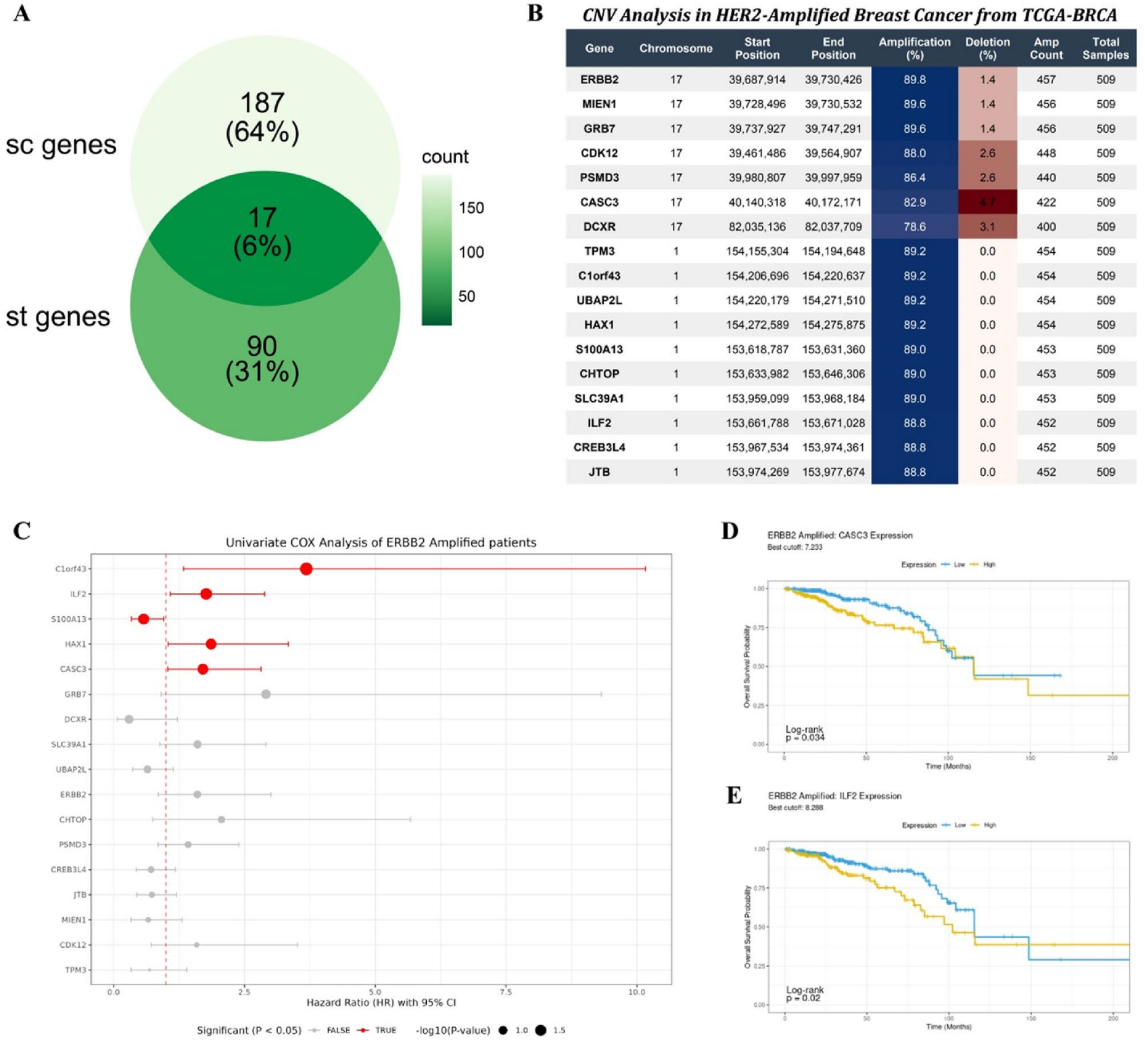
Copy number variation-associated genes predicted survival outcomes in HER2-amplified breast cancer. **A.** Venn diagram showing copy number variation associated genes shared between 14 single-cell RNA sequencing samples and 8 spatial transcriptomics samples. **B**. CNV analysis in HER2‑amplified breast cancer from the TCGA‑BRCA cohort. **C**. Univariate Cox analysis of the 14 CNV‑associated genes in ERBB2‑amplified patients. **D.** Kaplan-Meier plot illustrating the impact of CASC3 expression on overall survival in HER2-amplified breast cancer patients. **E.** Kaplan-Meier Plot showed survival impact of ILF2 stratification in HER2 amplified breast cancer patients.

Further integrative analysis of CNV-associated genes revealed 204 shared genes across 14 scRNA-seq samples and 107 common genes across 8 spatial transcriptomics samples, with 17 genes overlapping between both datasets (Fig. 6A). These shared genes include CDK12, ERBB2, MIEN1, GRB7, PSMD3, CASC3, S100A13, CHTOP, ILF2, SLC39A1, CREB3L4, JTB, TPM3, C1orf43, UBAP2L, HAX1, and DCXR. These genes are located within four genomic loci, that chr17q12, chr17q21.1, chr1q21.3, and chr17q25.3, and exhibited high-level amplification^22^. Consistent high-level amplification of these genes was also observed in HER2-amplified patients from the TCGA-BRCA cohort (Fig. 6B).

Univariate Cox regression analyses were performed to evaluate the impact of each genomic alteration signature on patient survival (Fig. 6C). Five genes showed a significant association between expression level and overall survival (*p* < 0.05). Notably, CASC3 and ILF2 showed high-level amplifications^23,24^. CASC3, in the chr17q21.1 region, demonstrated a strong association between its expression level and survival outcomes in HER2-positive breast cancer. Higher expression of CASC3 was significantly associated with shorter overall survival (OS) (Fig. 6D). And ILF2 over-expression also showed a significant correlation with worse OS (Fig. 6E). High expression of C1orf43 and HAX1 also correlated with adverse outcomes (Supplementary Figs. 10B and 10 C), whereas elevated S100A13 expression was associated with better prognosis compared with low-expression counterparts (Supplementary Fig. 10D).

Univariate and multivariate Cox models adjusting for key clinical covariates (age, race, and tumor stage) were subsequently applied to each of the five prognostic genes(Supplementary Tables 4–8). Advanced age was consistently associated with worse prognosis across nearly all gene subgroups (except in the HAX1 high-expression group in univariate analysis, *p* = 0.6). Patients identified as Black or African American exhibited significantly poorer survival outcomes in multivariate models across all five genes. Furthermore, advanced tumor stage III/IV was significantly associated with worse prognosis in several analyses, including HAX1 (univariate), the ILF2 low-expression group (univariate), CASC3 (univariate), the S100A13 high-expression group (univariate), and the C1orf43 high-expression group (univariate). These findings suggest that specific CNV signatures may serve as clinically relevant predictors of survival in HER2-positive breast cancer.

## Discussion

The degree of genomic instability was assessed based on the number of CNVs, the proportion of the genome affected by deletion of heterozygosity (LOH), and the presence or absence of whole-genome duplication^2^. These criteria reflect the genomic heterogeneity driven by whole-genome alterations, chromothripsis, and aneuploidy, which are hallmarks of human cancer. In this study, we observed that one DCIS patient had a large tumor size (73 mm). This finding raises the important question^6,8^: whether spatial tumor heterogeneity and tumor size were correlated with CNV accumulation.

In order to answer this question, we performed a comprehensive CNV analysis of HER2-positive breast cancer, generating a high resolution landscape of CNV atlas comprising 68,064 single cells from 14 patients and 4,764 spatial transcriptomic spots from 8 patients. For each individual cell or spatial spot, we inferred CNVs from transcriptomic data, thereby enabling high-resolution characterization of intratumoral heterogeneity. This work extends the utility of scRNA-seq and ST-seq to map both the CNV and transcriptional landscapes of tumor cells. Our single‑cell and spatial transcriptomic analysis overcomes the limitations of bulk sequencing. Unlike bulk approaches that average signals across cell populations, our method resolves intratumoral heterogeneity and subclonal architecture at cellular resolution. It further identifies rare malignant or transitional cell states that are typically undetectable in bulk assays.

Notably, significant CNV events were already observed in DCIS^25^, suggesting that considerable genomic alterations emerge early during tumorigenesis. Moreover, we also observed widespread shared CNV regions between DCIS and IDC, suggesting that many genomic aberrations were established at early stages and maintained throughout tumor progression^11,26^. These findings challenge the hypothesis that DCIS and IDC arise from independently evolving clonal populations. Instead, our data support a clonal continuity model, in which DCIS and IDC share common genomic origins and succession progression pathways.

Notably, it is important to note that HER2 expression status is not uniform across all DCIS lesions or during disease progression. Not all DCIS lesions are HER2-positive, and discordant HER2 status between paired DCIS and IDC components is well-documented^27,28^. Specifically, HER2-positive DCIS can coexist with HER2-negative IDC, and conversely, HER2-negative DCIS can progress to HER2-positive invasive carcinoma. This heterogeneity underscores the complexity of HER2 signaling in breast cancer evolution. Our observations of shared CNV alterations, including HER2 amplification events between DCIS and IDC components within individual patients, should be interpreted within this broader context. Therefore, HER2-negative DCIS may indeed give rise to HER2-positive invasive disease. Our findings highlight the substantial intrinsic heterogeneity within HER2-positive breast tumors. Evolutionary tree reconstruction and CNV pattern analysis revealed the presence of distinct tumor subclones in patients with both DCIS and IDC lesions^4^. These subclones exhibited unique spatial distributions and distinct CNV profiles, which underlie the heterogeneity of therapeutic response, and clinical outcomes among patients with HER2-positive disease. Furthermore, the proportions of different clones varied across samples and regions, and the relationships among clones, competitive or cooperative, remain an open question warranting further investigation.

Previous studies have suggested that CNV accumulation is a gradual process that may begin prior to overt tumor formation and subsequently escalate during tumor progression through successive cycles of proliferation and differentiation^9,29^. While DCIS and IDC share core somatic mutations, invasive lesions acquire further alterations and exhibit increased chromosomal instability (Yates et al., 2017; Casasent et al., 2018). Thus, our findings reinforce the concept that the transition to invasion involves clonal expansion from a common origin coupled with progressive genomic accumulation, reflected in the heightened CNV burden of IDC. While DCIS and IDC are traditionally distinguished based on the histological presence or absence of ductal wall invasion, molecular-level distinctions between them have remained unclear. In this study, we demonstrate, across multiple patients and within individual patients, that DCIS consistently harbors fewer CNV events compared to matched IDC samples. The greater burden of CNVs in IDC than in DCIS indicated that IDC exhibits higher levels of genomic instability and is more prone to acquiring additional genomic alterations. This may be attributable to the more aggressive proliferative nature of IDC cells and their compromised DNA replication and repair mechanisms.

Due to the complexity and plasticity of tumor evolution, we also identified instances of CNV reversion, amplifications followed by deletions, or deletions followed by subsequent amplifications, underscoring the non-linear nature of CNV evolution. Our findings demonstrate that DCIS progression in HER2-positive breast cancer follows multiple coexisting evolutionary theories^19,30^, including independent clonal branching, bottleneck evolution, and multiclonal invasion. This study identifies the multi-threaded evolutionary model as characteristic features of HER2-positive DCIS progression. Future studies leveraging larger cohorts and integrative multi-omic analyses will be essential to fully elucidate the mechanisms underlying HER2-positive breast cancer progression.

The greater CNV burden observed in IDC may reflect its higher malignancy and worse prognosis.^31^ These additional CNV regions may offer opportunities to develop gene-level tools to discriminate between DCIS and IDC, a prospect that warrants further investigation. Overall, the higher number of CNV alterations in IDC is likely a consequence of its increased invasiveness and malignancy, and holds important implications for the diagnosis, therapeutic stratification, and prognostication of breast cancer^32^. Therefore, the CNV differences between DCIS and IDC in HER2-positive breast cancer highlight their underlying molecular heterogeneity. These genomic distinctions provide valuable insights into the biological divergence between in situ and invasive lesions, with potential translational relevance for more accurate tumor staging, prognostic evaluation, and the development of targeted therapeutic strategies.

One of the defining features of HER2-positive breast cancer is the presence of extensive CNVs^33^, which we systematically validated in this study. Beyond the well-established amplification of the ERBB2 locus, we observed other frequent CNVs across chromosome 17, particularly within the 17q12-21 region^22^. These amplifications, encompassing several cancer-related genes, were found to have a significant impact on patient prognosis^34^. Additional amplifications on chromosome 17 and alterations on other chromosomes were also closely associated with clinical outcomes in HER2-positive breast cancer.

These findings not only identify novel biomarkers for prognostic stratification but also provide a biological basis for refined molecular subtyping and staging^35^. Both DCIS and IDC often share CNV regions harboring key oncogenes such as HER2, CASC3^23,34^ and ILF2^24^, which play central roles in tumorigenesis and are targets for molecular therapies.

CASC3 were reported as both amplified and overexpressed in gastric cancer and hepatocellular carcinoma^23,34^. LINC00571/HNRNPK/ILF2/IDH2^36^ signaling axis has been shown to drive progression in triple-negative breast cancer and small cell lung cancer^37^. In concordance with prior studies, our analysis confirmed that elevated expression of either CASC3 or ILF2 correlates with significantly shorter OS.

However, distinct CNV regions differentiating DCIS from IDC were also identified, some of which include genes like CDH1^38^, CDKN2A^39^, and RB1^40^, implicated in cell adhesion, cell cycle regulation, and tumor suppression. These unique CNVs may serve as prognostic indicators or therapeutic targets specific to invasive transformation and metastatic potential^41^. Importantly, co-amplified genes may act synergistically to enhance the aggressiveness of HER2-positive breast cancer^42^. Thus, these loci represent potential biomarkers for risk stratification beyond HER2 status alone, offering a refined framework for predicting disease progression.

Comparison of gene expression patterns across tumor subclones suggests that CNV aberrations may underlie both disease progression and therapeutic resistance in HER2-positive breast cancer^22^. However, additional mechanisms, such as somatic mutations, epigenetic alterations, or regulatory changes, are also likely to modulate these effects^43,44^. Disentangling their contributions to cell-state transitions and phenotypic outcomes remains a considerable challenge. Accordingly, more advanced methods integrating genetic and epigenetic data are needed to fully elucidate the impact of genomic instability on tumor biology.

Our study provides a robust framework for investigating genomic integrity in HER2-positive breast cancer and enriches the molecular pathology toolkit for characterizing tumor heterogeneity. These insights lay the groundwork for improving early detection, tailoring local and systemic therapeutic strategies, and ultimately enhancing outcomes in patients with HER2-positive and other high-risk breast cancers. Taken together, our findings raise fundamental biological questions regarding tumor evolution, cooperative oncogenesis, and prognostic prediction in HER2-positive breast cancer, and offer new avenues for future research and clinical translation.

Despite the comprehensive analysis of CNVs in breast cancer presented in this study, several limitations remain. First, the analysis is based on a relatively small patient cohort, lacking large-scale validation across independent datasets. Second, the precise functional mechanisms by which CNVs contribute to tumor development and therapeutic response remain incompletely understood, necessitating further experimental and functional studies. Third, single-cell sequencing presents some extent analytical challenges, including technical noise, batch effects, limited cell numbers, and data interpretation complexity. Algorithmic refinements and robust computational pipelines are still needed to fully leverage the power of single-cell CNV analysis. Four, the clustering-based approach for identifying malignant cells prioritizes specificity to reliably enrich a high-confidence malignant population, albeit potentially at the expense of sensitivity. An additional technical limitation of this study lies in the integration of scRNA-seq data from three different sources using distinct preprocessing pipelines. Variability in ploidy estimation across these methods may influence the resulting CNV-derived signatures, potentially introducing bias into downstream analyses.

## Conclusion

Extensive shared CNVs between DCIS and IDC strongly suggest that these genomic aberrations occur early in tumorigenesis and are maintained throughout tumor evolution. Significant CNVs emerge in the DCIS stage indicates that genomic instability is already present at the initial phases of breast cancer development. IDC exhibits a higher burden of CNV events than DCIS, potentially reflecting an intensification of genomic instability as the disease progresses. Notably, the progression of DCIS to IDC aligns closely with a multi-linear evolutionary model, characterized by simultaneous alterations in multiple genes or chromosomal regions.

## Electronic Supplementary Material

Below is the link to the electronic supplementary material.

Supplementary Material 1

## Data Availability

All the public raw scRNA-seq datasets were available, from the Broad Institute Single Cell Portal (https://singlecell.broadinstitute.org/single_cell/study/SCP1039), NCBI GEO Series GSE161529, and GEO under accession numbers GSE195861 and GSE196208. The ST-seq dataset can be downloaded from zenodo (https://doi.org/10.5281/zenodo.4751624). The public code related to the analyses in this study can be found on GitHub at https://github.com/Swarbricklab-code/BrCa_cell_atlas. Any other additional information required to reanalyze the data reported in this work paper is available upon request, which should be addressed to lead contact Yuanqiang Duan (duanyuanqiang2008@163.com).
