# Supplementary material for: Decoding clone evolution in HER2 amplified breast cancer through single-cell and spatial transcriptomics analysis of copy number variations
Source: Sci Rep. 2026 Mar 16;16:13658. doi: 10.1038/s41598-026-44476-7 (PMC13125306; doi:10.1038/s41598-026-44476-7)
Supplement: Supplementary file 1 — Supplementary Material 1 [file 41598_2026_44476_MOESM1_ESM.docx]

**Title**: Decoding clone evolution in HER2 amplified breast cancer through single-cell and spatial transcriptomics analysis of copy number variations

**Running title**: CNV-driven evolution and spatial heterogeneity in HER2+ breast cancer

**Authors**: Jiao Yang^1,2,†^, Yong Li^3,†^, Suxia Luo^1^, Jian Wang^2^, Yuanqiang Duan^4,*^

**Affiliations**:

^1^Department of Medical Oncology, the Affiliated Cancer Hospital of Zhengzhou University & Henan Cancer Hospital, Zhengzhou 450008, China.

^2^Department of Oncology, the Second Affiliated Hospital of Zhengzhou University, Zhengzhou 450008, China

^3^Department of Breast Disease, Henan Breast Cancer Center, the Affiliated Cancer Hospital of Zhengzhou University & Henan Cancer Hospital, Zhengzhou, 450008, China.

^4^Department of Thoracic Surgery, the Affiliated Cancer Hospital of Zhengzhou University& Henan Cancer Hospital, Zhengzhou 450008, China.

^†^These authors contributed equally.

^*^Correspondence: [duanyuanqiang2008@163.com](mailto:duanyuanqiang2008@163.com)

Authors’ detail information:

**Jiao Yang**: 1.Department of Medical Oncology, the Affiliated Cancer Hospital of Zhengzhou University & Henan Cancer Hospital, Zhengzhou 450008, China; 2.Department of Oncology, the Second Affiliated Hospital of Zhengzhou University, Zhengzhou 450008, China, [yangjiao5620@163.com](mailto:yangjiao5620@163.com)

**Yong Li**: Department of Breast Disease, Henan Breast Cancer Center, the Affiliated Cancer Hospital of Zhengzhou University & Henan Cancer Hospital, Zhengzhou, 450008, China.,[zlyyliyong3737@zzu.edu.cn](mailto:zlyyliyong3737@zzu.edu.cn)

**Suxia Luo**: Department of Medical Oncology, the Affiliated Cancer Hospital of Zhengzhou University & Henan Cancer Hospital, Zhengzhou 450008, China, [luosxrm@163.com](mailto:luosxrm@163.com)

**Jian Wang:** Department of Oncology, the Second Affiliated Hospital of Zhengzhou University, Zhengzhou 450008, China, [wangjianfs@zzu.edu.cn](mailto:wangjianfs@zzu.edu.cn)

**Yuanqiang Duan**: Department of Thoracic Surgery, the Affiliated Cancer Hospital of Zhengzhou University & Henan Cancer Hospital, Zhengzhou 450008, Henan, China, [duanyuanqiang2008@163.com](mailto:duanyuanqiang2008@163.com)

**One Sentence Summary:** Single-cell and spatial transcriptomics revealed early genomic instability and cooperative copy number variation events that drive clonal evolution and heterogeneity in HER2-positive breast cancer progression from DCIS to IDC.

**Highlights:**

1. Early Emergence of in DCIS: significant copy number variations (CNVs) are already present at the DCIS stage, indicating early genomic instability in HER2+ breast cancer.
2. Clonal Evolution from DCIS to IDC: shared CNV regions between DCIS and IDC suggest a clonal evolutionary trajectory rather than independent lineage development.
3. Co-amplification Hotspots on Chr17: frequent co-amplification of HER2 and neighboring genes highlights potential synergistic drivers and therapeutic targets.
4. Spatial Clonal Diversity: spatial transcriptomics revealed spatially distinct tumor subclones with unique CNV profiles, mirroring the histological co-existence of DCIS and IDC
5. Clinical Implications of CNV Burden: CNV extent correlates with disease progression and patient prognosis, offering a framework for risk stratification and personalized therapy.

**Keywords:**

HER2-positive breast cancer; ductal carcinoma in situ; invasive ductal carcinoma; copy number variation; clonal evolution.

**Abstract**

Background: HER2-positive breast cancer exhibits marked genomic instability and heterogeneity, yet the clonal architecture and copy number variation (CNV) dynamics between ductal carcinoma in situ (DCIS) and invasive ductal carcinoma (IDC), remain poorly understood.

Methods: We analyzed single-cell RNA sequencing data from 14 HER2-positive breast cancer patients and spatial transcriptomics from 8 patients. CNVs were inferred to evaluate genomic alterations and reconstruct tumor subclone evolutionary trajectories. Survival analyses were performed on CNV-correlated transcripts.

Results: We identified 68,064 cells and 4,764 spatial transcriptomic spots, and observed early and pervasive CNV events in DCIS. IDC exhibiting a higher CNV burden supported the hypothesis of progressive genomic instability during tumor evolution. Shared CNV regions across DCIS and IDC suggested the common clonal origin, favoring a multi-threaded evolutionary model. Amplifications in chromosome 17q12-21 were associated with poor prognosis.

Conclusion: CNV-driven clonal evolution probably originates at early stages of HER2-positive breast cancer and persists through disease progression. Early CNV events may serve as predictive biomarkers and potential intervention targets to prevent disease advancement.

**Main Text:**

**INTRODUCTION**

Copy number variation (CNV), a form of genomic structural alteration involving segmental duplication or deletion of DNA, plays a significant role in the development and progression of breast cancer^1,2^. In breast cancer, CNVs have been closely linked to tumor initiation, heterogeneity, progression, and therapeutic response^3^. Notably, CNV landscapes in breast cancer are highly heterogeneous, varying not only between molecular subtypes but also between individual patients. In HER2-positive (HER2+) breast cancer, CNVs are reported to mainly contribute to tumor aggressiveness and metastasis^4^.

Ductal carcinoma in situ (DCIS) is considered a potential precursor to invasive ductal carcinoma (IDC). Since only an estimated 10-50% of untreated DCIS cases progress to IDC, overtreatment remains a significant concern^5-8^. Despite numerous efforts, the molecular mechanisms underlying DCIS-to-IDC progression remain poorly understood^6,9^. This underscores the urgent need for reliable prognostic biomarkers to distinguish indolent from high-risk lesions.

Recent advances in single-cell RNA sequencing (scRNA-seq) and spatial transcriptomics have enabled CNV inference at unprecedented resolution, offering insight into CNV patterns at both the cellular and spatial levels^1,10,11^. These technologies allow for the exploration of intratumoral heterogeneity, subclonal architecture, and evolutionary dynamics in situ. In this study, we applied these cutting-edge approaches to characterize CNV landscapes in HER2-positive DCIS and IDC breast cancer. Moreover, by comparing DCIS and IDC, we investigated the distinct CNV profiles and their implications for disease progression, therapeutic resistance, and prognosis in HER2-positive breast cancer.

**METHODS**

Data Sources

This study included public available data from four major sources^4,5,10,12^, comprising single-cell RNA sequencing (scRNA-seq) data from 14 HER2-positive breast cancer patients and spatial transcriptomics sequencing (ST-seq) data from 8 patients. The scRNA-seq datasets were obtained from the Broad Institute Single Cell Portal (SCP1039) (n = 4), GEO Series GSE161529 (n = 6), and NCBI GEO under accession numbers GSE195861 and GSE196208 (n = 4). All scRNA-seq data were generated using the 10x Genomics Chromium platform. The ST-seq dataset was achieved from zenodo (4751624) (n = 8). All cases were pathologically confirmed as HER2-enriched breast cancer based on immunohistochemistry (IHC) or fluorescence in situ hybridization (FISH), as reported in the original publications. Detailed clinical and pathological information for each patient was provided in Supplementary Table1.

Pre-processing and Quality Control of scRNA-seq Data

Filtered gene expression matrix for each sample were obtained from the sources described above and processed using R software (v4.3.1) with the Seurat package (v4.3.0.1). To ensure high data quality, a series of filtering steps were applied. For each sample, cells were excluded if they met any of the following criteria: fewer than 200 unique molecular identifiers (UMIs), fewer than 200 or more than 6,000 detected genes, or more than 20% of UMIs derived from mitochondrial transcripts-indicative of low-quality or dying cells. After quality control, 68,064 high-quality cells from 14 scRNA-seq samples were retained for downstream analysis. Similarly, after applying the same quality filtering criteria, 4764 spots from 8 ST-seq samples were included in subsequent analyses.

Integrative Analysis of Multiple Datasets

To perform dimensionality reduction, the 2,000 most variable genes were selected using the FindVariableFeatures function with the vst method in the Seurat package. These highly variable genes were then subjected to principal component analysis for linear dimensionality reduction. To correct for batch effects across different samples, the Harmony package (v0.1.1) was employed using the RunHarmony function. Subsequently, cell clustering was performed using Seurat’s FindNeighbors and FindClusters functions. The resolution parameter was adjusted to 0.1 to delineate clusters. Specifically, the top 30 principal components (PCs) were used to construct a shared nearest-neighbor (SNN) graph by calculating the neighborhood overlap between each cell and its nearest neighbors. Clusters were identified using a modularity optimization-based clustering algorithm applied to the SNN graph. For visualization, uniform manifold approximation and projection (UMAP) was conducted on the top 30 PCs using the RunUMAP function, enabling two-dimensional representation of the cellular landscape and cluster distribution.

Cell clusters annotation

Cell clusters were annotated based on the expression of canonical marker genes corresponding to known cell types. Specifically, the fibroblast cells were identified by expression of PDGFRA, COL3A1, POSTN, COL1A1 and COL5A2; endothelial cells by PECAM1, CLDN5, ITGA6, ENG, FLT1, CD93, PLVAP; myeloid cells by CD68, APOE, APOC1, C1QA and C1QC; B cells by BANK1, CD79A, IGHM, and MS4A1; T cells by CD3D, CLL5, TRBC2, CD2.

Epithelial cells were further refined using SingleR-based (v.2.2.0) annotation. Following epithelial cell selection, standard preprocessing steps, including normalization, dimensionality reduction, and clustering, were repeated. Due to the biological heterogeneity inherent to tumors, batch correction (via Harmony) was not applied in this step. Dimensionality reduction was visualized using the RunUMAP function, and epithelial marker expression was used to validate the epithelial cell clusters.

InferCNV analysis and phylogenetic trees construction

CNV signals at the single-cell level were estimated using the inferCNV^13^ (v1.16.0) with cutoff value 0.1, and a dynamic threshold of 1.5 standard deviations was applied for signal denoising^14^. Required input files, including raw count matrices, cell annotations, and gene / chromosome position data, were prepared following official guidelines (https://github.com/broadinstitute/inferCNV). Immune and endothelial cells, particularly T cells, were used as the primary reference population for CNV normalization. To assess potential reference bias, normal epithelial cells from adjacent non-tumor tissues were also utilized. Comparable CNV patterns were observed with both reference populations, demonstrating the robustness of the inferred CNV profiles. For each sample, gene expression values of individual cells were re-standardized and scaled to a range between -1 and 1. A CNV score for each cell was calculated as the sum of all CNV regions. Gene-level CNV inferences were derived using a Hidden Markov Model (HMM)-based approach, specifically from the output file HMM_CNV_predictions.HMMi6.rand_trees.hmm_mode-subclusters.Pnorm_0.5.pred_cnv_regions.dat. These inferred CNVs were then visualized using UPhyloplot2 to construct phylogenetic trees, thereby illustrating clonal architecture and evolutionary trajectories of the tumor.

Identification of tumor cells in scRNA-seq data

A clustering approach analogous to the K-means algorithm was employed to classify epithelial cells in order to distinguish tumor cells from unassigned cells^15^. The cluster number (k=3) was optimized to balance cluster distinctness, population size, and biological interpretability. For each sample, epithelial cells were grouped into three main clusters based on their CNV scores. The cluster with the highest average CNV score was inferred to contain tumor cells. The remaining clusters were designated as unassigned. This strategy was designed to selectively identify tumor cells with high confidence and preserve the inherent heterogeneity of tumor populations.

Venn diagram analysis

CNV-altered genes and regions were extracted from single-cell RNA-seq and spatial transcriptomics datasets for each patient sample. With the help of the ggVennDiagram package, shared and unique CNV-altered genes and regions across samples were identified and represented in Venn diagrams to highlight overlaps and distinctions in CNV profiles among different samples.

Spatial transcriptomics data analysis

Raw spatial transcriptomics data and code were obtained as described in the original publication^4^and repository (https://github.com/almaan/her2st).

Definition of epithelial spots signature in ST-seq

Epithelial spot signatures were quantified using the AddModuleScore function in the Seurat package, based on the expression of canonical epithelial markers: EPCAM, KRT8, and KRT18.

Manual algorithmic tree construction from inferCNV outputs

Clone tree consensus spatial inferred CNV (siCNV) event calling

Putative subclonal CNV events were identified by integrating both the HMM-based outputs (from files infercnv.17_HMM_predHMMi6.hmm_mode-samples.png and 17_HMM_predHMMi6.hmm_mode-samples.pred_cnv_regions.dat), along with manual curation of CNV profiles. A final consensus CNV event list was generated for each clone and used to construct clonal phylogenies. Clone trees were built by identifying CNV events shared across cell clusters, based on the biological assumption that CNV events were irreversible. Thus, shared CNVs suggest common ancestry among the clusters. This framework allowed for the hierarchical reconstruction of subclonal lineages.

Clone tree branch lengths

To depict the relative evolutionary distance between subclones, branch lengths were scaled based on the number of additional CNVs acquired by each descendant clone. Specifically, we applied a logarithmic transformation to the CNV difference using the formula: bk = 10×log₂(|Z_descendant| - |Z_parent|), where bk was the branch length (in pixels), and Z represented the number of CNV events in the descendant and parent clones. An arbitrary scaling factor was included to ensure branches remained visually discernible, even when CNV differences were minimal.

Clone tree node sizes

The size of each node (circle) representing a clone was proportional to the number of epithelial spots assigned to that clone, relative to the total epithelial spots in the sample. Clone diameters were computed using the formula: dl = √p, where dl is the diameter of the clone (in pixels), and p is the percentage of epithelial spots assigned to the clone.

Lineage inference in ST-seq data using monocle

To investigate potential functional transitions and lineage differentiation among epithelial spots in ST-seq data, we employed the Monocle2 package (v.2.28.0). Epithelial clusters were directly input into Monocle2 for trajectory analysis. Specifically, we used the distinct GeneTest function to perform density peak clustering (via the Monocle dpFeature algorithm), allowing for the identification of differentially expressed genes among clusters. Default Monocle2 parameters were applied for dimensionality reduction and cell ordering, enabling the inference of epithelial spot differentiation trajectories. Endothelial cells trajectories were inferred using Monocle2 under the default settings. Integrated gene expression matrices exported from Seurat were used to construct a CellDataSet in Monocle2. All variable genes identified by the differential GeneTest function were used for cell ordering via the set Ordering Filter function. Dimensionality reduction was performed without additional normalization using the DDRTree method in the reduce Dimension step.

Survival analysis of CNV-associated signatures

Survival associations of CNV-associated gene signatures were assessed using TCGA-BRCA cohort. The maximum significant cut-off point of each gene was calculated based on the best p-value. Kaplan-Meier survival curves were generated to evaluate the association of these gene signatures with overall patient survival outcomes.

Statistical analyses

All data were analyzed and visualized by R (v.4.3.1) in this study. Kaplan-Meier survival curves were generated with K-M Plotter tool (http://www.kmplot.com). The hazard ratio (HR) and the 95% confidence interval (CI) were computed by using the univariate Cox proportional hazards regression analysis. Wilcox test was used to assess the difference between groups in this study. P< 0.05 were considered statistically significant in all statistical tests. Visualization was done using the ggplot2 (v.3.4.3) R package.

**RESULTS**

**A high-resolution cellular landscape of HER2 amplified breast cancer**

To explore the cell type and cellular characteristics of HER2+ breast cancer, we analyzed scRNA profiling data of 14 HER2+ primary breast cancer patients from three research studies^4,5,12^ (Figure 1A and 1B), including 4 DCIS patients and 10 IDC patients (Supplementary Table 1).Finally, 68,064 high-quality cells were obtained for further analysis (Figure 1C) and annotated into six main cell types based on canonical lineage marker genes (Figure 1D), which contained EPCAM for epithelial cells, PDGFR for fibroblasts, PECAM1 for endothelial cells, CD68 for myeloid cells,CD3D for T cellsand MS4A1 for B cells^16^ (Supplementary Figure 1). The number of cells per sample was ranged from 800 to 12,000 (Figure 1E).

To further explore the genomic variation of these cells, we performed inferCNV analysis. We assessed CNV levels in every cell and each cell cluster^13^. Interestingly, the CNV score of epithelial cells (0.0019) was significantly higher than the other cell types (Figure 1F). This finding provides important clues to our understanding of the genomic variant characteristics of epithelial cells in HER2+ breast cancer.

**The characteristics and the evolution of epithelial cells in HER2 amplified breast cancer.**

A total of 11,320 epithelial cells were annotated from 14 patients diagnosed with DCIS or IDC, sourced from three research studies (Figure 2A, 2B and 2C).Since DNA copy number variations constitute a major oncogenic driver in breast cancer, cells of the same lineage would share similar CNV signatures^11,17^.CNV profiles of individual epithelial cells were inferred by the inferCNV tool^14^, to characterize the clonal CNV patterns and genomic heterogeneity.

Therefore, we presented a comprehensive picture of the presumed malignant cell profile from all 14 patients. The CNV burden exhibited no significant correlation with the number of epithelial cells selected per sample or with tumor subtype (Supplementary Figure 2A–2D). Epithelial cells from each patient were classified into three populations by the CNV scores (Supplementary Figure 2E). Malignant cell selection was performed by prioritizing cells exhibiting the highest CNV scores within each sample, with all other cells designated as unassigned group^15^. Then 3,971 tumor cells and 6,384 unassigned epithelial cells were successfully identified. UMAP plot showed the distribution of all malignant epithelial cells in a multidimensional space (Figure 2D). Our comprehensive analysis confirmed significantly higher CNV burden in IDC versus DCIS (0.0042 vs 0.0027, p<0.005; Figure 2E), indicating enhanced genomic instability during invasive progression.

Through inferred CNVs across all malignant epithelial cell clusters, we observed extensive overlap in CNV profiles between DCIS and IDC samples, uncovering both shared breast cancer-associated changes and patient-specific alterations. 204 genes within 46 genomic regions were consistently altered across all 14 patients in our study cohort (Figure 2F and 2G). We identified amplifications in chromosome 17 regions (q12, q21.1, q21.2, q25.3), and in chr1 (q21.3, q22, q23.1, q23.2, q23.3, q24.1, p34.1, p34.2), 7(q13), chr11 (q12.1, q12.2, q12.3, q13.1), chr12 (q13.2, q13.3, q13.12, q13.13), chr20 (p11.23, p11.22, p11.21, q11.21, q11.22, p12.1) and chr21 (q22.2, q22.3, q22.11, q22.12, q22.13)^18,19^. Deletions were found in chromosomes 6 (p22.1, p21.33, p21.32, p21.31, p22.3, p22.2) and chr11 (q21, q22.3, q23.1, q23.2, q23.3, q24.1, q24.2, q24.3). Through rigorous screening of regions exhibiting high-level amplifications and deep deletions, we finally identified 147 key genes distributed across 16 genomic loci. For each of these regions, we highlighted three representative genes in heatmap visualization (Figure 2H).

Specifically, our analysis revealed high-level amplification of the 17q12 chromosomal region containing ERBB2 (HER2), the canonical driver of HER2-amplified breast cancer (Figure 2H, Supplementary Figure 3A and 3B). Heatmap visualization demonstrated concordance between ERBB2 amplification status and expression patterns, showing the robust correlation between ERBB2 expression and RNA-inferred CNV level. Quantitative analysis showed a significant positive correlation between HER2 expression and CNV scores (13/14 patients; r=0.1-0.7, p<0.05) (Supplementary figure 4). Patient 08 (r = −0.06) may reflect unique biological or technical confounding factors. This finding is in line with previous DNA-sequencing studies^20,21^, indicating that HER2 amplification cooperates with other genomic alterations to drive breast cancer progression.

Based on the genetic profiles amplification and deletion in Patient01, we reconstructed a CNV-based phylogenetic tree of tumor evolution, which delineated distinct clonal architectures, CNV patterns, and subpopulation-specific genomic alterations across cell lineages (Figure 2I). This single-cell resolution analysis allowed us to resolve the subclonal diversity that is often obscured in bulk DNA or RNA sequencing data. Clonal evolution and CNV heatmaps in other patients further support our findings and provide us with a more comprehensive view of the evolution of epithelial cells in HER2+ breast cancer (Supplementary figure 5). The heatmap of the predicted malignant cellsin the Patient01 sample showed obviously stronger CNV pattern (Figure 2J, right).

Notably, Patient02 presented an unusual case with a large tumor size (73 mm) and low ER/PR expression despite being pathologically diagnosed as DCIS, exhibiting a CNV score (0.0052) that actually exceeded the IDC group average (0.0042) (Supplementary figure 6).

**Spatial transcriptomemapped the CNV spectrum of HER2+ breast cancer**

To validate our approach for identifying malignant epithelial cells, we performed comprehensive CNV profiling analysis of 36 spatial transcriptomic sections from 8 patients, encompassing over 1,200 spatial regions^4^. Based on manually annotated histological sections, all malignant epithelial regions were identified and validated using canonical epithelial cell markers derived from single-cell RNA sequencing. UMAP displayed all the malignant epithelial cells colored by the eight patients, epithelium score, CNV score or HER2 expression (Figure 3A, 3B 3C and 3D).

We performed correlation analyses between HER2 expression and CNV scores across all malignant epithelial cells from the 8 patients (Supplementary figure 7A). In all cases, HER2 expression levels showed a positive correlation with CNV scores (r 0.1-0.5, p< 0.05). Consistently, correlation analyses across malignant epithelial cells from all eight patients demonstrated a consistent positive association between epithelial scores and CNV scores (r 0.05-0.35, p< 0.05, Supplementary figure 7B).

Widespread large-scale CNVs were detected within the tumor regions, revealing both patient-specific copy number alterations and common CNV patterns associated with breast cancer, such as amplifications in chr1q, chr5q, chr17q and chr19p^18^ (Figure 3E). Further analysis identified a set of 107 genes spanning 7 regions that were consistently altered across all 8 patients (Figure 3F &3G). The heatmap was annotated with hotspot genes and corresponding genomic regions (Figure 3H). These common genes were annotated on the CNV heatmap, with a prominent amplification in 17q12, ERBB2, further validating the accuracy of CNV inference. Besides HER2 amplified, other genomic alterations on chromosome 17q were also identified, including 17q21.1, 17q21.2 and 17q25.3 (Figure 3E).

Both DCIS and IDC lesions were coexisted in the Patient A, G and H (Supplementary Figure 8). Substantial CNV events in both DCIS and IDC spots were observed, with IDC spots exhibiting a remarkably higher burden of CNVs compared to DCIS ones (Figure 3H). This finding is consistent with the results previously obtained from scRNA seq data (Figure 2J). These findings indicate distinct CNV profiles among DCIS and IDC clones, although the potential influence of extrinsic factors within the tumor ecosystem warrants further investigation.

**Spatial transcriptome revealed tumor heterogeneity and evolutionary pathways**

To specifically characterize the molecular alterations associated with HER2 amplification during the DCIS-to-IDC transition, we conducted detailed multi-region analysis of three representative cases containing both DCIS and IDC pathological components: six consecutive cryosections from Patient A, three sections from Patient G, and three sections from Patient H, enabling systematic comparison of paired pre-invasive and invasive lesions within individual tumor ecosystems.

All spatial transcriptomic spots were annotated based on pathological diagnosis and unique gene expression profiles. Both DCIS and IDC spots were precisely mapped onto pathological tissue sections (Figure 4A). CNV quantification and tumor subcloning analysis was performed using siCNV methodology (Supplementary Table2), with results spatially mapped onto each tissue section (Figures 4A, 4B). The heatmap analysis confirmed HER2 overexpression patterns aligned with pathological findings (Figure 4A). We found a shared ancestral clone containing fundamental CNV alterations in Patient A: chr1q21.3 amplification, chr1p34.3 amplification, chr6q22.31 amplification, chr17q11.2 amplification, chr17q12 (HER2) amplification, and chr17p11.2 amplification (Figure 4A and 4C).

Subsequent subclones developed distinct CNV profiles. These molecular events were distributed across both DCIS and IDC histological regions and among different tumor subclones (Figure 4C and 4E). This analysis demonstrates how spatial transcriptomics combined with CNV profiling can reveal both the phylogenetic relationships and histological distribution of tumor subclones within individual patients.

Spatial analysis of discriminatory events through siCNV mapping elucidated potential tumor clonal evolution. Complementary application of pseudo-temporal analysis (Monocle) enabled further characterization of pathological progression and subclonal dynamics. Notably, DCIS clones were spersed in start of the reconstructed evolutionary trajectory, with concurrent subclonal evolution of both DCIS and IDC populations evident in pseudo-temporal ordering (Figure 4D). UMAP visualization confirmed the spatial distribution of distinct pathological subtypes and clonal populations (Figure 4D). Collectively, integrated CNV and transcriptomic pseudo-temporal analysis enabled reconstruction of the putative evolutionary trajectory for DCIS in Patient A, demonstrating concordance between genomic and transcriptional regulation of tumor progression. Patient A exhibited two distinct tumor progression patterns: a multiclonal invasion model and an independent evolution model (Figure 4E). In the multiclonal invasion scenario, three subclones (A, B, C) originating from a common normal progenitor cell (P1) collectively breached the ductal structure and co-migrated into surrounding tissues to form invasive carcinoma. Alternatively, the independent evolution model featured that clone D developing through a separate malignant transformation pathway from the same progenitor population (P1), demonstrating parallel clonal selection within the same tumor microenvironment.

As with Patient A, the presumed directionality of progression from DCIS to IDC was inferred using both Monocle and CNV-based approaches. We next conducted a detailed analysis of Patient H (Figures 5A and 5B), which included normal tissue, DCIS, and IDC spots. CNV quantification and tumor subclonal architecture were assessed using the siCNV methodology (Supplementary Table 3). Normal epithelial spots as controls, we analyzed copy number states across all spots and performed stratified clustering to classify them into six distinct clones (A-F) based on defined cluster separation thresholds (Figure 5A and 5B). Spatial mapping revealed these computationally derived clonal clusters were organized into distinct groups that broadly correlated with histological subtypes (Figures 5C and 5D). All analyzed spots contained both DCIS and IDC components, indicating their developmental relationship. These spatial-genomic patterns provided crucial insights into tumor subclone evolutionary trajectories (Figure 5C). Importantly, every subclone contained both DCIS and IDC components, demonstrating their close biological relationship. Notably, DCIS clones were interspersed among IDC clones in the reconstructed evolutionary trajectory, with concurrent subclonal evolution of both DCIS and IDC populations evident in pseudo-temporal ordering (Figure 5D). Patient H demonstrated dual tumor progression pathways: (1) a multiclonal invasion pattern where five genetically distinct subclones (A, B, C, E, F) derived from a common normal progenitor (P1) cooperatively invaded through ductal barriers into adjacent tissues, and (2) an independent evolution pattern where clone D arose in situ from the same progenitor pool (P1), illustrating concurrent but divergent clonal selection mechanisms within the same tumor ecosystem (Figure 5E).

Patient G exhibited two distinct evolutionary patterns: (1) an evolutionary bottleneck model characterized by selective migration of clone C (with clones A and B remaining non-migratory), and (2) an independent evolution model featuring the emergence of clone D, both originating from a common progenitor population (Supplementary Figure 9). These observations across all three patients collectively support a multi-threaded evolutionary model of DCIS to IDC progression.

**CNV loci genes predicted the survival of HER2+ breast cancer**

CNV frequencies between the TCGA-BRCA dataset and our scRNA-seq data revealed highly concordant patterns of gene amplification and deletion (Supplementary Figure 10A). We next sought to address a key clinical question: whether these hotspot CNV genes hold prognostic relevance. To investigate this, we collected clinical outcome data and gene expression profiles from cancer patients in publicly available data. Breast cancer patients with HER2+ were stratified into groups based on high or low expression of genes associated with chromosomal instability regions, and survival analyses were performed.

Further integrative analysis of CNV-associated genes revealed 204 shared genes across 14 scRNA-seq samples and 107 common genes across 8 spatial transcriptomics samples, with 17 genes overlapping between both datasets (Figure 6A). These shared genes include CDK12, ERBB2, MIEN1, GRB7, PSMD3, CASC3, S100A13, CHTOP, ILF2, SLC39A1, CREB3L4, JTB, TPM3, C1orf43, UBAP2L, HAX1, and DCXR. These genes are located within four genomic loci, that chr17q12, chr17q21.1, chr1q21.3, and chr17q25.3, and exhibited high-level amplification^22^. Consistent high-level amplification of these genes was also observed in HER2-amplified patients from the TCGA-BRCA cohort (Figure 6B).

Univariate Cox regression analyses were performed to evaluate the impact of each genomic alteration signature on patient survival (Figure 6C). Five genes showed a significant association between expression level and overall survival (p< 0.05). Notably, CASC3 and ILF2 showed high-level amplifications^23,24^. CASC3, in the chr17q21.1 region, demonstrated a strong association between its expression level and survival outcomes in HER2-positive breast cancer. Higher expression of CASC3 was significantly associated with shorter overall survival (OS) (Figure 6D). And ILF2 over-expression also showed a significant correlation with worse OS (Figure 6E). High expression of C1orf43 and HAX1 also correlated with adverse outcomes (Supplementary Figures 10B and 10C), whereas elevated S100A13 expression was associated with better prognosis compared with low-expression counterparts (Supplementary Figure 10D).

Univariate and multivariate Cox models adjusting for key clinical covariates (age, race, and tumor stage) were subsequently applied to each of the five prognostic genes(Supplementary Table 4-8). Advanced age was consistently associated with worse prognosis across nearly all gene subgroups (except in the HAX1 high-expression group in univariate analysis, p = 0.6). Patients identified as Black or African American exhibited significantly poorer survival outcomes in multivariate models across all five genes. Furthermore, advanced tumor stage III/IV was significantly associated with worse prognosis in several analyses, including HAX1 (univariate), the ILF2 low-expression group (univariate), CASC3 (univariate), the S100A13 high-expression group (univariate), and the C1orf43 high-expression group (univariate). These findings suggest that specific CNV signatures may serve as clinically relevant predictors of survival in HER2-positive breast cancer.

**DISCUSSION**

The degree of genomic instability was assessed based on the number of CNVs, the proportion of the genome affected by deletion of heterozygosity (LOH), and the presence or absence of whole-genome duplication^2^. These criteria reflect the genomic heterogeneity driven by whole-genome alterations, chromothripsis, and aneuploidy, which are hallmarks of human cancer. In this study, we observed that one DCIS patient had a large tumor size (73 mm). This finding raises the important question^6,8^: whether spatial tumor heterogeneity and tumor size were correlated with CNV accumulation.

In order to answer this question, we performed a comprehensive CNV analysis of HER2-positive breast cancer, generating a high resolution landscape of CNV atlas comprising 68,064 single cells from 14 patients and 4,764 spatial transcriptomic spots from 8 patients. For each individual cell or spatial spot, we inferred CNVs from transcriptomic data, thereby enabling high-resolution characterization of intratumoral heterogeneity. This work extends the utility of scRNA-seq and ST-seq to map both the CNV and transcriptional landscapes of tumor cells. Our single‑cell and spatial transcriptomic analysis overcomes the limitations of bulk sequencing. Unlike bulk approaches that average signals across cell populations, our method resolves intratumoral heterogeneity and subclonal architecture at cellular resolution. It further identifies rare malignant or transitional cell states that are typically undetectable in bulk assays.

Notably, significant CNV events were already observed in DCIS^25^, suggesting that considerable genomic alterations emerge early during tumorigenesis. Moreover, we also observed widespread shared CNV regions between DCIS and IDC, suggesting that many genomic aberrations were established at early stages and maintained throughout tumor progression^11,26^. These findings challenge the hypothesis that DCIS and IDC arise from independently evolving clonal populations. Instead, our data support a clonal continuity model, in which DCIS and IDC share common genomic origins and succession progression pathways.

Notably, it is important to note that HER2 expression status is not uniform across all DCIS lesions or during disease progression. Not all DCIS lesions are HER2-positive, and discordant HER2 status between paired DCIS and IDC components is well-documented^27,28^. Specifically, HER2-positive DCIS can coexist with HER2-negative IDC, and conversely, HER2-negative DCIS can progress to HER2-positive invasive carcinoma. This heterogeneity underscores the complexity of HER2 signaling in breast cancer evolution. Our observations of shared CNV alterations, including HER2 amplification events between DCIS and IDC components within individual patients, should be interpreted within this broader context. Therefore, HER2-negative DCIS may indeed give rise to HER2-positive invasive disease. Our findings highlight the substantial intrinsic heterogeneity within HER2-positive breast tumors. Evolutionary tree reconstruction and CNV pattern analysis revealed the presence of distinct tumor subclones in patients with both DCIS and IDC lesions^4^. These subclones exhibited unique spatial distributions and distinct CNV profiles, which underlie the heterogeneity of therapeutic response, and clinical outcomes among patients with HER2-positive disease. Furthermore, the proportions of different clones varied across samples and regions, and the relationships among clones, competitive or cooperative, remain an open question warranting further investigation.

Previous studies have suggested that CNV accumulation is a gradual process that may begin prior to overt tumor formation and subsequently escalate during tumor progression through successive cycles of proliferation and differentiation^9,29^. While DCIS and IDC share core somatic mutations, invasive lesions acquire further alterations and exhibit increased chromosomal instability (Yates et al., 2017; Casasent et al., 2018). Thus, our findings reinforce the concept that the transition to invasion involves clonal expansion from a common origin coupled with progressive genomic accumulation, reflected in the heightened CNV burden of IDC. While DCIS and IDC are traditionally distinguished based on the histological presence or absence of ductal wall invasion, molecular-level distinctions between them have remained unclear. In this study, we demonstrate, across multiple patients and within individual patients, that DCIS consistently harbors fewer CNV events compared to matched IDC samples. The greater burden of CNVs in IDC than in DCIS indicated that IDC exhibits higher levels of genomic instability and is more prone to acquiring additional genomic alterations. This may be attributable to the more aggressive proliferative nature of IDC cells and their compromised DNA replication and repair mechanisms.

Due to the complexity and plasticity of tumor evolution, we also identified instances of CNV reversion, amplifications followed by deletions, or deletions followed by subsequent amplifications, underscoring the non-linear nature of CNV evolution. Our findings demonstrate that DCIS progression in HER2-positive breast cancer follows multiple coexisting evolutionary theories^19,30^, including independent clonal branching, bottleneck evolution, and multiclonal invasion. This study identifies the multi-threaded evolutionary model as characteristic features of HER2-positive DCIS progression. Future studies leveraging larger cohorts and integrative multi-omic analyses will be essential to fully elucidate the mechanisms underlying HER2-positive breast cancer progression.

The greater CNV burden observed in IDC may reflect its higher malignancy and worse prognosis.^31^ These additional CNV regions may offer opportunities to develop gene-level tools to discriminate between DCIS and IDC, a prospect that warrants further investigation. Overall, the higher number of CNV alterations in IDC is likely a consequence of its increased invasiveness and malignancy, and holds important implications for the diagnosis, therapeutic stratification, and prognostication of breast cancer^32^. Therefore, the CNV differences between DCIS and IDC in HER2-positive breast cancer highlight their underlying molecular heterogeneity. These genomic distinctions provide valuable insights into the biological divergence between in situ and invasive lesions, with potential translational relevance for more accurate tumor staging, prognostic evaluation, and the development of targeted therapeutic strategies.

One of the defining features of HER2-positive breast cancer is the presence of extensive CNVs^33^, which we systematically validated in this study. Beyond the well-established amplification of the ERBB2 locus, we observed other frequent CNVs across chromosome 17, particularly within the 17q12-21 region^22^. These amplifications, encompassing several cancer-related genes, were found to have a significant impact on patient prognosis^34^. Additional amplifications on chromosome 17 and alterations on other chromosomes were also closely associated with clinical outcomes in HER2-positive breast cancer.

These findings not only identify novel biomarkers for prognostic stratification but also provide a biological basis for refined molecular subtyping and staging^35^. Both DCIS and IDC often share CNV regions harboring key oncogenes such as HER2, CASC3^23,34^ and ILF2^24^, which play central roles in tumorigenesis and are targets for molecular therapies.

CASC3 were reported as both amplified and overexpressed in gastric cancer and hepatocellular carcinoma^23,34^. LINC00571/HNRNPK/ILF2/IDH2^36^ signaling axis has been shown to drive progression in triple-negative breast cancer and small cell lung cancer^37^. In concordance with prior studies, our analysis confirmed that elevated expression of either CASC3 or ILF2 correlates with significantly shorter OS.

However, distinct CNV regions differentiating DCIS from IDC were also identified, some of which include genes like CDH1^38^, CDKN2A^39^, and RB1^40^, implicated in cell adhesion, cell cycle regulation, and tumor suppression. These unique CNVs may serve as prognostic indicators or therapeutic targets specific to invasive transformation and metastatic potential^41^. Importantly, co-amplified genes may act synergistically to enhance the aggressiveness of HER2-positive breast cancer^42^. Thus, these loci represent potential biomarkers for risk stratification beyond HER2 status alone, offering a refined framework for predicting disease progression.

Comparison of gene expression patterns across tumor subclones suggests that CNV aberrations may underlie both disease progression and therapeutic resistance in HER2-positive breast cancer^22^. However, additional mechanisms, such as somatic mutations, epigenetic alterations, or regulatory changes, are also likely to modulate these effects^43,44^. Disentangling their contributions to cell-state transitions and phenotypic outcomes remains a considerable challenge. Accordingly, more advanced methods integrating genetic and epigenetic data are needed to fully elucidate the impact of genomic instability on tumor biology.

Our study provides a robust framework for investigating genomic integrity in HER2-positive breast cancer and enriches the molecular pathology toolkit for characterizing tumor heterogeneity. These insights lay the groundwork for improving early detection, tailoring local and systemic therapeutic strategies, and ultimately enhancing outcomes in patients with HER2-positive and other high-risk breast cancers. Taken together, our findings raise fundamental biological questions regarding tumor evolution, cooperative oncogenesis, and prognostic prediction in HER2-positive breast cancer, and offer new avenues for future research and clinical translation.

Despite the comprehensive analysis of CNVs in breast cancer presented in this study, several limitations remain. First, the analysis is based on a relatively small patient cohort, lacking large-scale validation across independent datasets. Second, the precise functional mechanisms by which CNVs contribute to tumor development and therapeutic response remain incompletely understood, necessitating further experimental and functional studies. Third, single-cell sequencing presents some extent analytical challenges, including technical noise, batch effects, limited cell numbers, and data interpretation complexity. Algorithmic refinements and robust computational pipelines are still needed to fully leverage the power of single-cell CNV analysis. Four, the clustering-based approach for identifying malignant cells prioritizes specificity to reliably enrich a high-confidence malignant population, albeit potentially at the expense of sensitivity. An additional technical limitation of this study lies in the integration of scRNA-seq data from three different sources using distinct preprocessing pipelines. Variability in ploidy estimation across these methods may influence the resulting CNV-derived signatures, potentially introducing bias into downstream analyses.

Conclusion

Extensive shared CNVs between DCIS and IDC strongly suggest that these genomic aberrations occur early in tumorigenesis and are maintained throughout tumor evolution. Significant CNVs emerge in the DCIS stage indicates that genomic instability is already present at the initial phases of breast cancer development. IDC exhibits a higher burden of CNV events than DCIS, potentially reflecting an intensification of genomic instability as the disease progresses. Notably, the progression of DCIS to IDC aligns closely with a multi-linear evolutionary model, characterized by simultaneous alterations in multiple genes or chromosomal regions.

**Declarations:**

Data and code availability

All the public raw scRNA-seq datasets were available, from the Broad Institute Single Cell Portal (https://singlecell.broadinstitute.org/single_cell/study/SCP1039), NCBI GEO Series GSE161529, and GEO under accession numbers GSE195861 and GSE196208. The ST-seq dataset can be downloaded from zenodo (https://doi.org/10.5281/zenodo.4751624). The public code related to the analyses in this study can be found on GitHub at <https://github.com/Swarbricklab-code/BrCa_cell_atlas.> Any other additional information required to reanalyze the data reported in this work paper is available upon request, which should be addressed to lead contact Yuanqiang Duan ([duanyuanqiang2008@163.com](mailto:duanyuanqiang2008@163.com)).

Authors' contributions

Conceptualization: JY and YQD; Methodology& Data collection: JY and YQD; Investigation & Visualization: JY, YL and YQD; Project administration: JY, YL and YQD; Validation: SXL, YL, JW and YQD; Supervision: SXL and YQD; Writing & original draft: JY, YL and YQD; Writing, review & editing: JY, YL, SXL, JW and YQD.

Competing interest

The authors declare that the research was conducted in the absence of any commercial or financial relationships that could be construed as a potential conflict of interest. All the authors declare that they all consent for publication.

Acknowledgments

All authors appreciate the helps from the staff in the Affiliated Cancer Hospital of Zhengzhou University & Henan Cancer Hospital and the helps from the Second Affiliated Hospital of Zhengzhou University.

Funding

This work is supported by the National Natural Science Foundation of China (Youth Project) (Grant No. 82403484) to Yuanqiang Duan.

**REFERENCE**

1. Erickson A, He M, Berglund E, et al. Spatially resolved clonal copy number alterations in benign and malignant tissue. *Nature*. 2022;608(7922):360-367. doi:10.1038/s41586-022-05023-2

2. Steele CD, Abbasi A, Islam SMA, et al. Signatures of copy number alterations in human cancer. *Nature*. Jun 2022;606(7916):984-991. doi:10.1038/s41586-022-04738-6

3. Staaf J, Jönsson G, Ringnér M, et al. High-resolution genomic and expression analyses of copy number alterations in HER2-amplified breast cancer. *Breast Cancer Research*. 2010;12(3)doi:10.1186/bcr2568

4. Andersson A, Larsson L, Stenbeck L, et al. Spatial deconvolution of HER2-positive breast cancer delineates tumor-associated cell type interactions. *Nature Communications*. 2021;12(1)doi:10.1038/s41467-021-26271-2

5. Tokura M, Nakayama J, Prieto-Vila M, et al. Single-cell transcriptome profiling reveals intratumoral heterogeneity and molecular features of ductal carcinoma In situ. *Cancer Research*. 2022;82(18):3236-3248. doi:10.1158/0008-5472.Can-22-0090

6. Anstine LJ, Keri R. A new view of the mammary epithelial hierarchy and its implications for breast cancer initiation and metastasis. *J Cancer Metastasis Treat*. 2019;5doi:10.20517/2394-4722.2019.24

7. Abba MC, Gong T, Lu Y, et al. A Molecular Portrait of High-Grade Ductal Carcinoma In Situ. *Cancer Res*. Sep 15 2015;75(18):3980-90. doi:10.1158/0008-5472.CAN-15-0506

8. Pareja F, Brown DN, Lee JY, et al. Whole-Exome Sequencing Analysis of the Progression from Non-Low-Grade Ductal Carcinoma In Situ to Invasive Ductal Carcinoma. *Clin Cancer Res*. Jul 15 2020;26(14):3682-3693. doi:10.1158/1078-0432.CCR-19-2563

9. Nachmanson D, Officer A, Mori H, et al. The breast pre-cancer atlas illustrates the molecular and micro-environmental diversity of ductal carcinoma in situ. *NPJ Breast Cancer*. Jan 13 2022;8(1):6. doi:10.1038/s41523-021-00365-y

10. Wu SZ, Al-Eryani G, Roden DL, et al. A single-cell and spatially resolved atlas of human breast cancers. *Nature Genetics*. 2021;53(9):1334-1347. doi:10.1038/s41588-021-00911-1

11. Wang K, Kumar T, Wang J, et al. Archival single-cell genomics reveals persistent subclones during DCIS progression. *Cell*. 2023;186(18):3968-3982.e15. doi:10.1016/j.cell.2023.07.024

12. Pal B, Chen Y, Vaillant F, et al. A single‐cell RNA expression atlas of normal, preneoplastic and tumorigenic states in the human breast. *The EMBO Journal*. 2021;40(11)doi:10.15252/embj.2020107333

13. Patel AP, Tirosh I, Trombetta JJ, et al. Single-cell RNA-seq highlights intratumoral heterogeneity in primary glioblastoma. *Science*. Jun 20 2014;344(6190):1396-401. doi:10.1126/science.1254257

14. Xu K, Wang R, Xie H, et al. Single-cell RNA sequencing reveals cell heterogeneity and transcriptome profile of breast cancer lymph node metastasis. *Oncogenesis*. Oct 5 2021;10(10):66. doi:10.1038/s41389-021-00355-6

15. Itay Tirosh BI, 1,2,3*†‡ Sanjay M. Prakadan,1,4,5,6 Marc H. Wadsworth II,1,4,5,6 Daniel Treacy,1 John J. Trombetta,1 Asaf Rotem,1,2,3 Christopher Rodman,1 Christine Lian,7 George Murphy,7 Mohammad Fallahi-Sichani,8 Ken Dutton-Regester,1,2,9 Jia-Ren Lin,10 Ofir Cohen,1 Parin Shah,2 Diana Lu,1 Alex S. Genshaft,1,4,5,6 Travis K. Hughes,1,4,6,11 Carly G. K. Ziegler,1,4,6,11 Samuel W. Kazer,1,4,5,6 Aleth Gaillard,1,4,5,6 Kellie E. Kolb,1,4,5,6 Alexandra-Chloé Villani,1 Cory M. Johannessen,1 Aleksandr Y. Andreev,1 Eliezer M. Van Allen,1,2,3 Monica Bertagnolli,12,13 Peter K. Sorger,8,10,14 Ryan J. Sullivan,15 Keith T. Flaherty,15 Dennie T. Frederick,15 Judit Jané-Valbuena,1 Charles H. Yoon,12,13† Orit Rozenblatt-Rosen,1† Alex K. Shalek,1,4,5,6,11,16† Aviv Regev,1,17,18†‡ Levi A. Garraway. Dissecting the multicellular ecosystem of metastatic melanoma by single-cell RNA-seq. 2016;

16. Bhat-Nakshatri P, Gao H, Sheng L, et al. A single-cell atlas of the healthy breast tissues reveals clinically relevant clusters of breast epithelial cells. *Cell Rep Med*. Mar 16 2021;2(3):100219. doi:10.1016/j.xcrm.2021.100219

17. Cancer Genome Atlas N. Comprehensive molecular portraits of human breast tumours. *Nature*. Oct 4 2012;490(7418):61-70. doi:10.1038/nature11412

18. Vargas AC, McCart Reed AE, Waddell N, et al. Gene expression profiling of tumour epithelial and stromal compartments during breast cancer progression. *Breast Cancer Res Treat*. Aug 2012;135(1):153-65. doi:10.1007/s10549-012-2123-4

19. Casasent AK, Edgerton M, Navin NE. Genome evolution in ductal carcinoma in situ: invasion of the clones. *The Journal of Pathology*. 2016;241(2):208-218. doi:10.1002/path.4840

20. Yates LR, Knappskog S, Wedge D, et al. Genomic Evolution of Breast Cancer Metastasis and Relapse. *Cancer Cell*. Aug 14 2017;32(2):169-184 e7. doi:10.1016/j.ccell.2017.07.005

21. Casasent AK, Schalck A, Gao R, et al. Multiclonal Invasion in Breast Tumors Identified by Topographic Single Cell Sequencing. *Cell*. Jan 11 2018;172(1-2):205-217 e12. doi:10.1016/j.cell.2017.12.007

22. Lamy PJ, Fina F, Bascoul-Mollevi C, et al. Quantification and clinical relevance of gene amplification at chromosome 17q12-q21 in human epidermal growth factor receptor 2-amplified breast cancers. *Breast Cancer Res*. Feb 2 2011;13(1):R15. doi:10.1186/bcr2824

23. Xu L, Dai W, Li J, et al. Methylation-regulated miR-124-1 suppresses tumorigenesis in hepatocellular carcinoma by targeting CASC3. *Oncotarget*. May 3 2016;7(18):26027-41. doi:10.18632/oncotarget.8266

24. Marchesini M, Ogoti Y, Fiorini E, et al. ILF2 Is a Regulator of RNA Splicing and DNA Damage Response in 1q21-Amplified Multiple Myeloma. *Cancer Cell*. Jul 10 2017;32(1):88-100.e6. doi:10.1016/j.ccell.2017.05.011

25. Lu P, Foley J, Zhu C, et al. Transcriptome and genome evolution during HER2-amplified breast neoplasia. *Breast Cancer Research*. 2021;23(1)doi:10.1186/s13058-021-01451-6

26. Cowell CF, Weigelt B, Sakr RA, et al. Progression from ductal carcinoma in situ to invasive breast cancer: revisited. *Mol Oncol*. Oct 2013;7(5):859-69. doi:10.1016/j.molonc.2013.07.005

27. Melendez-Florez MP, Ortega-Recalde O, Rangel N, Rondon-Lagos M. Chromosomal Instability and Clonal Heterogeneity in Breast Cancer: From Mechanisms to Clinical Applications. *Cancers (Basel)*. Apr 4 2025;17(7)doi:10.3390/cancers17071222

28. Castellanos G, Camargo-Herrera LV, Rangel N, Jimenez-Tobon GA, Martinez-Aguero M, Rondon-Lagos M. Exploring chromosomal instability and clonal heterogeneity in breast cancer. *Endocr Relat Cancer*. Dec 1 2024;31(12)doi:10.1530/ERC-24-0096

29. Xu H, Lien T, Bergholtz H, et al. Multi-Omics Marker Analysis Enables Early Prediction of Breast Tumor Progression. *Front Genet*. 2021;12:670749. doi:10.3389/fgene.2021.670749

30. Casasent AK, Schalck A, Gao R, et al. Multiclonal Invasion in Breast Tumors Identified by Topographic Single Cell Sequencing. *Cell*. 2018;172(1-2):205-217.e12. doi:10.1016/j.cell.2017.12.007

31. Trinh A, Gil Del Alcazar CR, Shukla SA, et al. Genomic Alterations during the In Situ to Invasive Ductal Breast Carcinoma Transition Shaped by the Immune System. *Mol Cancer Res*. Apr 2021;19(4):623-635. doi:10.1158/1541-7786.MCR-20-0949

32. Thorat MA, Levey PM, Jones JL, et al. Prognostic and Predictive Value of HER2 Expression in Ductal Carcinoma In Situ: Results from the UK/ANZ DCIS Randomized Trial. *Clin Cancer Res*. Oct 1 2021;27(19):5317-5324. doi:10.1158/1078-0432.CCR-21-1239

33. Nagasawa S, Kuze Y, Maeda I, et al. Genomic profiling reveals heterogeneous populations of ductal carcinoma in situ of the breast. *Commun Biol*. Apr 1 2021;4(1):438. doi:10.1038/s42003-021-01959-9

34. Varis A, Wolf M, Monni O, et al. Targets of gene amplification and overexpression at 17q in gastric cancer. *Cancer Res*. May 1 2002;62(9):2625-9.

35. Brunner AL, Li J, Guo X, et al. A shared transcriptional program in early breast neoplasias despite genetic and clinical distinctions. *Genome Biol*. May 23 2014;15(5):R71. doi:10.1186/gb-2014-15-5-r71

36. Xi Z, Huang H, Hu J, et al. LINC00571 drives tricarboxylic acid cycle metabolism in triple-negative breast cancer through HNRNPK/ILF2/IDH2 axis. *J Exp Clin Cancer Res*. Jan 18 2024;43(1):22. doi:10.1186/s13046-024-02950-y

37. Zhao M, Liu Y, Chang J, et al. ILF2 cooperates with E2F1 to maintain mitochondrial homeostasis and promote small cell lung cancer progression. *Cancer Biol Med*. Nov 2019;16(4):771-783. doi:10.20892/j.issn.2095-3941.2019.0050

38. Li S, Zhang W, Yang Q, et al. Genomic landscape of paired primary and peritoneal metastatic lesions in gastric cancer highlights evolutionary dynamics and mutational drivers. *J Adv Res*. May 26 2025;doi:10.1016/j.jare.2025.05.043

39. Zhao K, Vos J, Lam S, et al. Longitudinal and multisite sampling reveals mutational and copy number evolution in tumors during metastatic dissemination. *Nat Genet*. Jun 2025;57(6):1504-1511. doi:10.1038/s41588-025-02204-3

40. Chen D, Lu S, Huang K, et al. Cell cycle duration determines oncogenic transformation capacity. *Nature*. May 2025;641(8065):1309-1318. doi:10.1038/s41586-025-08935-x

41. Lopes Cardozo JMN, Andrulis IL, Bojesen SE, et al. Associations of a Breast Cancer Polygenic Risk Score With Tumor Characteristics and Survival. *J Clin Oncol*. Apr 1 2023;41(10):1849-1863. doi:10.1200/JCO.22.01978

42. Nilsen G, Liestol K, Van Loo P, et al. Copynumber: Efficient algorithms for single- and multi-track copy number segmentation. *BMC Genomics*. Nov 4 2012;13:591. doi:10.1186/1471-2164-13-591

43. Bailey MH, Tokheim C, Porta-Pardo E, et al. Comprehensive Characterization of Cancer Driver Genes and Mutations. *Cell*. Apr 5 2018;173(2):371-385 e18. doi:10.1016/j.cell.2018.02.060

44. Nik-Zainal S, Davies H, Staaf J, et al. Landscape of somatic mutations in 560 breast cancer whole-genome sequences. *Nature*. Jun 2 2016;534(7605):47-54. doi:10.1038/nature17676


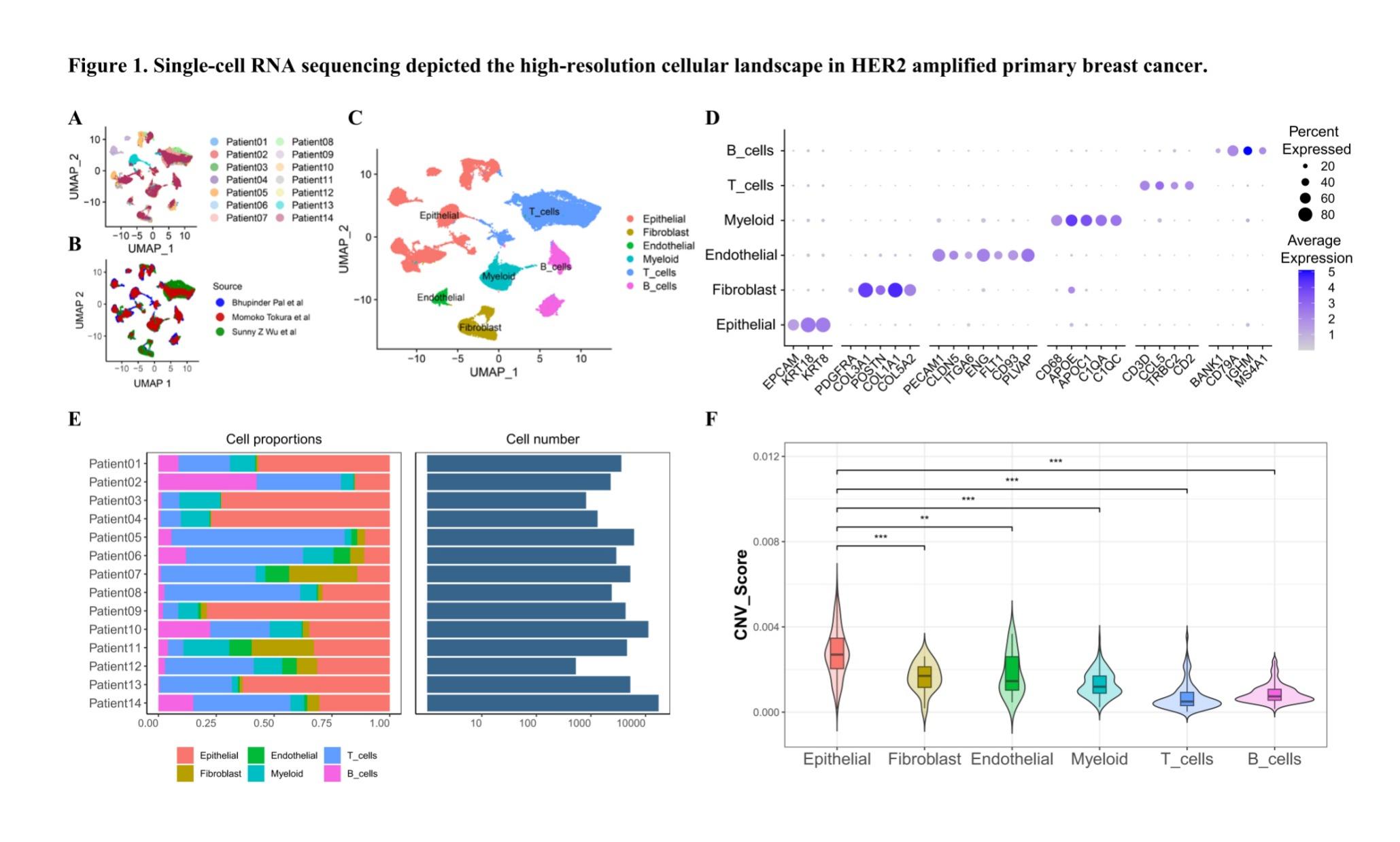


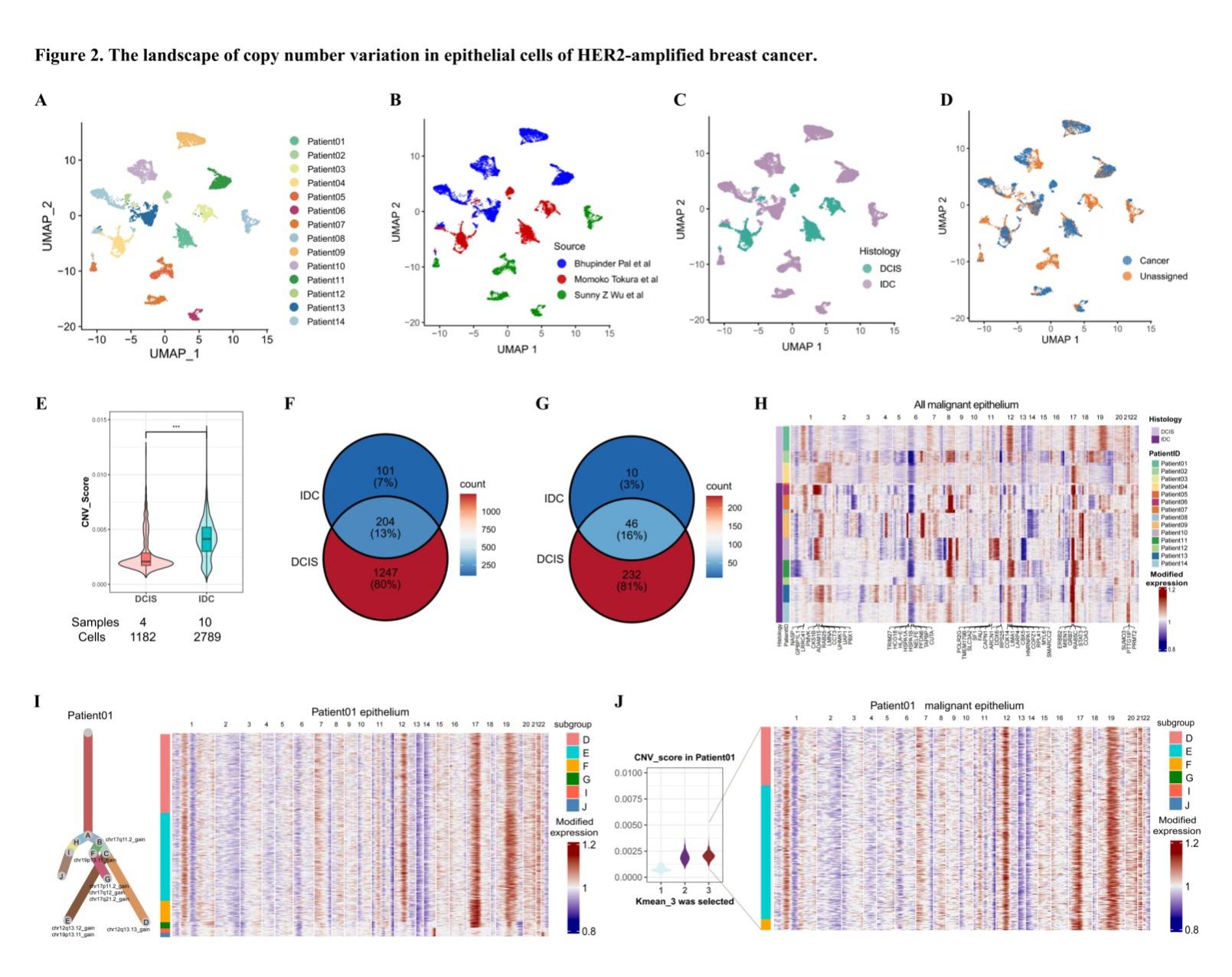


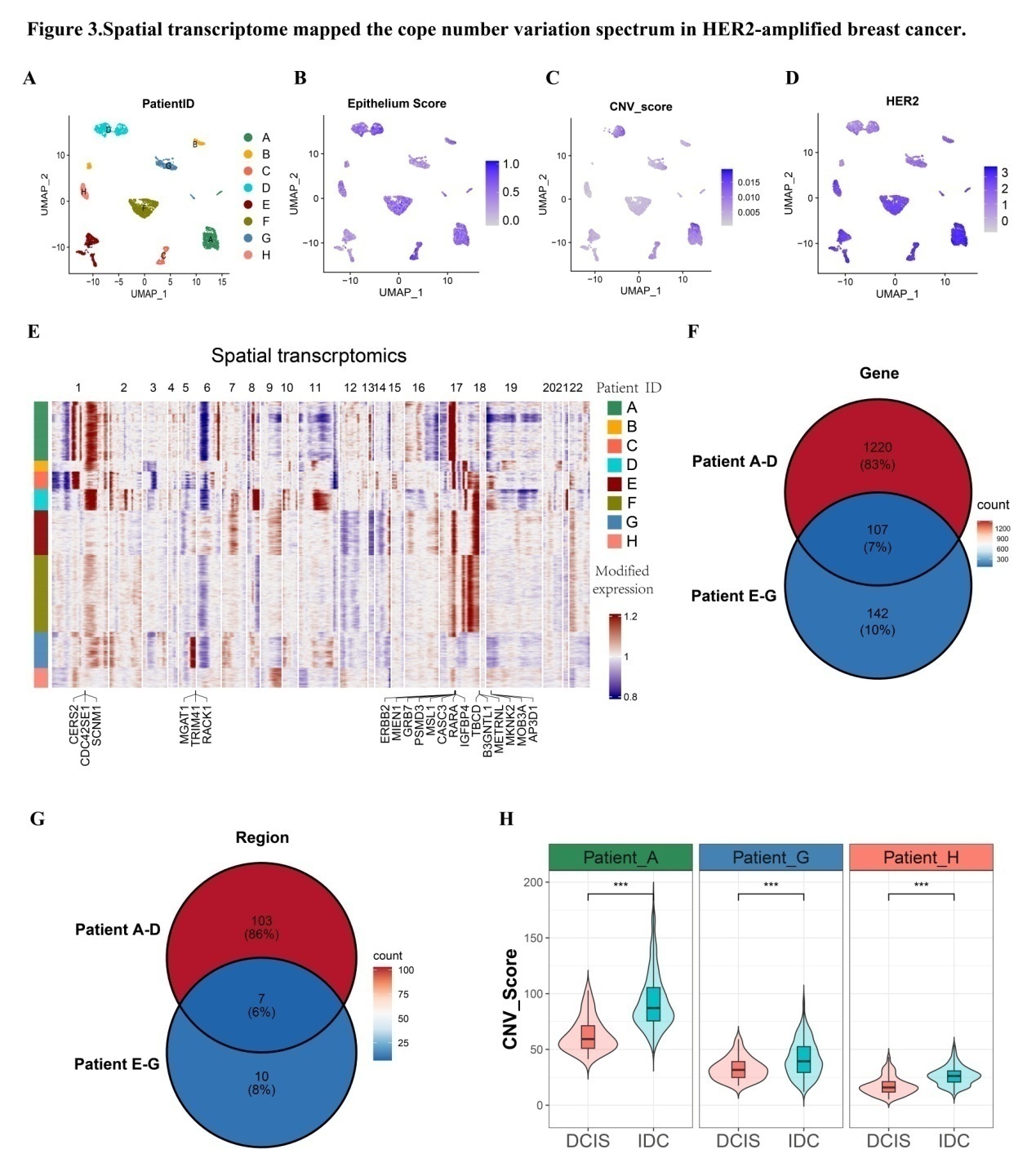


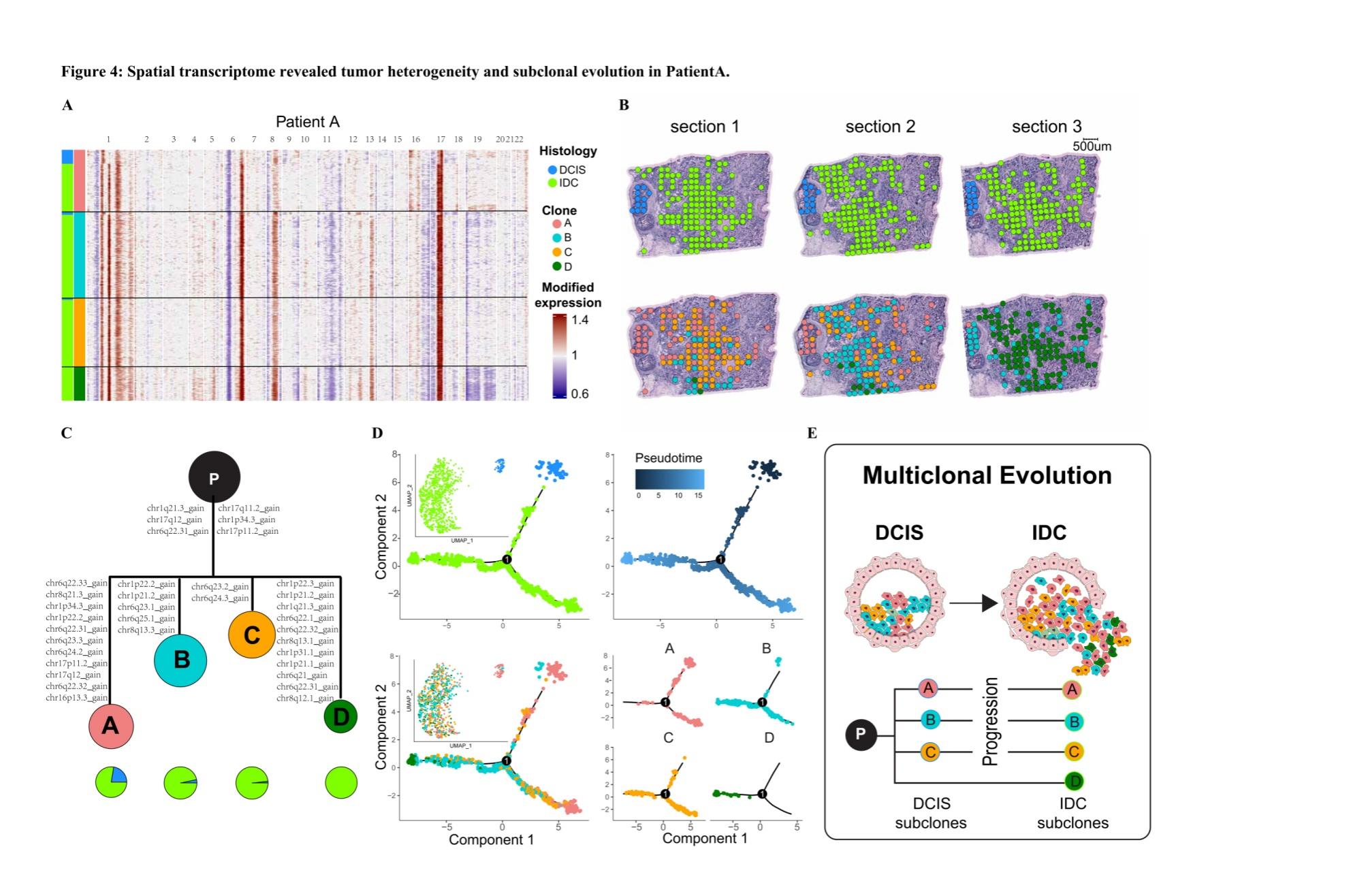

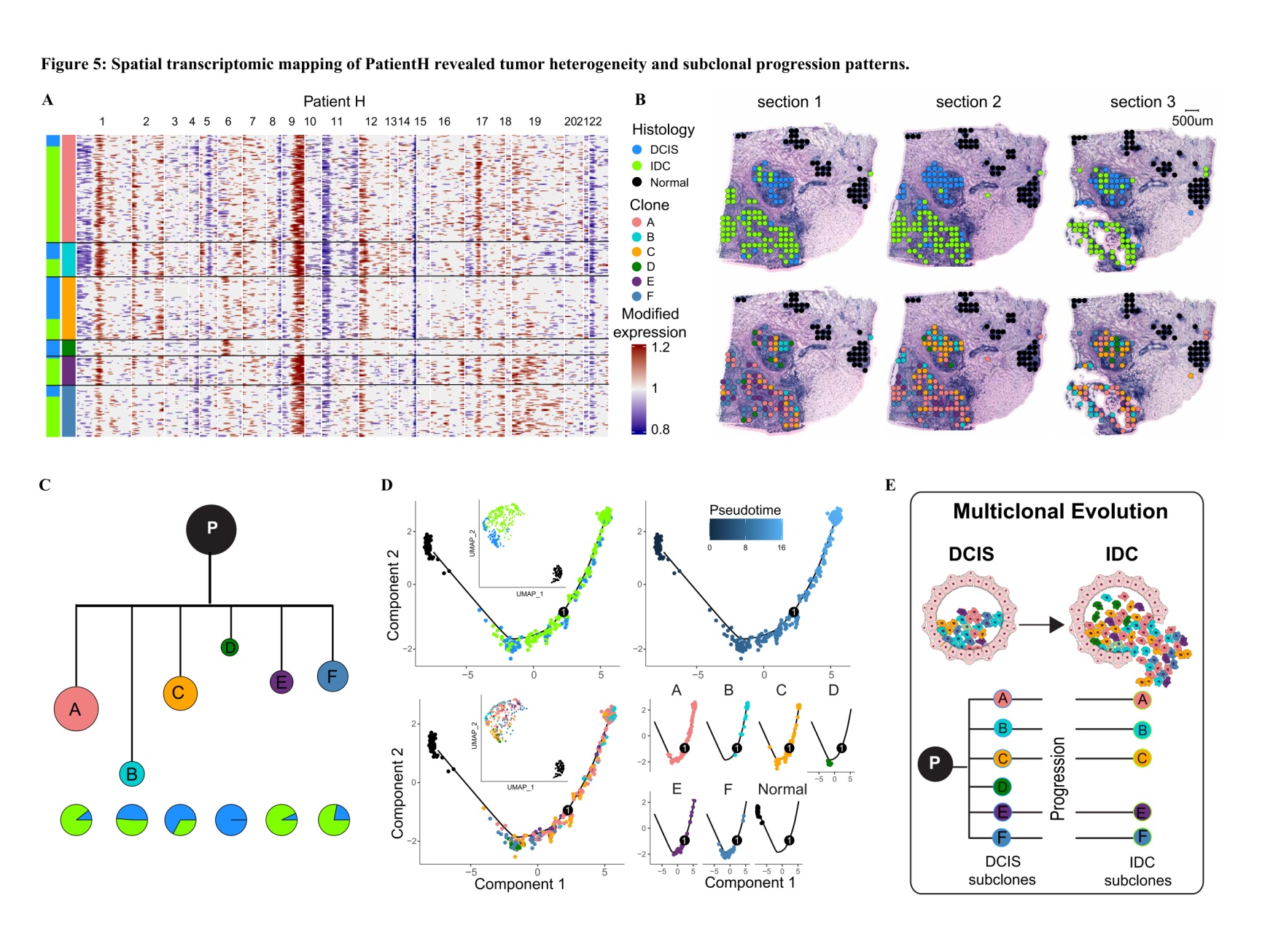

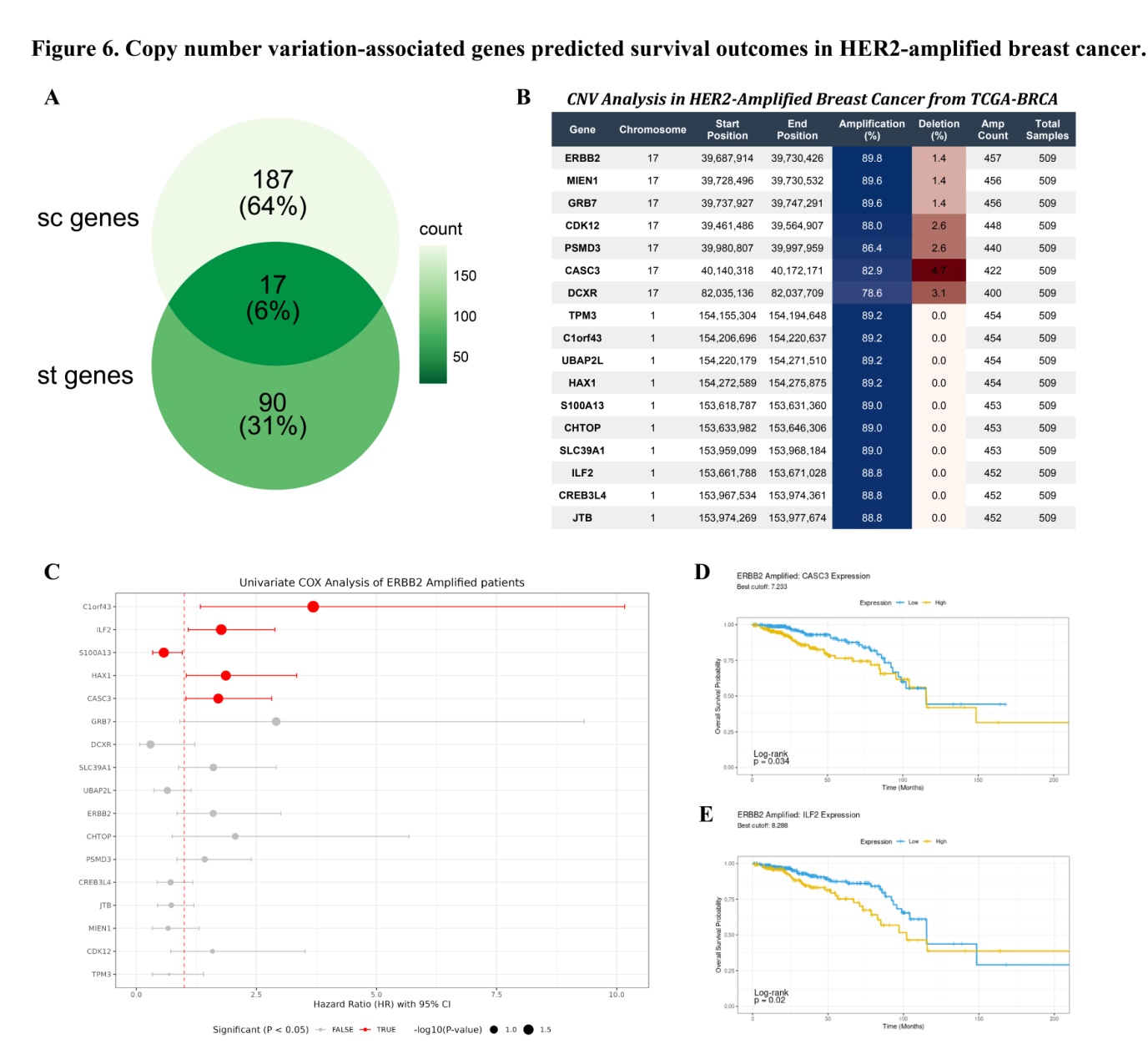

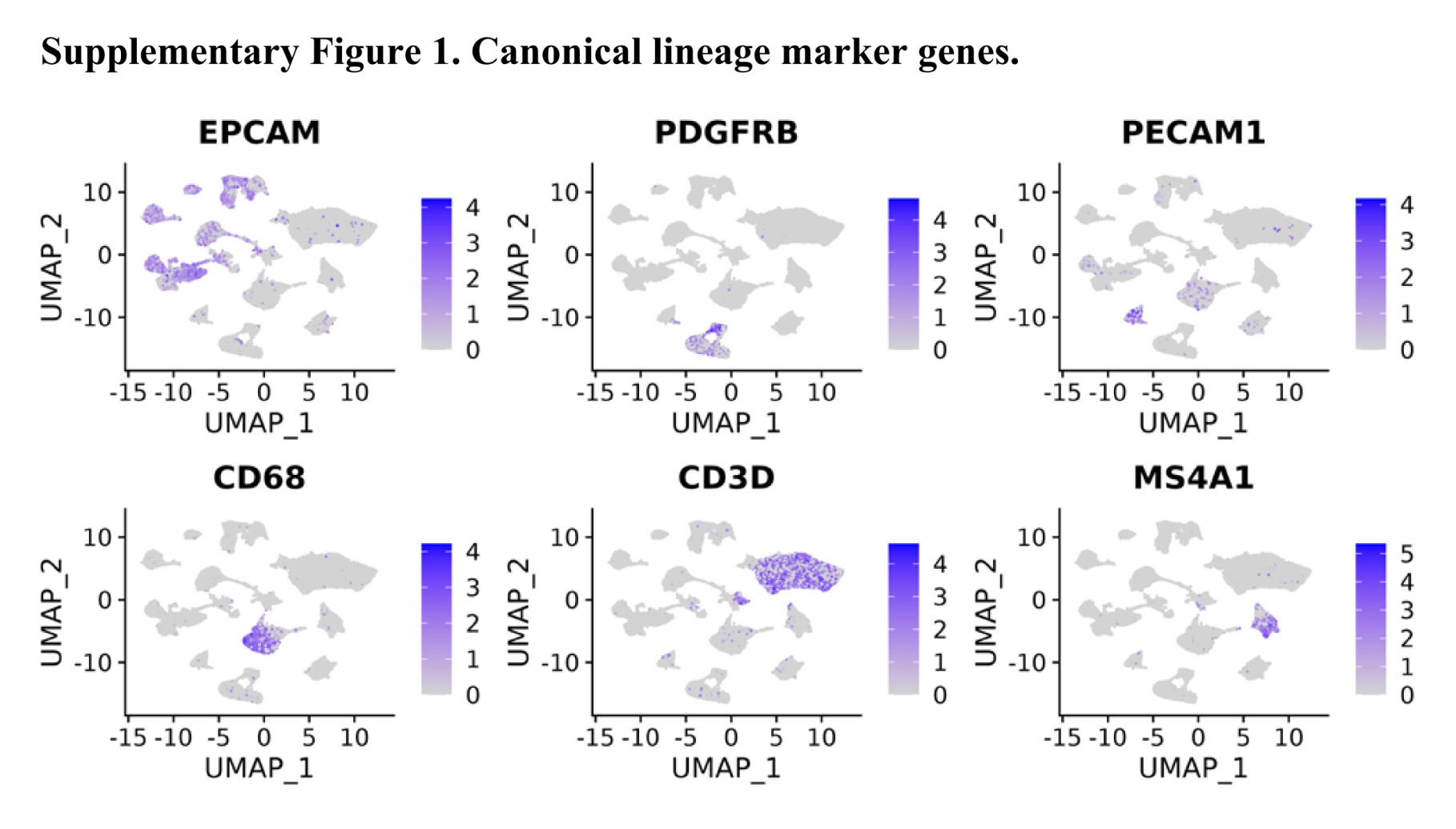

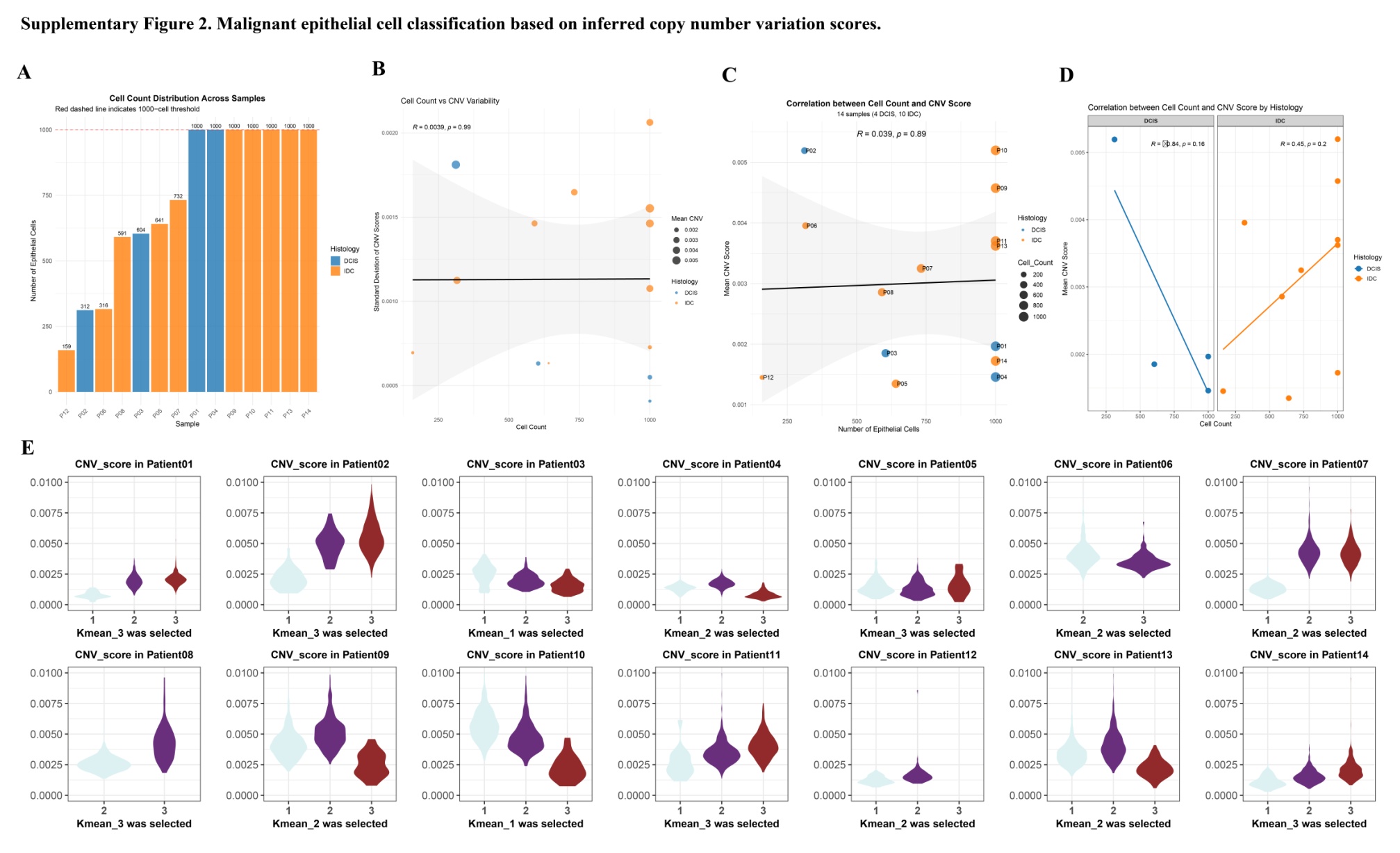


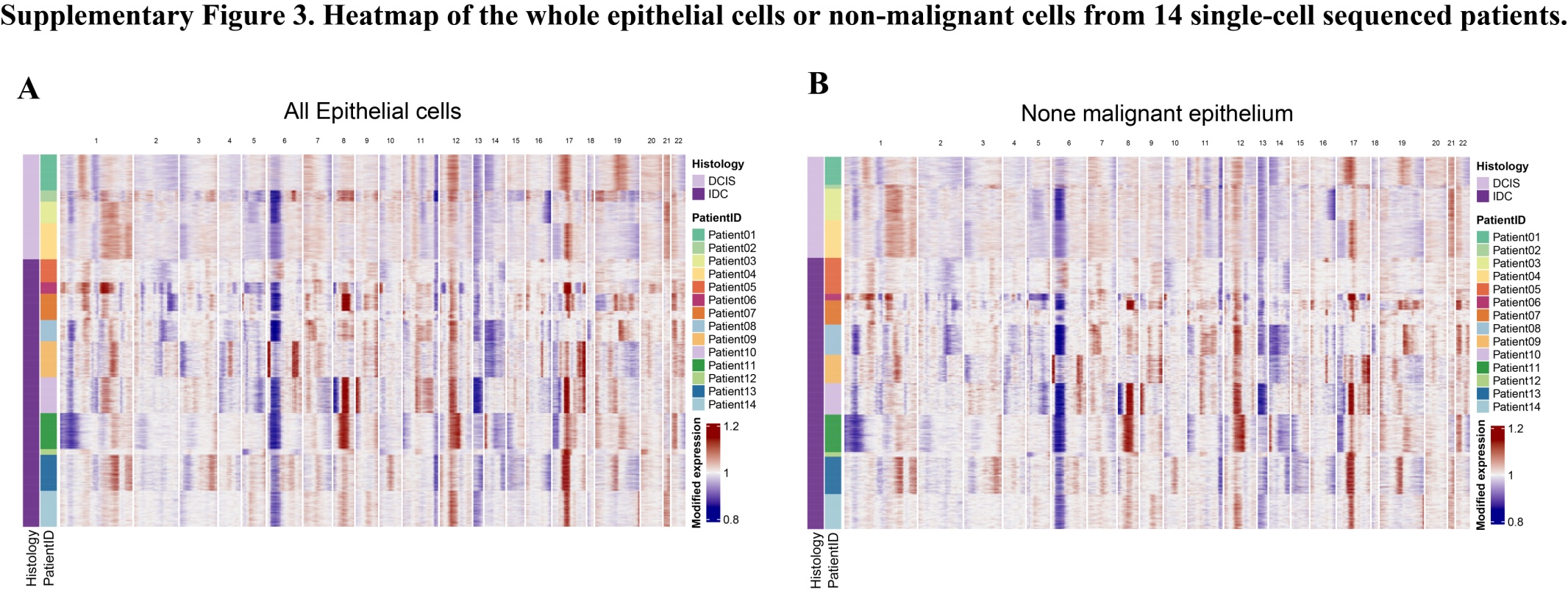

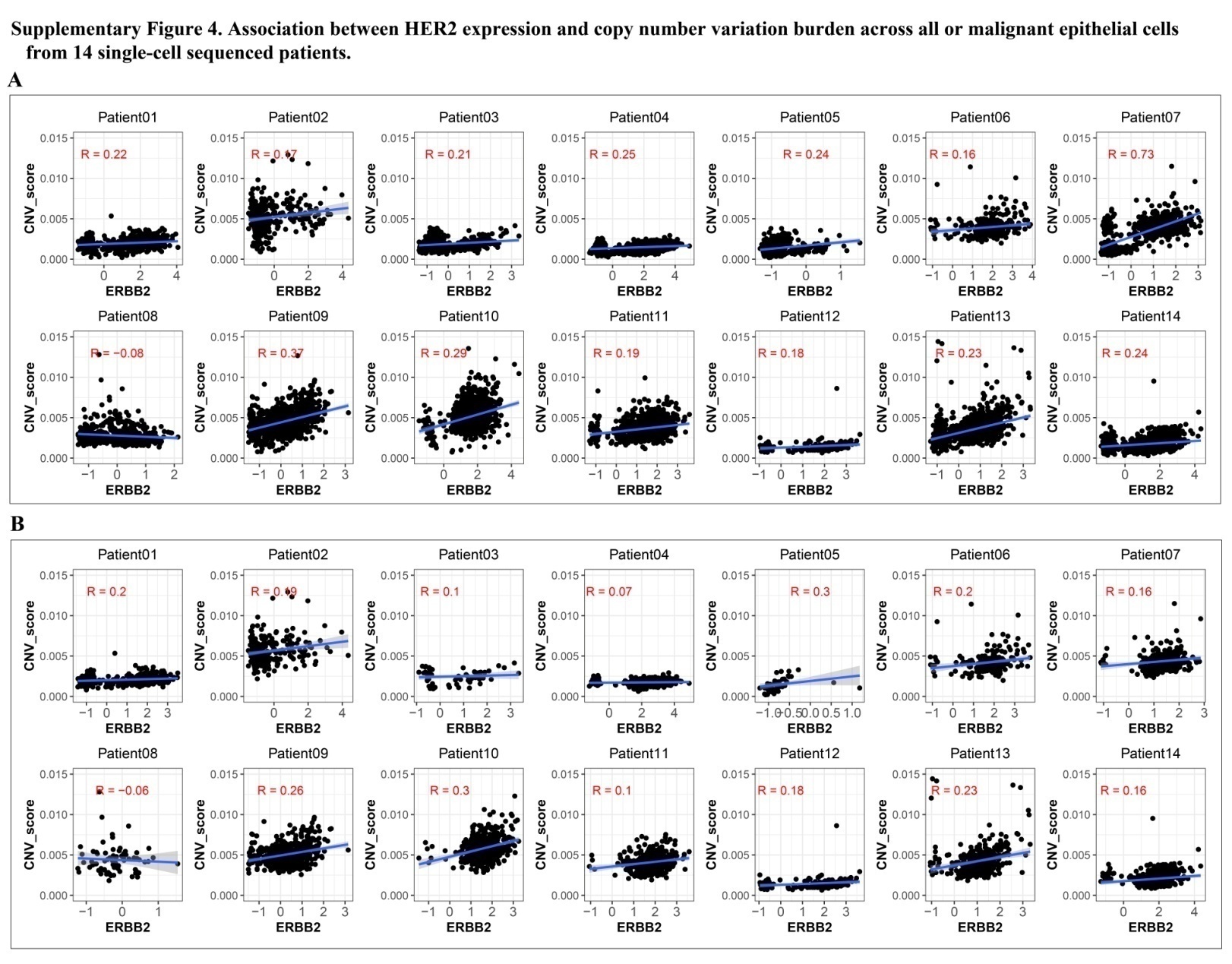


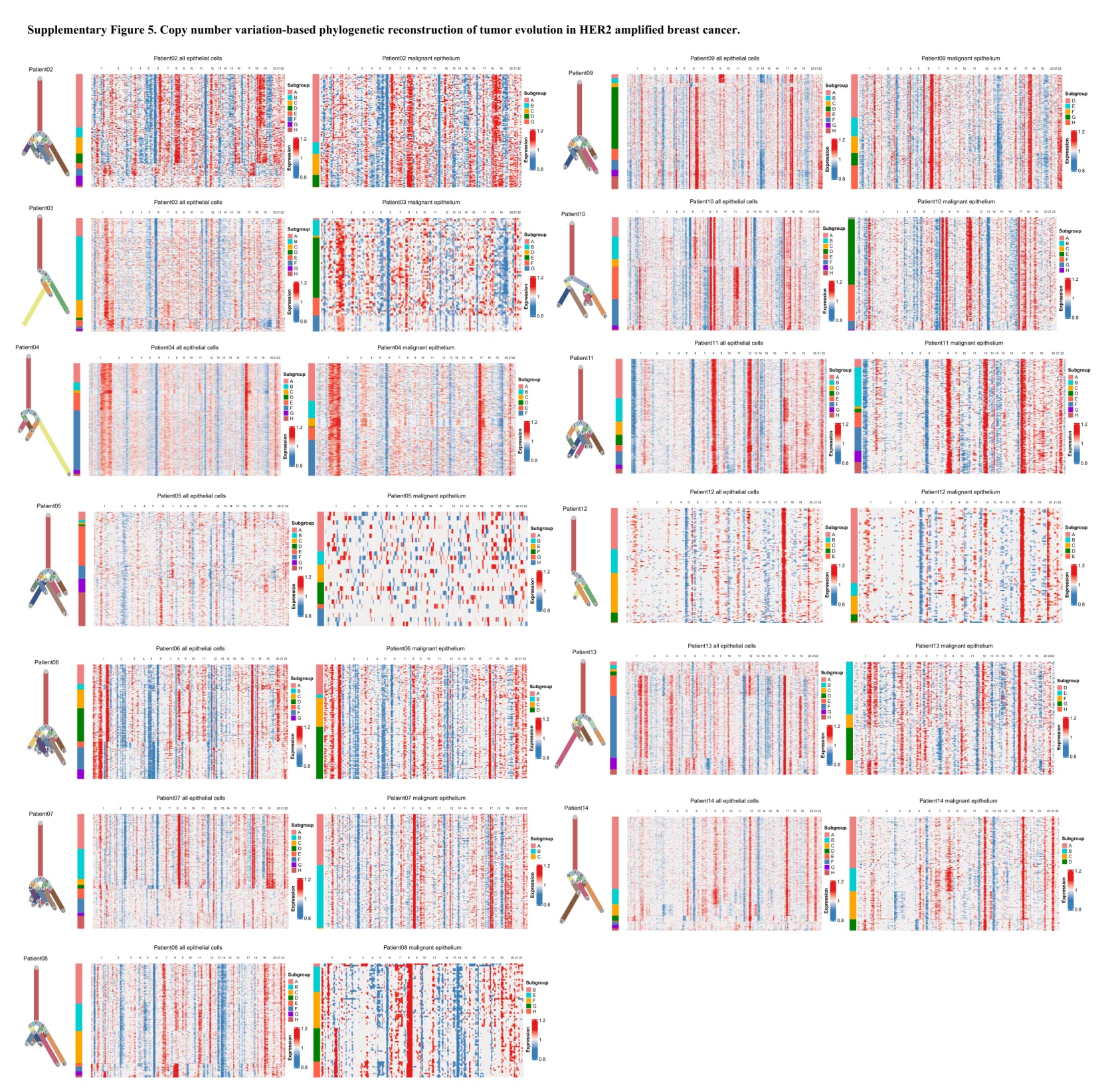


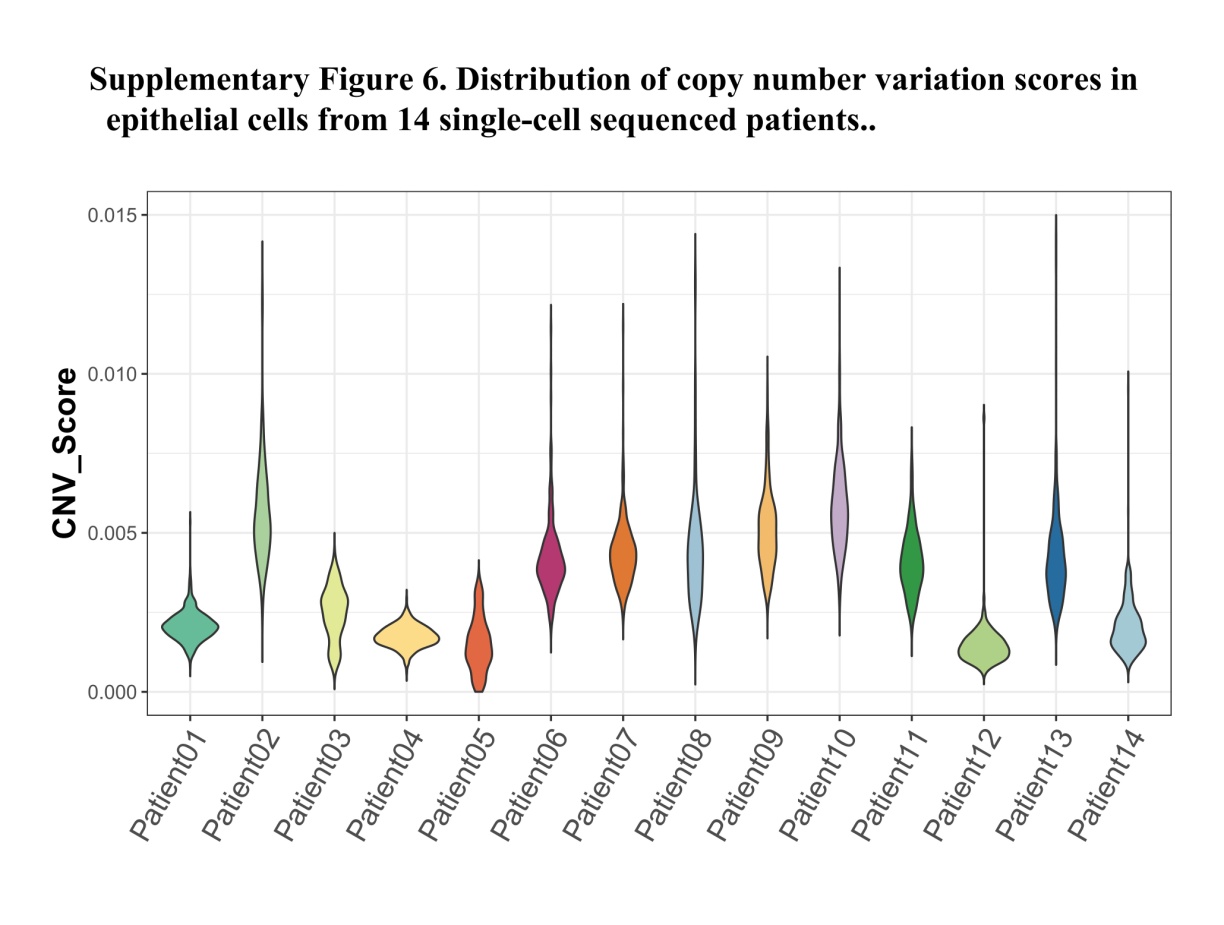

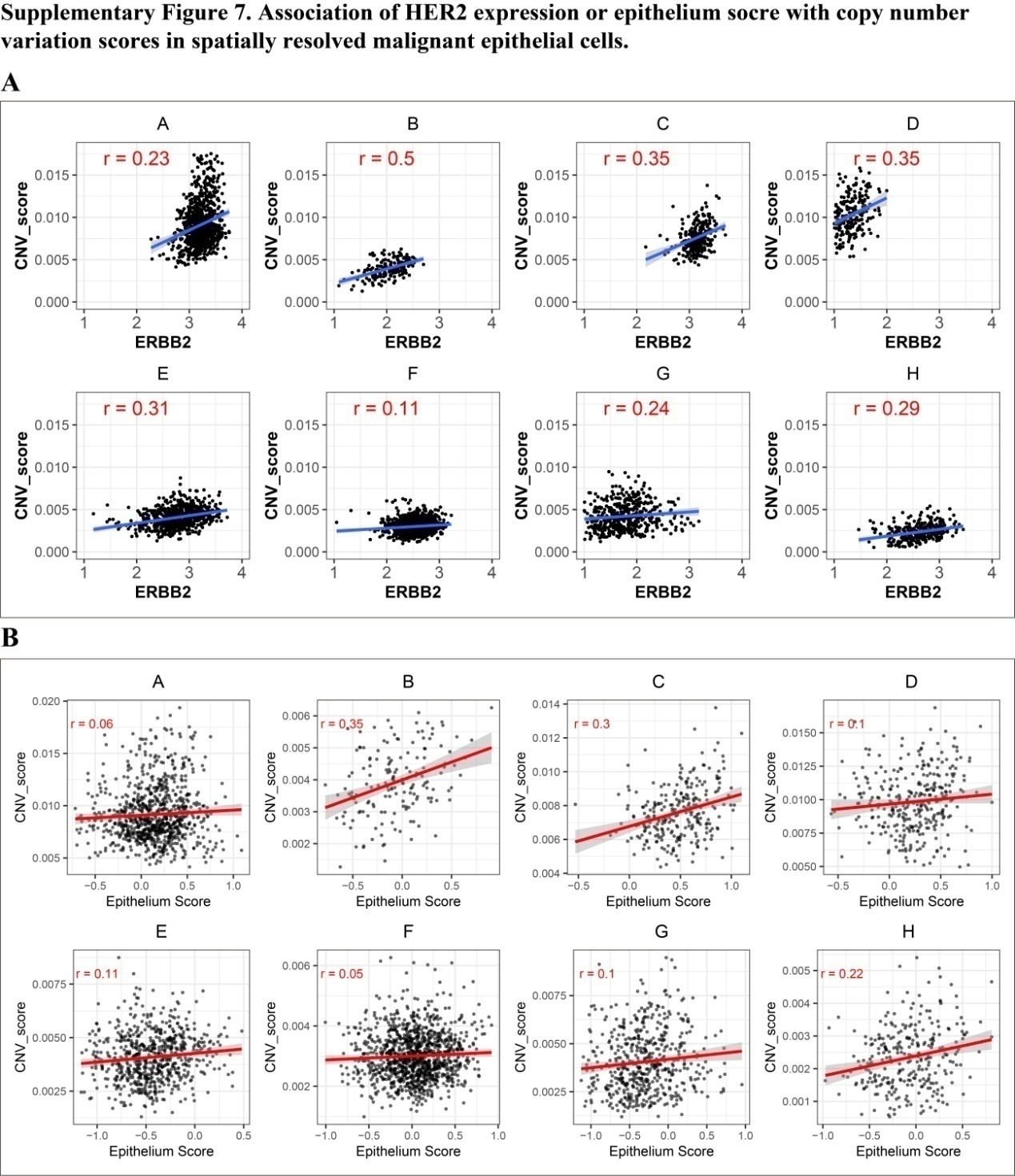


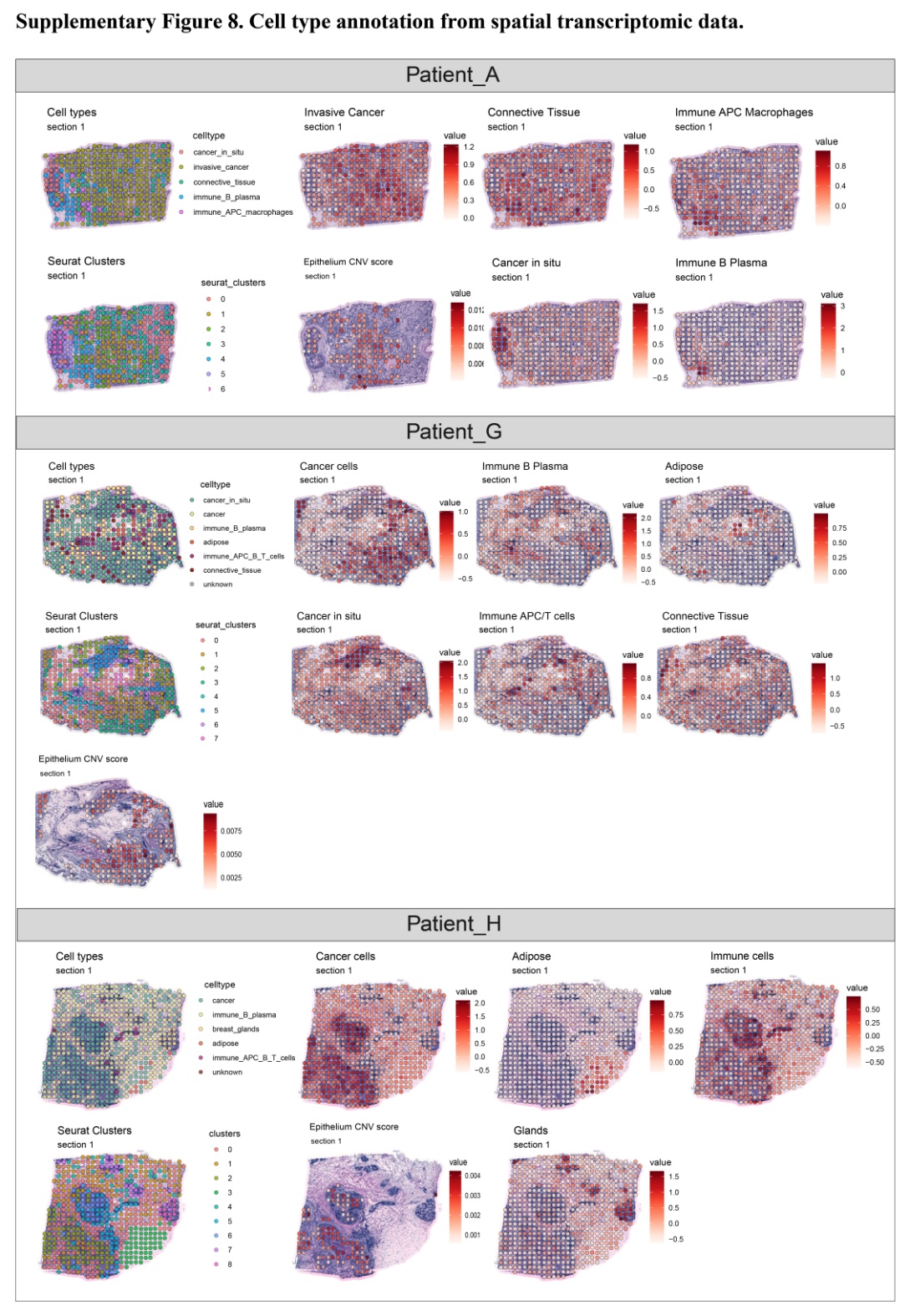


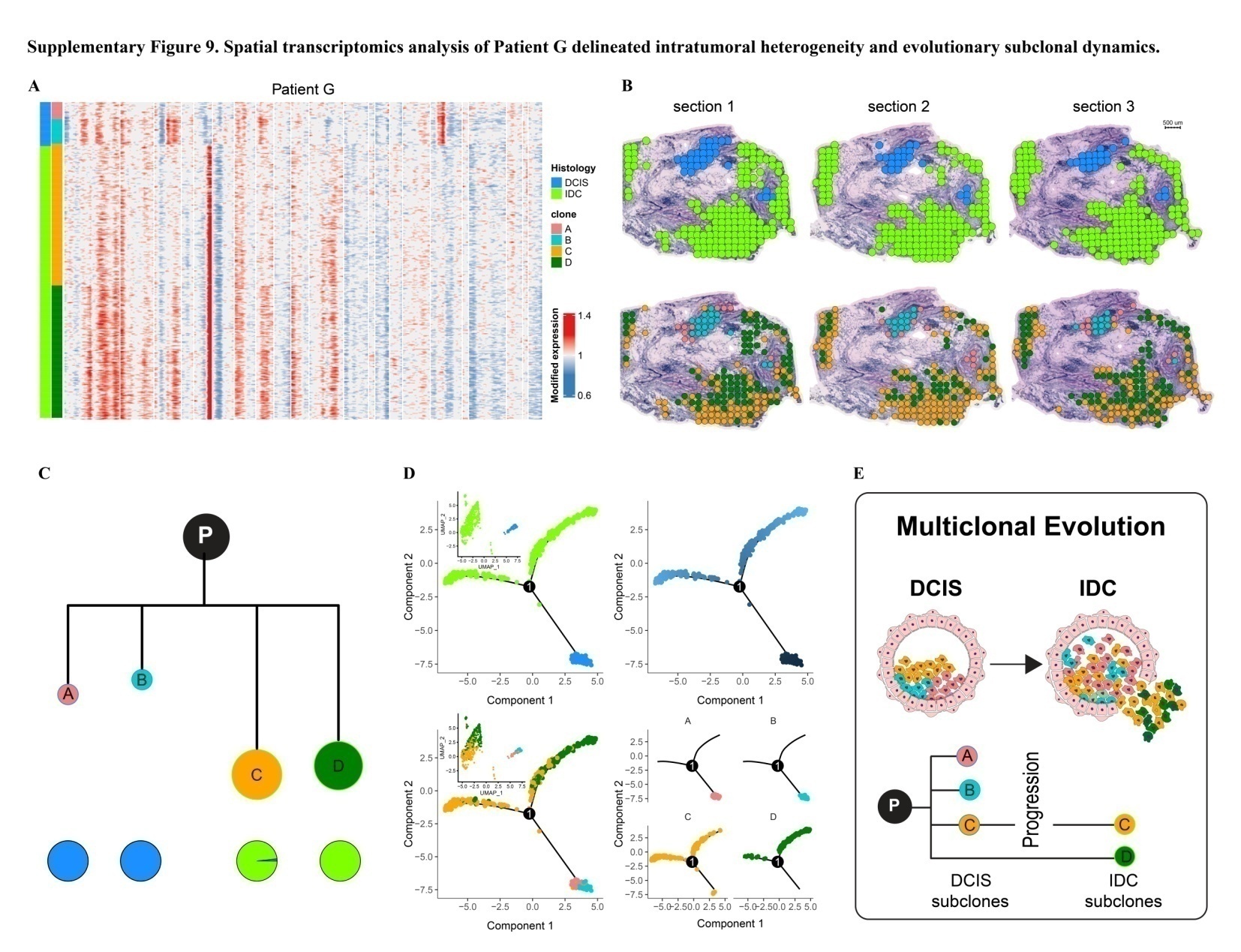


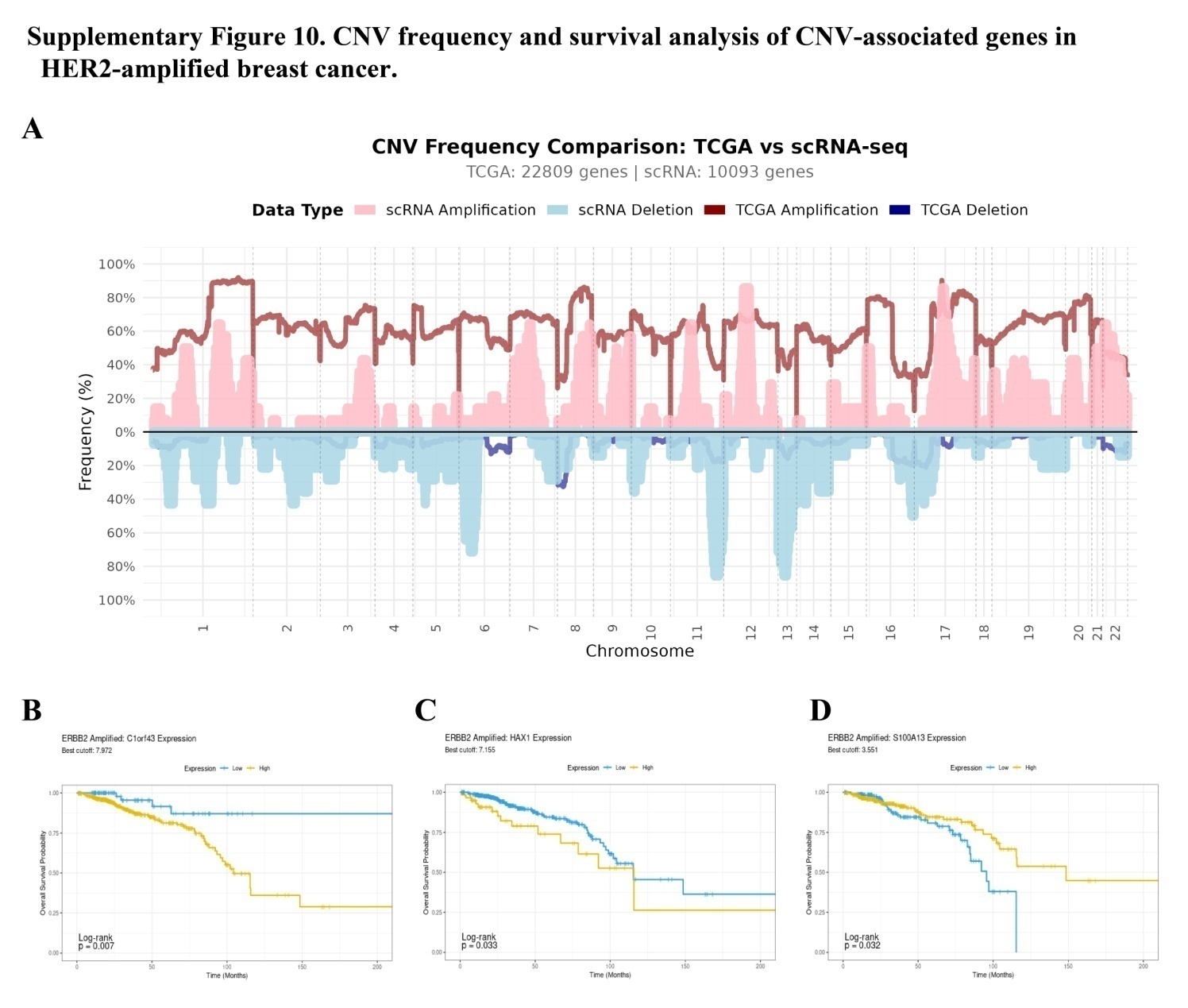


Supplementary Table 1. Clinical and pathological information of all patients.

| **PatientID** | **Source** | **SeqName** | **SelectionCell** | **Technique** | **Gender** | **Age** | **Histology** | **Grade** | **Tumor Type** |
| --- | --- | --- | --- | --- | --- | --- | --- | --- | --- |
| Patient01 | Momoko Tokura et al | NCCBC05 | Total | 10x | Female | 44 | DCIS* | 3 | HER2 amplified |
| Patient02 | Momoko Tokura et al | NCCBC06 | Total | 10x | Female | 60 | DCIS | 2 | HER2 amplified |
| Patient03 | Momoko Tokura et al | NCCBC11 | Total | 10x | Female | 43 | DCIS | 1 | HER2 amplified |
| Patient04 | Momoko Tokura et al | NCCBC14 | Total | 10x | Female | 39 | DCIS | 3 | HER2 amplified |
| Patient05 | Sunny Z Wu et al | CID3586 | Total | 10x | Female | 43 | IDC* | 3 | HER2 amplified |
| Patient06 | Sunny Z Wu et al | CID3921 | Total | 10x | Female | 60 | IDC | 3 | HER2 amplified |
| Patient07 | Sunny Z Wu et al | CID4066 | Total | 10x | Female | 41 | IDC | 2 | HER2 amplified |
| Patient08 | Sunny Z Wu et al | CID45171 | Total | 10x | Female | 58 | IDC | 3 | HER2 amplified |
| Patient09 | Bhupinder Pal et al | GSM4909289_HER2-AH0308 | Total | 10x | Female | 32 | IDC | 3 | HER2 amplified |
| Patient10 | Bhupinder Pal et al | GSM4909290_HER2-PM0337 | Total | 10x | Female | 66 | IDC | 3 | HER2 amplified |
| Patient11 | Bhupinder Pal et al | GSM4909291_HER2-MH0031 | Total | 10x | Female | 47 | IDC | 3 | HER2 amplified |
| Patient12 | Bhupinder Pal et al | GSM4909292_HER2-MH0069 | Total | 10x | Female | 71 | IDC | 3 | HER2 amplified |
| Patient13 | Bhupinder Pal et al | GSM4909293_HER2-MH0161 | Total | 10x | Female | 80 | IDC | 3 | HER2 amplified |
| Patient14 | Bhupinder Pal et al | GSM4909294_HER2-MH0176 | Total | 10x | Female | 60 | IDC | 3 | HER2 amplified |
| PatientA | Alma Andersson et al | PatientA | Total | Spatial Transcriptomics | Na* | Na | DCIS/IDC | Na | HER2 amplified |
| PatientB | Alma Andersson et al | PatientB | Total | Spatial Transcriptomics | Na | Na | IDC | 3 | HER2 amplified |
| PatientC | Alma Andersson et al | PatientC | Total | Spatial Transcriptomics | Na | Na | IDC | 3 | HER2 amplified |
| PatientD | Alma Andersson et al | PatientD | Total | Spatial Transcriptomics | Na | Na | IDC | Na | HER2 amplified |
| PatientE | Alma Andersson et al | PatientE | Total | Spatial Transcriptomics | Na | Na | IDC | 3 | HER2 amplified |
| PatientF | Alma Andersson et al | PatientF | Total | Spatial Transcriptomics | Na | Na | IDC | 2 | HER2 amplified |
| PatientG | Alma Andersson et al | PatientG | Total | Spatial Transcriptomics | Na | Na | DCIS/IDC | 3 | HER2 amplified |
| PatientH | Alma Andersson et al | PatientH | Total | Spatial Transcriptomics | Na | Na | DCIS/IDC | 3 | HER2 amplified |
| *Na: not applicable; DCIS: ductal carcinoma in situ; IDC: invasive ductal carcinoma. | | | | | | | | | |

Supplementary Table 2. Tumor subclonal architecture reconstruction in Patient A via copy number variation (CNV) profiling.

| **CNV common** | **Clone A** | **Clone B** | **Clone C** | **Clone D** |
| --- | --- | --- | --- | --- |
| chr1p34.3_amplification | chr6p22.3_deletion | chr1p22.2_amplification | chr5q22.3_amplification | chr1p22.3_amplification |
| chr1q21.3_amplification | chr7q11.21_amplification | chr1p21.2_amplification | chr6q21_amplification | chr1p21.2_amplification |
| chr6p22.3_deletion | chr9p24.3_deletion | chr4p14_amplification | chr6q23.2_amplification | chr1q23.3_amplification |
| chr6p21.1_amplification | chr11q21_deletion | chr4q24_amplification | chr6q24.3_amplification | chr2q32.1_amplification |
| chr6q22.31_amplification | chr12q13.13_amplification | chr5q21.3_amplification | chr8q12.1_amplification | chr3p25.2_amplification |
| chr8p23.3_deletion | chr16p12.2_amplification | chr6q23.1_amplification | chr11q13.1_deletion | chr4p15.2_amplification |
| chr17p13.3_deletion | chr17q21.2_deletion | chr6q25.1_amplification | chr11q21_deletion | chr5q13.3_amplification |
| chr17p11.2_amplification | chr22q13.1_amplification | chr8q11.23_amplification | chr12p13.33_amplification | chr6q22.1_amplification |
| chr17q11.2_amplification | chr1p36.13_deletion | chr8q13.3_amplification | chr13q32.3_deletion | chr6q22.32_amplification |
| chr17q12_amplification | chr1p32.3_amplification | chr8q24.12_amplification | chr14q32.32_deletion | chr8q11.23_amplification |
| chr17q21.2_amplification | chr1p22.2_amplification | chr9q34.13_deletion | chr16p13.3_amplification | chr8q13.1_amplification |
| chr2p25.3_amplification | chr1q23.1_amplification | chr10q11.23_amplification | 0 | chr11p11.2_deletion |
| chr19p13.3_deletion | chr2p25.3_amplification | chr11p15.5_deletion | 0 | chr12q14.3_amplification |
| chr1p34.1_amplification | chr2q37.1_amplification | chr11q22.1_deletion | 0 | chr16p13.13_amplification |
| chr1p31.1_amplification | chr6q21_amplification | chr12p11.23_amplification | 0 | chr17q21.1_amplification |
| chr1p13.3_amplification | chr6q22.33_amplification | chr12q15_amplification | 0 | chr18q21.2_amplification |
| chr1q23.3_amplification | chr6q24.3_amplification | chr13q33.1_deletion | 0 | chr20q11.23_deletion |
| chr13q12.11_amplification | chr7q36.1_deletion | chr14q32.31_deletion | 0 | chr1p21.1_amplification |
| chr1p22.3_amplification | chr8q13.1_amplification | chr15q26.1_deletion | 0 | chr2q31.2_amplification |
| chr11p11.2_deletion | chr8q21.3_amplification | chr17q25.1_deletion | 0 | chr3p14.3_amplification |
| 0 | chr8q24.13_amplification | chr18q21.33_amplification | 0 | chr3q25.1_amplification |
| 0 | chr9q31.2_amplification | chr19p13.11_deletion | 0 | chr4p15.31_amplification |
| 0 | chr11p15.5_deletion | chr21q22.3_deletion | 0 | chr5p15.33_amplification |
| 0 | chr12p13.31_amplification | 0 | 0 | chr5q13.2_amplification |
| 0 | chr12q24.11_deletion | 0 | 0 | chr6q21_amplification |
| 0 | chr14q32.32_deletion | 0 | 0 | chr6q22.31_amplification |
| 0 | chr16q24.2_deletion | 0 | 0 | chr8q11.21_amplification |
| 0 | chr19p13.3_deletion | 0 | 0 | chr8q12.1_amplification |
| 0 | chr1p36.12_deletion | 0 | 0 | chr11p12_deletion |
| 0 | chr1p34.1_amplification | 0 | 0 | chr12q14.1_amplification |
| 0 | chr1p31.1_amplification | 0 | 0 | chr16p13.12_amplification |
| 0 | chr1p13.3_amplification | 0 | 0 | chr17q21.2_amplification |
| 0 | chr1q23.3_amplification | 0 | 0 | chr18q21.1_amplification |
| 0 | chr2p11.2_deletion | 0 | 0 | chr22q11.21_deletion |
| 0 | chr6p23_deletion | 0 | 0 | 0 |
| 0 | chr6p21.1_amplification | 0 | 0 | 0 |
| 0 | chr6q22.31_amplification | 0 | 0 | 0 |
| 0 | chr6q23.3_amplification | 0 | 0 | 0 |
| 0 | chr6q24.2_amplification | 0 | 0 | 0 |
| 0 | chr6q25.2_amplification | 0 | 0 | 0 |
| 0 | chr8q12.2_amplification | 0 | 0 | 0 |
| 0 | chr12p13.33_amplification | 0 | 0 | 0 |
| 0 | chr13q12.11_amplification | 0 | 0 | 0 |
| 0 | chr13q32.1_deletion | 0 | 0 | 0 |
| 0 | chr15q26.1_deletion | 0 | 0 | 0 |
| 0 | chr16p12.3_amplification | 0 | 0 | 0 |
| 0 | chr16q12.1_deletion | 0 | 0 | 0 |
| 0 | chr17q12_amplification | 0 | 0 | 0 |
| 0 | chr17q25.3_amplification | 0 | 0 | 0 |
| 0 | chr22q13.2_amplification | 0 | 0 | 0 |
| 0 | chr1p36.33_amplification | 0 | 0 | 0 |
| 0 | chr1p22.3_amplification | 0 | 0 | 0 |
| 0 | chr1q23.2_amplification | 0 | 0 | 0 |
| 0 | chr4p16.3_amplification | 0 | 0 | 0 |
| 0 | chr5q35.2_amplification | 0 | 0 | 0 |
| 0 | chr6p24.3_deletion | 0 | 0 | 0 |
| 0 | chr6q22.1_amplification | 0 | 0 | 0 |
| 0 | chr6q22.32_amplification | 0 | 0 | 0 |
| 0 | chr6q25.1_amplification | 0 | 0 | 0 |
| 0 | chr7q22.1_amplification | 0 | 0 | 0 |
| 0 | chr9q34.3_amplification | 0 | 0 | 0 |
| 0 | chr11p11.2_deletion | 0 | 0 | 0 |
| 0 | chr11q14.2_deletion | 0 | 0 | 0 |
| 0 | chr13q31.1_deletion | 0 | 0 | 0 |
| 0 | chr14q23.1_deletion | 0 | 0 | 0 |
| 0 | chr14q32.33_deletion | 0 | 0 | 0 |
| 0 | chr19p13.3_amplification | 0 | 0 | 0 |
| 0 | chr19p13.11_amplification | 0 | 0 | 0 |

Supplementary Table 3. Clonal architecture deconvolution in Patient H using genome-wide copy number alteration patterns.

| **CNV common** | **Clone A** | **Clone B** | **Clone C** | **Clone D** | **Clone E** | **Clone F** |
| --- | --- | --- | --- | --- | --- | --- |
| chr14q32.33_deletion | chr1p34.3_amplification | chr1p36.33_deletion | chr1p34.3_amplification | chr1p34.2_amplification | chr1p34.3_amplification | chr1p34.3_amplification |
| chr9q34.11_amplification | chr11p15.5_deletion | chr1p34.3_amplification | chr11p15.5_deletion | chr1p13.1_amplification | chr11p15.5_deletion | chr11p15.5_deletion |
| 0 | chr11q22.2_deletion | chr1p34.2_amplification | chr12p13.33_amplification | chr14q32.33_deletion | chr12p13.33_amplification | chr12p13.33_amplification |
| 0 | chr12p13.33_amplification | chr1p32.3_amplification | chr14q32.33_deletion | chr6p22.1_amplification | chr14q32.33_deletion | chr14q32.33_deletion |
| 0 | chr14q32.33_deletion | chr11p15.5_deletion | chr17q11.2_amplification | chr6p21.33_amplification | chr16q23.1_amplification | chr16q24.2_amplification |
| 0 | chr17q11.2_amplification | chr11q13.4_deletion | chr2p25.3_amplification | chr6p21.32_amplification | chr17q11.2_amplification | chr17q11.2_amplification |
| 0 | chr19p13.3_amplification | chr12p13.33_amplification | chr2q33.1_amplification | chr9q34.11_amplification | chr17q25.1_amplification | chr17q25.3_amplification |
| 0 | chr2p25.3_amplification | chr12q23.1_amplification | chr22q11.1_deletion | 0 | chr19p13.3_amplification | chr19p13.3_amplification |
| 0 | chr2q35_amplification | chr13q14.2_amplification | chr5p15.33_amplification | 0 | chr2p25.3_amplification | chr2p25.3_amplification |
| 0 | chr22q11.1_deletion | chr14q11.2_amplification | chr6p21.33_amplification | 0 | chr22q11.1_deletion | chr4q12_deletion |
| 0 | chr5q31.1_deletion | chr14q32.33_deletion | chr7p22.3_amplification | 0 | chr4q12_deletion | chr6p21.33_amplification |
| 0 | chr7p22.3_amplification | chr17q11.2_amplification | chr8q24.3_deletion | 0 | chr7p11.2_amplification | chr9q34.11_amplification |
| 0 | chr8q24.3_deletion | chr17q25.3_amplification | chr9q34.11_amplification | 0 | chr9q33.3_amplification | chr9q34.3_amplification |
| 0 | chr9q33.3_amplification | chr2p25.3_amplification | 0 | 0 | chr9q34.11_amplification | 0 |
| 0 | chr9q34.11_amplification | chr2p24.1_amplification | 0 | 0 | 0 | 0 |
| 0 | 0 | chr2q33.2_amplification | 0 | 0 | 0 | 0 |
| 0 | 0 | chr21q22.3_deletion | 0 | 0 | 0 | 0 |
| 0 | 0 | chr22q11.1_deletion | 0 | 0 | 0 | 0 |
| 0 | 0 | chr5p15.33_amplification | 0 | 0 | 0 | 0 |
| 0 | 0 | chr5q31.1_deletion | 0 | 0 | 0 | 0 |
| 0 | 0 | chr5q35.3_amplification | 0 | 0 | 0 | 0 |
| 0 | 0 | chr6p21.1_amplification | 0 | 0 | 0 | 0 |
| 0 | 0 | chr7p22.3_amplification | 0 | 0 | 0 | 0 |
| 0 | 0 | chr8p21.1_amplification | 0 | 0 | 0 | 0 |
| 0 | 0 | chr8q24.3_deletion | 0 | 0 | 0 | 0 |
| 0 | 0 | chr9q33.3_amplification | 0 | 0 | 0 | 0 |
| 0 | 0 | chr9q34.11_amplification | 0 | 0 | 0 | 0 |

Supplementary Table 4. Cox regression analysis of CASC3 expression in the HER2-amplified TCGA-BRCA cohort.

| **Characteristics** | **Variable** | **Univariate Cox** | | | | **Multivariate Cox  (N=314)** |
| --- | --- | --- | --- | --- | --- | --- |
|  |  | **CASC3 high expression** | | **CASC3 low expression** | |  |
|  |  | **HR, 95% CI, P value** | **Number** | **HR, 95% CI, P value** | **Number** |  |
| Age | — | 1.05 (1.02-1.08, P<0.001) | 228 | 1.04 (1.00-1.07, P=0.025) | 221 | 1.04 (1.01-1.07, P=0.006) |
| Gender | Female | 1.00 | 221 | 1.00 | 221 | 1.00 |
|  | Male | 0.00 (0.00->1e+05, P=0.945) | 7 | — | 0 | — |
| Race | White | 1.00 | 150 | 1.00 | 149 | 1.00 |
|  | Black or African American | 1.43 (0.61-3.34, P=0.408) | 27 | 1.23 (0.46-3.28, P=0.682) | 43 | 2.82 (1.20-6.64, P=0.018) |
|  | Asian | 0.61 (0.08-4.58, P=0.633) | 20 | 0.78 (0.10-5.91, P=0.812) | 15 | 0.84 (0.11-6.48, P=0.868) |
| Stage | I-II | 1.00 | 163 | 1.00 | 157 | 1.00 |
|  | III-IV | 2.42 (1.23-4.76, P=0.011) | 52 | 2.29 (1.03-5.13, P=0.043) | 59 | 1.75 (0.62-4.94, P=0.292) |
| T_stage | T1 | 1.00 | 45 | 1.00 | 59 | 1.00 |
|  | T2 | 1.32 (0.43-4.01, P=0.625) | 144 | 1.03 (0.32-3.31, P=0.961) | 118 | 1.74 (0.69-4.38, P=0.238) |
|  | T3 | 5.10 (1.49-17.49, P=0.010) | 18 | 0.85 (0.15-4.68, P=0.850) | 28 | 2.39 (0.60-9.55, P=0.219) |
|  | T4 | 3.51 (0.77-16.11, P=0.106) | 10 | 2.43 (0.42-14.03, P=0.320) | 8 | 1.70 (0.32-8.95, P=0.531) |
| N_stage | N0 | 1.00 | 90 | 1.00 | 98 | 1.00 |
|  | N1-3 | 1.78 (0.78-4.07, P=0.173) | 121 | 0.95 (0.36-2.54, P=0.920) | 113 | 1.07 (0.48-2.37, P=0.868) |
| M_stage | M0 | 1.00 | 189 | 1.00 | 179 | 1.00 |
|  | M1 | 15.97 (4.58-55.67, P<0.001) | 4 | 4.66 (0.60-36.29, P=0.141) | 2 | 4.51 (0.77-26.33, P=0.094) |

HR, Hazard Ratio; CI, Confidence Interval; >1e+05, >100,000; —, not applicable.

Supplementary Table 5. Cox regression analysis of ILF2 expression in the HER2-amplified TCGA-BRCA cohort.

| **Characteristics** | **Variable** | **Univariate Cox** | | | | **Multivariate Cox  (N=314)** |
| --- | --- | --- | --- | --- | --- | --- |
|  |  | **ILF2 high expression** | | **ILF2 low expression** | |  |
|  |  | **HR, 95% CI, P value** | **Number** | **HR, 95% CI, P value** | **Number** |  |
| Age | — | 1.03 (1.00-1.06, P=0.033) | 152 | 1.06 (1.03-1.09, P=<0.001) | 297 | 1.04 (1.01-1.07, P=0.004) |
| Gender | Female | 1.00 | 150 | 1.00 | 292 | 1.00 |
|  | Male | 0.00 (0.00->1e+05, P=0.956) | 2 | 0.00 (0.00->1e+05, P=0.950) | 5 | — |
| Race | White | 1.00 | 90 | 1.00 | 209 | 1.00 |
|  | Black or African American | 1.95 (0.89-4.28, P=0.094) | 30 | 0.24 (0.03-1.79, P=0.166) | 40 | 2.16 (0.91-5.12, P=0.081) |
|  | Asian | 3.20 (0.67-15.24, P=0.145) | 16 | 0.00 (0.00->1e+05, P=0.933) | 19 | 0.84 (0.11-6.50, P=0.869) |
| Stage | I-II | 1.00 | 110 | 1.00 | 210 | 1.00 |
|  | III-IV | 1.90 (0.91-3.95, P=0.088) | 38 | 2.51 (1.20-5.21, P=0.014) | 73 | 1.42 (0.50-4.05, P=0.516) |
| T_stage | T1 | 1.00 | 22 | 1.00 | 82 | 1.00 |
|  | T2 | 0.47 (0.16-1.38, P=0.171) | 98 | 2.24 (0.64-7.88, P=0.208) | 164 | 1.61 (0.64-4.08, P=0.312) |
|  | T3 | 1.79 (0.47-6.79, P=0.390) | 12 | 3.25 (0.76-13.86, P=0.111) | 34 | 2.36 (0.57-9.81, P=0.239) |
|  | T4 | 0.94 (0.22-4.06, P=0.934) | 11 | 13.52 (2.23-81.99, P=0.005) | 7 | 1.01 (0.19-5.46, P=0.993) |
| N_stage | N0 | 1.00 | 69 | 1.00 | 119 | 1.00 |
|  | N1-3 | 1.15 (0.48-2.75, P=0.753) | 70 | 1.85 (0.72-4.73, P=0.200) | 164 | 1.24 (0.56-2.74, P=0.593) |
| M_stage | M0 | 1.00 | 123 | 1.00 | 245 | 1.00 |
|  | M1 | 27.17 (2.76-267.22, P=0.005) | 2 | 11.71 (3.32-41.28, P<0.001) | 4 | 6.51 (1.09-39.00, P=0.040) |

HR, Hazard Ratio; CI, Confidence Interval; >1e+05, >100,000; —, not applicable.

Supplementary Table 6. Cox regression analysis of C1orf43 expression in the HER2-amplified TCGA-BRCA cohort.

| **Characteristics** | **Variable** | **Univariate Cox** | | | | **Multivariate Cox  (N=314)** |
| --- | --- | --- | --- | --- | --- | --- |
|  |  | **C1orf43 high expression** | | **C1orf43 low expression** | |  |
|  |  | **HR (95% CI, P value)** | **Number** | **HR (95% CI, P value)** | **Number** |  |
| Age | — | 1.04 (1.02-1.06, P<0.001) | 369 | 1.12 (1.00-1.25, P=0.045) | 80 | 1.04 (1.01-1.07, P=0.008) |
| Gender | Female | 1.00 | 364 | 1.00 | 78 | 1.00 |
|  | Male | 0.00 (0.00->1e+05, P=0.934) | 5 | 0.00 (0.00->1e+05, P=0.983) | 2 | — |
| Race | White | 1.00 | 248 | 1.00 | 51 | 1.00 |
|  | Black or African American | 1.28 (0.66-2.49, P=0.464) | 56 | 0.00 (0.00->1e+05, P=0.980) | 14 | 2.69 (1.15-6.27, P=0.022) |
|  | Asian | 0.98 (0.23-4.07, P=0.972) | 27 | 0.00(0.00->1e+05, P=0.990) | 8 | 0.89 (0.12-6.83, P=0.909) |
| Stage | I-II | 1.00 | 259 | 1.00 | 61 | 1.00 |
|  | III-IV | 1.89 (1.11-3.22, P=0.020) | 96 | 5.50 (0.77-39.09, P=0.088) | 15 | 1.52 (0.53-4.34, P=0.433) |
| T_stage | T1 | 1.00 | 79 | 1.00 | 25 | 1.00 |
|  | T2 | 1.09 (0.48-2.45, P=0.837) | 215 | >1e+05 (0.00->1e+05, P=0.978) | 47 | 1.74 (0.71-4.27, P=0.226) |
|  | T3 | 1.99 (0.77-5.17, P=0.158) | 40 | 0.97 (0.00->1e+05, P=1.000) | 6 | 1.96 (0.48-7.94, P=0.346) |
|  | T4 | 2.21 (0.70-6.99, P=0.178) | 18 |  | 0 | 1.53 (0.29-8.08, P=0.619) |
| N_stage | N0 | 1.00 | 158 | 1.00 | 30 | 1.00 |
|  | N1-3 | 1.35 (0.70-2.59, P=0.374) | 186 | 1.65 (0.15-18.37, P=0.683) | 48 | 1.17 (0.53-2.58, P=0.696) |
| M_stage | M0 | 1.00 | 299 | 1.00 | 69 | 1.00 |
|  | M1 | 8.21 (2.87-23.43, P<0.001) | 6 | — | 0 | 3.18 (0.56-17.89, P=0.190) |

HR, Hazard Ratio; CI, Confidence Interval; >1e+05, >100,000; —, not applicable.

Supplementary Table 7. Cox regression analysis of HAX1 expression in the HER2-amplified TCGA-BRCA cohort.

| **Characteristics** | **Variable** | **Univariate Cox** | | | | **Multivariate Cox  (N=314)** |
| --- | --- | --- | --- | --- | --- | --- |
|  |  | **HAX1 high expression** | | **HAX1 low expression** | |  |
|  |  | **HR, 95% CI, P value** | **Number** | **HR, 95% CI, P value** | **Number** |  |
| Age | — | 1.01 (0.97-1.06, P=0.600) | 60 | 1.05 (1.03-1.08, P<0.001) | 389 | 1.03 (1.01-1.06, P=0.019) |
| Gender | Female | 1.00 | 59 | 1.00 | 383 | 1.00 |
|  | Male | 0.00 (0.00->1e+05, P=0.973) | 1 | 0.00 (0.00->1e+05, P=0.936) | 6 | — |
| Race | White | 1.00 | 37 | 1.00 | 262 | 1.00 |
|  | Black or African American | 3.40 (1.08-10.68, P=0.036) | 14 | 0.62 (0.22-1.74, P=0.365) | 56 | 2.42 (1.03-5.70, P=0.044) |
|  | Asian | 0.00 (0.00->1e+05, P=0.969) | 4 | 0.87 (0.21-3.63, P=0.850) | 31 | 0.88 (0.11-6.78, P=0.903) |
| Stage | I-II | 1.00 | 41 | 1.00 | 279 | 1.00 |
|  | III-IV | 4.50 (1.29-15.64, P=0.018) | 18 | 1.97 (1.09-3.55, P=0.024) | 93 | 1.77 (0.61-5.14, P=0.295) |
| T_stage | T1 | 1.00 | 11 | 1.00 | 93 | 1.00 |
|  | T2 | 0.54 (0.12-2.45, P=0.427) | 33 | 2.07 (0.71-6.07, P=0.183) | 229 | 1.87 (0.74-4.72, P=0.184) |
|  | T3 | 2.55 (0.53-12.22, P=0.242) | 10 | 2.63 (0.70-9.88, P=0.152) | 36 | 2.12 (0.52-8.60, P=0.292) |
|  | T4 | 2.58 (0.25-26.93, P=0.428) | 4 | 4.27 (1.02-17.88, P=0.047) | 14 | 1.81 (0.34-9.61, P=0.484) |
| N_stage | N0 | 1.00 | 28 | 1.00 | 160 | 1.00 |
|  | N1-3 | 1.06 (0.32-3.50, P=0.923) | 27 | 1.60 (0.76-3.40, P=0.219) | 207 | 1.04 (0.46-2.32, P=0.930) |
| M_stage | M0 | 1.00 | 49 | 1.00 | 319 | 1.00 |
|  | M1 | 21.86 (3.60-132.90, P<0.001) | 2 | 5.84 (1.37-24.88, P=0.017) | 4 | 3.52 (0.63-19.80, P=0.153) |

HR, Hazard Ratio; CI, Confidence Interval; >1e+05, >100,000; —, not applicable.

Supplementary Table 8. Cox regression analysis of S100A13 expression in the HER2-amplified TCGA-BRCA cohort.

| **Characteristics** | **Variable** | **Univariate Cox** | | | | **Multivariate Cox  (N=314)** |
| --- | --- | --- | --- | --- | --- | --- |
|  |  | **S100A13 high expression** | | **S100A13 low expression** | |  |
|  |  | **HR (95% CI, P value)** | **Number** | **HR (95% CI, P value)** | **Number** |  |
| Age | — | 1.05 (1.02-1.08, P<0.001) | 277 | 1.05 (1.02-1.08, P=0.002) | 172 | 1.04 (1.01-1.07, P=0.003) |
| Gender | Female | 1.00 | 272 | 1.00 | 170 | 1.00 |
|  | Male | 0.00 (0.00->1e+05, P=0.941) | 5 | 0.00 (0.00->1e+05, P=0.967) | 2 | — |
| Race | White | 1.00 | 181 | 1.00 | 118 | 1.00 |
|  | Black or African American | 1.86 (0.88-3.93, P=0.105) | 51 | 0.53 (0.12-2.23, P=0.384) | 19 | 2.59 (1.13-5.93, P=0.024) |
|  | Asian | 0.00 (0.00->1e+05, P=0.935) | 18 | 2.80 (0.62-12.70, P=0.181) | 17 | 0.94 (0.12-7.28, P=0.955) |
| Stage | I-II | 1.00 | 192 | 1.00 | 128 | 1.00 |
|  | III-IV | 3.43 (1.71-6.88, P=<0.001) | 74 | 1.05 (0.44-2.50, P=0.906) | 37 | 1.45 (0.51-4.17, P=0.489) |
| T_stage | T1 | 1.00 | 64 | 1.00 | 40 | 1.00 |
|  | T2 | 1.23 (0.40-3.80, P=0.721) | 158 | 1.35 (0.43-4.20, P=0.606) | 104 | 1.50 (0.61-3.65, P=0.374) |
|  | T3 | 3.16 (0.92-10.80, P=0.067) | 35 | 0.82 (0.09-7.45, P=0.863) | 11 | 2.15 (0.54-8.64, P=0.279) |
|  | T4 | 2.79 (0.58-13.40, P=0.200) | 10 | 4.48 (0.80-25.12, P=0.088) | 8 | 1.54 (0.27-8.79, P=0.627) |
| N_stage | N0 | 1.00 | 125 | 1.00 | 63 | 1.00 |
|  | N1-3 | 1.80 (0.78-4.17, P=0.168) | 138 | 0.98 (0.37-2.58, P=0.960) | 96 | 1.14 (0.52-2.53, P=0.741) |
| M_stage | M0 | 1.00 | 219 | 1.00 | 149 | 1.00 |
|  | M1 | 14.01 (3.98-49.39, P<0.001) | 5 | 3.38 (0.43-26.33, P=0.245) | 1 | 2.76 (0.43-17.56, P=0.283) |

HR, Hazard Ratio; CI, Confidence Interval; >1e+05, >100,000; —, not applicable.

Supplementary Material

Supplementary Figure 1. Canonical lineage marker genes.

Uniform manifold approximation and projection plots were colored by EPCAM for epithelial cells, PDGFR for fibroblasts, PECAM1 for endothelial cells, CD68 for myeloid cells,CD3D for T cellsand MS4A1 for B cells.

Supplementary Figure 2. Malignant epithelial cell classification based on inferred copy number variation scores.

A. Statistics of epithelial cell counts and tissue types across samples.

B. Correlation between copy number variation (CNV) deviation and epithelial cell number.

C. Association of CNV burden with tumor subtype.

D. Correlation between CNV burden and epithelial cell number.

E. For each patient, epithelial cells were stratified into two or three groups according to CNV scores. Malignant cells were identified by selecting those with the highest CNV scores within each sample, while remaining cells were categorized as unassigned.

Supplementary Figure 3. Heatmap of the whole epithelial cells or non-malignant cells from 14 single-cell sequenced patients.

A. Heatmap of the whole epithelial cells from 14 single-cell sequenced patients.

B. Heatmap of the non-malignant epithelial cells from 14 single-cell sequenced patients..

Supplementary Figure 4. Association between HER2 expression and copy number variation burden across all or malignant epithelial cells from 14 single-cell sequenced patients.

A. In the cohort of 14 HER2-amplified breast cancers, HER2 expression and copy number variation scores of all epithelial cells showed significant positive correlations in 13 patients (Pearson r = 0.1 - 0.7, p < 0.05), with only Patient08 as exception (r = −0.08).

B. Pearson correlation analysis revealed a significant association between HER2 expression and copy number variation scores in malignant epithelial cells from 14 HER2-amplified breast cancers (r = 0.07–0.3, p < 0.05), except in Patient08 (r = −0.06).

Supplementary Figure 5. Copy number variation-based phylogenetic reconstruction of tumor evolution in HER2 amplified breast cancer.

Phylogenetic analysis of Patient02-14 revealed clonal architectures and evolutionary trajectories through copy number variation (CNV) patterns. The accompanying heatmaps visualized chromosomal alterations (red: amplifications; blue: deletions) across all epithelial cells or specific malignant cell subsets, with columns representing genomic regions and rows indicating single-cell CNV profiles.

Supplementary Figure 6. Distribution of copy number variation scores in epithelial cells from 14 single-cell sequenced patients.

Supplementary Figure 7. Association of HER2 expression or epithelium score with copy number variation scores in spatially resolved malignant epithelial cells.

A.Analysis of 8 HER2-amplified breast cancer specimens demonstrated consistent positive correlations between HER2 expression levels and copy number variation scores across all patients (Pearson r = 0.1-0.5; p < 0.05) in spatially mapped epithelial cell populations.

B. Analysis of 8 HER2-amplified breast cancer specimens demonstrated consistent positive correlations between epithelium scores and copy number variation scores across all patients (Pearson r = 0.05-0.35; p < 0.05) in spatially mapped epithelial cell populations.

Supplementary Figure 8. Cell type annotation and epithelial CNV score mapping from spatial transcriptomic data. Spatial mapping of cell clusters in Patients A, G, and H illustrates the localization of distinct cell populations within the tissue architecture, alongside the corresponding distribution of epithelial CNV.

Supplementary Figure 9. Spatial transcriptomics analysis of Patient G delineated intratumoral heterogeneity and evolutionary subclonal dynamics.

A. Copy number variation (CNV) heatmap of spatial transcriptomics spots in Patient G. Columns show: chromosomal regions (x-axis), histology (DCIS: dodger blue; IDC: chartreuse), and clones (A-D; color-coded). Rows represent single cells with CNV magnitude (red: amplification; blue: deletion).

B. Tissue section mapping spots: (top) histological regions (DCIS/IDC); (bottom) spatially resolved subclones (A-D).

C. Phylogenetic reconstruction of clonal evolution. Node sizes reflect spot abundance; branch lengths indicate CNV divergence (log-scaled). Pie charts show DCIS/IDC composition per clone. "P" marks the common progenitor.

D. Pseudotime trajectory (Monocle) modeling DCIS to IDC progression with subclonal dynamics.

**E.** Two distinct progression models emerge in Patient G: the evolutionary bottleneck model (migration of clones C) and independent evolution model (emergence of clone D), originating from a common progenitor population.

Supplementary Figure 10. CNV frequency and survival analysis of CNV‑associated genes in HER2‑amplified breast cancer.

A. Comparative CNV frequencies between TCGA datasets and scRNA‑seq data.

B. Kaplan‑Meier plot depicting the impact of C1orf43 expression on overall survival in HER2‑amplified breast cancer patients.

C. Kaplan‑Meier analysis of survival stratified by HAX1 expression in HER2‑amplified breast cancer patients.

D. Kaplan‑Meier analysis of survival stratified by S100A13 expression in HER2‑amplified breast cancer patients.

Supplementary Table 1. Clinical and pathological information of all patients.

Supplementary Table 2. Tumor subclonal architecture reconstruction in Patient A via copy number variation profiling.

Supplementary Table 3. Clonal architecture deconvolution in Patient H using genome-wide copy number alteration patterns.

Supplementary Table 4. Cox regression analysis of CASC3 expression in the HER2-amplified TCGA-BRCA cohort.

Supplementary Table 5. Cox regression analysis of ILF2 expression in the HER2-amplified TCGA-BRCA cohort.

Supplementary Table 6. Cox regression analysis of C1orf43 expression in the HER2-amplified TCGA-BRCA cohort.

Supplementary Table 7. Cox regression analysis of HAX1 expression in the HER2-amplified TCGA-BRCA cohort.

Supplementary Table 8. Cox regression analysis of S100A13 expression in the HER2-amplified TCGA-BRCA cohort.
